# Epidemiology, patient outcome and complications after non‐operative management of hip fracture: a systematic review

**DOI:** 10.1111/anae.16732

**Published:** 2025-08-25

**Authors:** James Winfield, Lynn McNicoll, Iain K. Moppett

**Affiliations:** ^1^ Medical School University of Nottingham UK; ^2^ Anaesthesia and Critical Care Section, Academic Unit of Injury, Recovery and Inflammation Sciences University of Nottingham UK; ^3^ Alpert Medical School of Brown University Providence RI USA

**Keywords:** conservative, decision‐making, hip fracture, non‐operative

## Abstract

**Introduction:**

Surgery is the preferred treatment for hip fracture in older people. However, a proportion of patients with hip fracture do not receive surgery. There is a lack of contemporary evidence describing this patient population and what their associated outcomes are. We aimed to describe the variation in non‐operative management and its outcomes around the world.

**Methods:**

We performed a systematic review and meta‐analysis of older people presenting to hospital with hip fracture, comparing those with and without surgery for non‐operative proportions, mortality and other outcomes. Risk of bias was assessed using the Newcastle‐Ottawa Scale. We performed a random effects meta‐analysis with adjustment for clustering.

**Results:**

Of 4437 screened studies, 185 were included from 172 separate cohorts, 44 countries, six continents and involving 10,763,994 patients. The overall proportion of non‐operative management was 8.4% (95%CI 7.2–9.7%) with wide within‐country and regional variation. There was no consistent association of non‐operative management proportions with the admission characteristics of sex, fracture type or patient ethnicity. Non‐operative management was associated with a greater relative risk of death at all time points. Risk of bias was generally low except for the expected confounding by indication.

**Discussion:**

Non‐operative management of hip fracture is relatively common, but there is wide variation that is unexplained by differences in patient characteristics. The evidence is limited by incomplete reporting of patient characteristics and outcomes, and a lack of controlled studies even in the highest risk populations. Further work is needed to understand this decision‐making process.

## Plain Language Summary

Older people who break their hip are usually treated with surgery. But some people do not get surgery. We don't know much about who these people are or what happens to them. This study looked at how often people don't get surgery and what the results are in different countries. We looked at many past studies of older people who went to the hospital with a broken hip. We compared those who had surgery to those who did not. We checked how many people didn't have surgery, how many died and other health outcomes. We also checked how trustworthy each study was. We looked at 185 studies from 44 countries across six continents. These studies included over 10 million patients. On average, about 8 out of every 100 people with a hip fracture did not get surgery. This number was very different from country to country. Whether someone had surgery or not did not seem to depend on their sex, type of hip break or ethnic background. People who didn't have surgery were more likely to die at any time after their injury. Most studies were well done, but many did not explain why patients didn't have surgery. Not having surgery for a broken hip is quite common, but there are big differences in how often this happens in different places. These differences don't seem to be caused by patient health or other easy‐to‐see reasons. Many studies didn't give full details about the patients or their outcomes. More research is needed to understand why doctors sometimes choose not to do surgery.

## Introduction

Surgery is generally the preferred treatment for fragility hip fractures in the older person [1, 2, 3]. The surgical technique is well‐defined according to fracture type, and the beneficial role of early mobilisation and orthogeriatric models of care is well‐recognised [1, 2, 3]. The evidence in favour of surgery does not come from randomised clinical trials but from consensus‐driven change in practice and a recognition of the poor outcomes in the past of those patients managed non‐operatively or who underwent delayed surgery. People presenting with hip fracture are at high risk of poor outcomes due to pre‐existing comorbidities, advanced age and frailty, and there is limited evidence to guide when non‐operative management should be considered and what the outcomes are following non‐operative management in contemporary practice. Recent work from our group has suggested that there is significant variation globally in attitudes and practice towards non‐operative management of hip fracture [4].

Our objective was to review the published literature systematically to describe: the variation in rates of non‐operative management of hip fracture across the world; the differences in admission characteristics between the non‐operative and operative populations; the outcomes associated with non‐operative compared with operative management; and the evidence gaps in known outcomes. Our focus was as much on describing the variation as on providing point estimates of particular characteristics.

## Methods

We performed a systematic review and meta‐analysis. Minor changes to the pre‐registered protocol [5] included: a decision to limit included references/reports to those with mid‐cohort date after 1995 (to describe more contemporary practice); exclusion of studies reporting comparisons of management approaches for impacted intracapsular fractures (not the population of interest); and exclusion of COVID‐19‐related studies (non‐representative of general practice and reported widely elsewhere). This study has been reported in line with PRISMA recommendations.

We primarily searched PubMed and Web of Science (online Supporting Information Appendix S1). These searches were supplemented with specific searches for reports from hip fracture registries, keyword searching within Google Scholar and Google, snowballing forwards (citing references within PubMed and Google Scholar) and backwards (manual review of reference lists). The primary search was carried out in February 2024; updates were carried out, including snowballing, in June 2024 and October 2024.

Inclusion criteria were: study population of patients with fragility hip fracture requiring hospital care (as defined by the author or clearly corresponding to international definitions); and comparative data for both operative and non‐operative patients for at least one characteristic, including their respective numbers or proportions. Key exclusion criteria were: studies before 1995; studies investigating surgical approaches to impacted intracapsular hip fracture; and COVID‐19‐related studies. There was no restriction by country, study type (trials, observational studies) fracture type or publication language. We did not attempt to assess rates of non‐operative management formally across whole populations.

Search results were de‐duplicated using a combination of automated matching and manual verification. All screening, abstract, full paper review, quality assessment and data entry were performed independently by two study authors, with resolution of differences by discussion and/or correction from original data. We used Rayyan (Rayyan Systems Inc., Cambridge, MA, USA) for study screening and deduplication; Covidence (Covidence, Melbourne, Australia) for full text review; and Microsoft Excel (Microsoft Corporation, Redmond, WA, USA) for data collation.

Study quality was assessed using the Newcastle‐Ottawa scale [6]. With the exception of matched cohort studies, all studies were expected to be substantially confounded by indication. Data extraction items included study details; reasons (if stated) for non‐operative management; numbers of non‐operative and operative patients; characteristics of the non‐operative and operative cohorts (e.g. age, sex, fracture type); and outcomes of the non‐operative and operative cohorts (e.g. mortality at various time‐points; complications). Absence of reporting of characteristics and outcomes was documented for all studies. Ethnicity was reported by reference to the majority ethnic group of the relevant country. We did not seek information on funding sources as this was highly unlikely to be of relevance.

A priori cohorts were categorised by World Bank region [7] (East Asia and Pacific; Europe and Central Asia; Latin America and the Caribbean; Middle East and North Africa; North America; South Asia; Sub‐Saharan Africa); World Bank classification of income; unselected (all people with hip fracture without stratification by risk); lower vs. higher underlying risk; and source of data (local collection; administrative data; registry). Underlying risk was assigned based on descriptions of cohort age (age < or > 85 y; approximately the median age of people with hip fracture); dementia; frailty; non‐home residence before fracture; or authors' description and agreed by two authors.

Multiple reports from the same or closely time‐overlapping cohorts were collapsed where possible into single datasets. Conversely, where cohorts could be separated clearly (e.g. age groups described separately) these were reported separately, whilst retaining an overall cohort identifier. Data were extracted or calculated directly from the published data. Estimates of absolute values were used if proportions were reported and where necessary data were extracted from graphs using electronic callipers. Standard deviations were estimated from ranges using established methods [8].

Meta‐analysis was undertaken using R (Darwin, 21.6.0, R Core Team, Vienna, Austria) within R studio, with the packages meta and metafor. Sensitivity analyses were performed using the metafor package and the impact of clustering (by country or region) was investigated. Results are reported as proportion estimates (proportion having non‐operative management), risk ratios (categorical population characteristics and outcomes) or mean differences (continuous characteristics and outcomes) as appropriate. Random effects were assumed throughout. Statistical heterogeneity was reported using *I* ^2^ statistic for completeness; however, we anticipated a high *I* ^2^ due to the differences in study locations, times and populations [9].

The co‐primary outcomes were: a qualitative geographical description of the coverage of reported data regarding the population having non‐operative management and the outcomes reported; a quantitative description of differences between the non‐operative and operative hip fracture populations; and a quantitative description of the differences in outcomes between the non‐operative and operative hip fracture populations.

## Results

**Figure 1:**
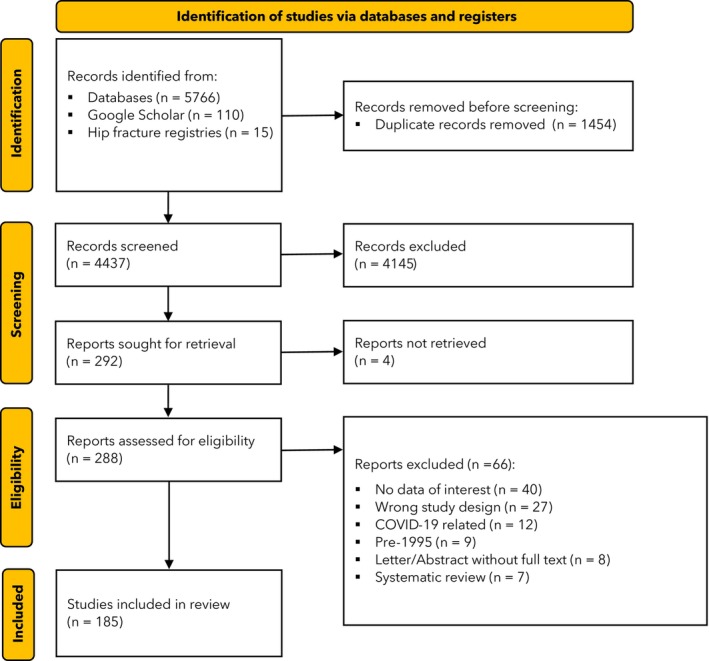
Study flow diagram.

The search initially identified 5891 records (Fig. 1) from which 185 studies were included from 172 separate cohorts, 44 countries and six continents (online Supporting information Figures S1 and S2), and involved 10,763,994 patients (online Supporting Information Tables S1–S3). The term cohort cluster is used to describe the combined studies, though in most instances these were single reports. All studies identified by three recent systematic reviews [10, 11, 12] were included except Rashidifard et al. [13], which contained data only on non‐operative management, and Jain et al. [14], which was pre‐1995. There were 14 registries with usable data [15, 16, 17, 18, 19, 20, 21, 22, 23, 24, 25, 26, 27, 28, 29, 30, 31, 32, 33, 34, 35]; 34 cohorts based on national administrative data [36, 37, 38, 39, 40, 41, 42, 43, 44, 45, 46, 47, 48, 49, 50, 51, 52, 53, 54, 55, 56, 57, 58, 59, 60, 61, 62, 63, 64, 65, 66, 67, 68, 69]; 18 multicentre cohorts [70, 71, 72, 73, 74, 75, 76, 77, 78, 79, 80, 81, 82, 83, 84, 85, 86, 87, 88, 89]; and 100 single centre studies [90, 91, 92, 93, 94, 95, 96, 97, 98, 99, 100, 101, 102, 103, 104, 105, 106, 107, 108, 109, 110, 111, 112, 113, 114, 115, 116, 117, 118, 119, 120, 121, 122, 123, 124, 125, 126, 127, 128, 129, 130, 131, 132, 133, 134, 135, 136, 137, 138, 139, 140, 141, 142, 143, 144, 145, 146, 147, 148, 149, 150, 151, 152, 153, 154, 155, 156, 157, 158, 159, 160, 161, 162, 163, 164, 165, 166, 167, 168, 169, 170, 171, 172, 173, 174, 175, 176, 177, 178, 179, 180, 181, 182, 183, 184, 185, 186, 187, 188, 189, 190, 191, 192, 193, 194, 195, 196, 197, 198, 199, 200].

Risk of bias assessments are reported in online Supporting information Figures S3–S6. Risk of bias was generally low except for the expected confounding by indication. Of the outcomes reported, mortality and duration of hospital stay were at low risk of bias. Complications, including delirium, were generally at higher risk due to lack of formalised data collection or standardised definitions. Reporting of population characteristics was highly variable across cohorts. All included studies reported the proportions of patients with non‐operative vs. operative management (online Supporting information Figure S7). Age (n = 44), sex (n = 43) and fracture type (n = 41) were the most reported patient characteristics, followed by some measure of comorbidity (n = 21) and presence of dementia (n = 20). All other characteristics were reported by < 1 in 10 included cohorts. Ethnicity was reported by eight cohorts.

Reporting of outcomes was variable, reported by 87 cohort clusters (online Supporting information Figure S8). Mortality in some form was reported by 78 cohort clusters. One‐year (n = 50) and 30‐day mortality (n = 34) were the time points reported most commonly. Duration of hospital stay (n = 21) and complications (any) (15) were next most frequent, with all other outcomes reported by < 10 cohort clusters (online Supporting Information Figure S8).

**Figure 2:**
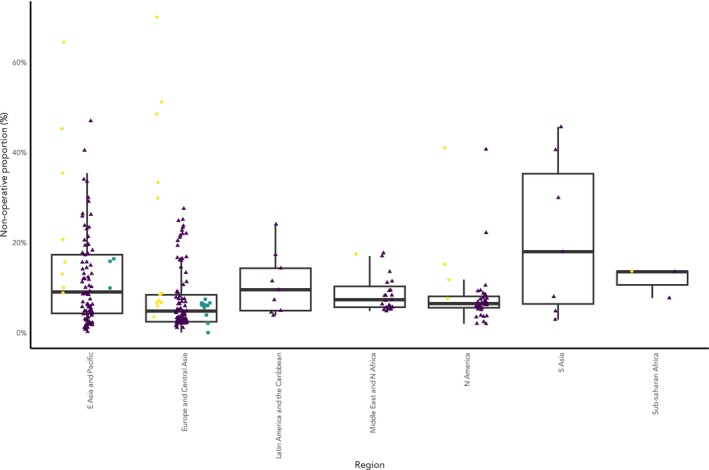
Distribution of proportions undergoing non‐operative management according to world bank region and cohort risk (higher risk, yellow circles; unselected, purple triangles; lower risk, teal squares). The boxes represent the interquartile range (IQR), with the median as a thicker line. Whiskers: The upper whisker extends from the 1st or 3rd quartile to the largest value no further than 1.5 × IQR from the quartile.

**Figure 3:**
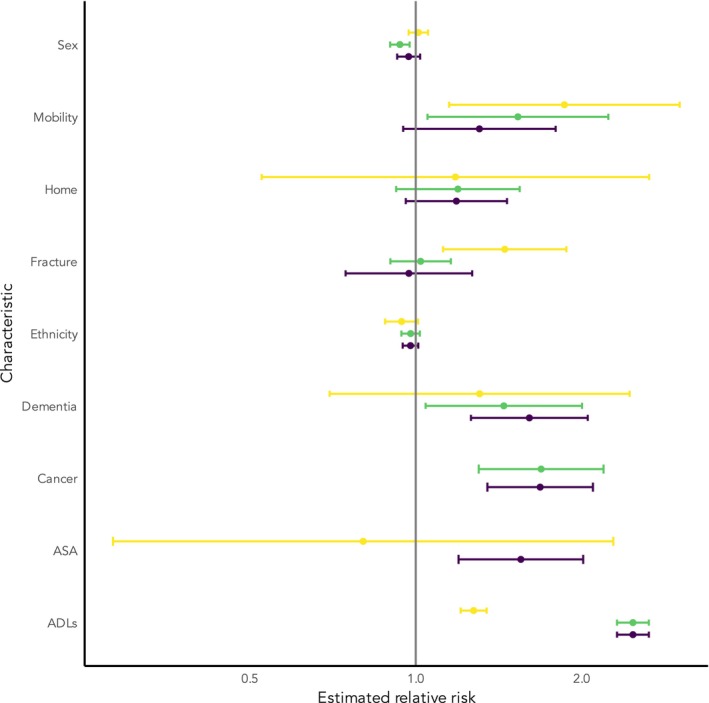
Estimated relative risk of receiving non‐operative management. Reference groups: sex, female; mobility, independently mobile; home, admitted from home; fracture, intracapsular fracture; ethnicity, majority ethnicity; dementia, no dementia; cancer, no cancer; ASA physical status, ASA physical status 1 or 2; ADLs, independent of activities of daily living. Unselected: cohorts which include all patients presenting with hip fracture (purple); administrative/registry: cohorts derived from prospectively gathered regional/national registries or from routine administrative datasets (green); high risk: cohorts where the population has been stratified by one or more risk factors for expected worse outcome (e.g. greater age, presence of dementia) (yellow).

The overall proportion of patients undergoing non‐operative management in unselected cohorts globally was 8.4% (95%CI 7.2–9.7%, 144 cohorts, 262 studies) (Fig. 3 and online Supporting Information Figure S9), and this proportion was essentially unchanged following sensitivity analyses and analysis with cohorts solely from administrative data (10.1% (95%CI 7.7–13.2%), 30 cohorts, 74 studies). Proportions were lower in data solely from registry cohorts (3.6% (95%CI 2.9–4.4%), 14 cohorts, 71 studies) and greater in higher risk cohorts (19% (13–27%), 25 cohorts, 27 studies). There was significant geographic variation (Fig. 2). There was variation in reported non‐operative rates for unselected populations within regions (interquartile ranges: East Asia and Pacific 11.8%; Europe and Central Asia 6.0%; Latin America and The Caribbean 9.4%; Middle East and North Africa 3.8%; North America 2.2%; South Asia 28.9%; Sub‐Saharan Africa 2.9%). For the 16 countries with more than five cohorts reported, the median interquartile range was 4.7% (overall range: < 0.3% (Northern Ireland) to 10.1% (Thailand)) (Fig. 2). There was no clear relationship between the proportion receiving non‐operative management and either the average age of the patients within the cohort, time period or proportion of the cohort with a diagnosis of dementia (online Supporting information Figures S10–S14).

There was no consistent association of non‐operative management proportions with the admission characteristics of sex, fracture type or ethnicity. Patients managed non‐operatively were aged on average approximately 2.6 (95%CI 1.7–3.6) years older than those undergoing surgery and their mean Charlson comorbidity index score was 0.5 (95% CI ‐0.2–0.8) points higher. Higher ASA physical status (3–5, relative risk 1.6, 95%CI 1.2–2.0), non‐independence of activities of daily living (relative risk 2.5 95%CI 2.3–2.7), cancer diagnosis (relative risk 1.7, 95%CI 1.4–2.1) and dementia (relative risk 1.6, 95%CI 1.3–2.1) were all associated with an increased risk of non‐operative management. These patterns were similar in data from administrative data or registries and in cohorts classified as high risk (Fig. 3). Being non‐independently mobile was associated with non‐operative management in administrative data/registries and high‐risk cohorts, but not in unselected cohorts overall (relative risk 1.3, 95%CI 0.95–1.8).

The overall estimate of 30‐day mortality for those patients receiving non‐operative management was 18% (95%CI 0.14–0.23) for the unselected cohort and 34% (95%CI 0.13–0.63) for the high‐risk cohort. One‐year mortality for non‐operative management was 46% (95% CI 0.41–0.51) for the unselected cohort and 65% (95%CI 0.39–0.85) for the high‐risk cohort. Non‐operative management was associated with greater relative risk of death at all time‐points (Table 1). Mortality outcomes for those undergoing non‐operative management were not associated with overall cohort mortality in unselected populations (online Supporting Figures S15 and S16).

**Table 1:** Summary of key outcomes. Summary estimates are provided with 95%CI as mean difference or relative risk, as appropriate; more negative (mean difference) or relative risk < 1 favours non‐operative management. Further details are provided in online Supporting Information Figures S20–S26.

Outcome	Group	n	Non‐operative		Operative		Summary estimate (95%CI)
			Events	Total	Events	Total	
30‐day mortality	All	32	27,889	169,148	35,899	46,4640	3.46 (2.58–4.65)
	Matched	4	117	320	42	527	4.50 (1.87–10.85)
	Higher risk	11	889	23,465	3347	34,846	2.40 (1.18–4.89)
	Unselected	17	26,883	145,363	32,510	42,9267	3.78 (3.1–4.63)
One‐year mortality	All	49	11,136	24,680	43,849	184,134	2.40 (2.11–2.74)
	Matched	5	212	348	167	583	2.17 (1.49–3.16)
	Higher risk	13	579	1099	2442	8283	1.71 (1.37–2.14)
	Unselected	31	10,345	23,233	41,240	175,268	2.77 (2.37–3.25)
Complications	All	6	182	1007	234	2623	1.73 (1.19–2.52)
	Higher risk	3	48	93	68	261	1.58 (1.02–2.46)
	Unselected	3	134	914	166	2362	1.82 (0.84–3.93)
Delirium	All	5	3760	23,549	5588	34,158	0.74 (0.48–1.15)
	Matched	1	14	94	18	114	0.94 (0.5–1.79)
	Higher risk	3	3734	23,222	5484	33,263	0.78 (0.4–1.52)
	Unselected	1	12	233	86	781	0.47 (0.26–0.84)
Pressure sores	All	6	123	1461	266	4918	1.46 (0.93–2.3)
	Higher risk	4	101	661	243	2782	1.13 (0.81–1.58)
	Unselected	2	22	800	23	2136	2.59 (0.77–8.68)
Duration of hospital stay; days	All	11	NA	1300	NA	4659	‐2.90 (‐5.14 to ‐0.65)
	Matched	2	NA	115	NA	134	0.09 (‐1.88–2.07)
	Higher risk	5	NA	229	NA	409	‐6.69 (‐12.19 to ‐1.19)
	Unselected	4	NA	956	NA	4116	‐1.74 (‐2.4 to ‐1.07)

NA, not applicable.

**Figure 4:**
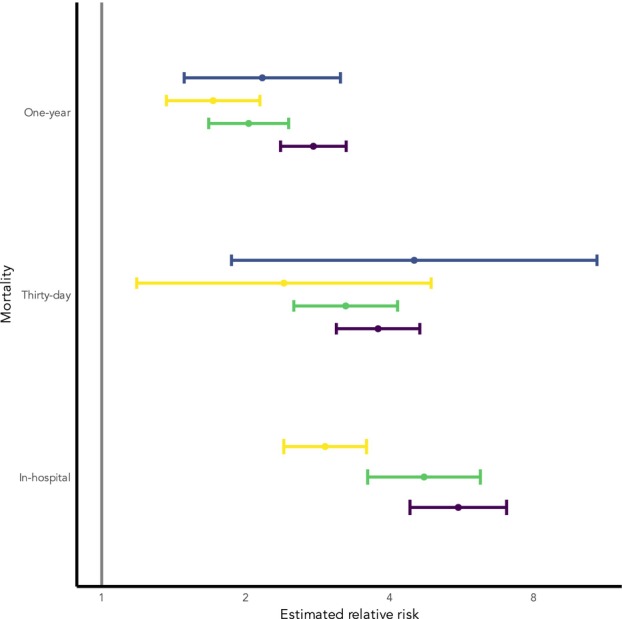
Relative risk of mortality associated with non‐operative management. Unselected: cohorts which include all patients presenting with hip fracture (purple); administrative/registry: cohorts derived from prospectively gathered regional/national registries or from routine administrative datasets (green); high risk: cohorts where the population has been stratified by one or more risk factors for expected worse outcome (e.g. greater age, presence of dementia) (yellow); matched: cohorts with control (operative) populations matched on key factors (e.g. age, comorbidities) (blue).

The relative risk of mortality decreased with increasing time since fracture (Fig. 4). Risks for overall complications and pressure injury for those undergoing non‐operative management were higher but this was not statistically significant. A small number of studies with data reported there was no significant difference in the risk of delirium or pressure injury in those with non‐operative management, but the number of studies was small (online Supporting Information Figure S17). Mean duration of hospital stay was 1.8 (95% CI 1.1–2.4) days shorter in those having non‐operative management (Table 1). Full reporting of the data is available in the online Supporting Information (forest plots Figures S18–S26; key study data Tables S4–S28; sensitivity analyses Tables S29–S31).

## Discussion

The key findings of our systematic review were: rates of and outcomes following non‐operative management of hip fracture vary across the world; despite non‐operative management being reported for around 1 in 12 patients, there is a paucity of data on who these people are; and there are large gaps in our understanding of what outcomes are for these patients, particularly for those who survive.

To our knowledge, this is the largest collation of data pertaining to non‐operative management of hip fracture. Three previous reviews identified only a total of 22 studies [8, 9, 10]. We cast a wide net deliberately, rather than narrowing our search to trials or matched cohort studies. We set out to describe variation in practice as much as deriving point estimates. Confounding by indication is universal to all the data we have reported; hence, interpreting outcomes of those with and without surgery is not straightforward. Any discussion of the point estimates we have generated must acknowledge the high degree of heterogeneity between reports and their clinical contexts. Although we have provided region‐level summary estimates, even those cover widely different healthcare systems and cultural approaches.

The variation in the use of non‐operative management of hip fracture between continents, countries and even within countries is significant (e.g. National Hip Fracture Database (NHFD) [28], Spanish Hip Fracture Registry [26] and Australia and New Zealand Hip Fracture Registry (ANZHFR) [13]) and is unlikely to be explained by differences in the patient populations. Our previous work suggests that there are regional and cultural differences in non‐operative decision‐making [4] and pathophysiological reasons are clearly a part of this. However, although the average age of patients managed non‐operatively was greater than those undergoing surgery, the difference was relatively small (2–2.5 years) and there was no association between the average age of the cohorts and proportions with non‐operative management. Similarly, although other surrogates of frailty such as independence in mobility and activities of daily living were suggestive of people living with greater degrees of frailty having non‐operative management, the data do not support this as a strong differentiator. The reasons why non‐operative management was chosen were frequently not reported or simply described in terms akin to ‘too unwell’. However, we suggest that it is likely the clinician‐patient‐family interpretation of the patient's current state and future outcomes influences this decision more than differences in the health of the patients presenting with hip fracture.

The small positive association between the proportion of the cohort with dementia (at least partially as a surrogate for greater frailty) and the proportion undergoing non‐operative management makes clinical sense but is too small to account for the variations. The extent to which biomechanical reasons (e.g. stable or old fractures) are a reason for variation in non‐operative management rates is also unclear. Data from the NHFD and ANZHFR show considerable between hospital/within‐country variation in reasons for non‐operative management. In addition, our previous work [4], and informal conversations with clinical colleagues, support the notion that external factors (e.g. perceived legal or reputational risk) and cultural factors (healthcare culture and societal expectations) may play at least a part in the decision to offer surgery.

The lack of data is startling. To our surprise, Europe appears relatively light on population and outcome data despite a strong tradition of hip fracture registries. Personal communications with some of the registries would suggest that more data are held than currently published and there are active projects to analyse data further. The lack of data from most of Africa is unsurprising; the number of people at risk of hip fracture is still relatively low, although demographic changes mean this will change in the next few decades.

As non‐randomised cohorts, all the studies are at considerable risk of bias by indication. In addition, aside from some of the national registries which can claim almost 100% capture of hip fracture data in their countries, there will inevitably be some degree of selection bias of the centres reporting data. The extent to which they serve populations that are different to the rest of their country or provide different approaches to non‐operative decision‐making is unanswerable. In many countries, almost all people with hip fractures will present to secondary care so reported data are likely to be representative of the local, if not national, population. In other parts of the world there is evidence that significant numbers either do not present to hospital or are not considered eligible for admission [201, 202, 203] for a variety of reasons and will therefore be missing from any data presented here. For those countries, the proportions provided with operative management will be over‐estimated. The reasons why registry‐based data give lower estimates of rates of non‐operative management are unclear. This may be simply due to where the registries are based, or it may reflect under‐reporting by registries or over‐reporting by administrative data. It is certainly likely that registries miss some patients that never come to their attention. However, this does not seem likely to account for the large differences seen, notably between the UK NHFD (registry) data (with non‐operative rates of around 2%) and the administrative UK data for the same periods reported by Burrack et al. [39] with rates of around 10%.

Even when sufficient data are available for non‐operative management, details of how these people compare with those offered surgery are sparse. Age, sex and fracture type are straightforward to collect, although the data we have would suggest that fracture type is irrelevant (with the caveat that we explicitly excluded studies focusing on non‐displaced intracapsular fractures). The limited data reported suggest that pre‐fracture functional status is associated with decisions for non‐operative management, as it is with postoperative rehabilitation outcomes [204]. These are important factors that would be considered as part of shared decision‐making. Without these data it is very hard to understand the variation in non‐operative management across the world. Importantly, there was very little information about expected prognosis or life expectancy after hip fracture and what options (e.g. hospice or palliative care) were available. These are important considerations in decision‐making. Without these data, it will be difficult going forward to understand when or if non‐operative management is a reasonable approach.

Outcome data are dominated by mortality. Unsurprisingly, mortality is higher at all time points with non‐operative management. This increase in associated risk is present in both unselected and higher‐risk cohorts, and is not associated with the underlying mortality risk of the cohort. In the absence of randomised trials, it remains impossible to say to what extent this mortality risk is due to identifying patients at the end of their life vs. non‐operative management processes resulting in a greater likelihood of death. We would suggest that both are contributors. The seminal work from Loggers et al. [11] in the Netherlands would suggest that, in their context, for the very frail, non‐operative management leads to a greater risk of death [81, 82, 83]. Conversely, the global variation in survival after non‐operative management would suggest that this is not a given, and a reasonable number of people receiving non‐operative management will survive beyond a year. There are scarcely any data to draw any meaningful conclusions about what survivorship looks like in the short or longer term. Surprisingly, there was no clear association between non‐operative management and delirium or pressure sores, which are generally assumed to be more common with untreated fracture, though again the data are sparse. However, outside of prospective data collection, we would argue that any data on delirium are treated sceptically. Duration of hospital stay appears to be shorter, likely as a result of both the competing risk of death and decisions to not attempt rehabilitation.

We draw a perhaps uncomfortable contrast with the wealth of information and research conducted into surgical options for hip fracture. We question whether it is ethical to continue to make decisions about 8% of the hip fracture population based on clinical wisdom and tradition alone. Notably, long‐term outcomes for survivors – quality of life, complications of poor mobility, impact on carers and family – are essentially absent. Successful discharge from hospital may not equate to a good outcome longer term. Currently, the minimum core dataset for hip fracture registries is unclear on which data should be reported for those undergoing non‐operative management [205, 206].

The drivers for surgery for patients with a hip fracture, even in those with high peri‐operative risk, come from observations that operating facilitates early mobilisation and is associated with a lower risk of death. In addition, even in the most acutely unwell or the most frail, the time between fracture and death is unpredictable [23] and operating is believed to support dignity and pain management, even if death is inevitable.

However, death is not the only outcome that matters to people with hip fracture [207, 208]. For some, they or their proxies may view hip fracture as a terminal event, whereby palliation of symptoms, not prolongation of life, is paramount. Contemporary nursing and medical care are very different now to the approaches of conservative management before surgery became the preferred option. Again, emphasising the particular social context, the FRAIL‐HIP study supports high satisfaction, despite very high early mortality when non‐operative management is chosen in people living with varying degrees of frailty [81, 82, 83].

Cost is a factor in all healthcare systems. We found few data addressing the relative costs of non‐operative compared with operative management. It is also likely context‐sensitive to the local setting. The only ‘guaranteed’ difference is that those not having surgery will avoid direct or indirect costs of implants and surgical time. Thereafter, it becomes more complex. Based on the limited data available, the duration of hospital stay is shorter, which would suggest lower total costs for non‐operative management, but potentially at the risk of transferring costs to other parts of the healthcare service. There was certainly some evidence of the significant cost (that could not always be borne) to families of accepting operative management for an older relative.

Our study has limitations. Despite a comprehensive search, we may have missed some reports; but additional data are unlikely to alter our key findings. Many of the studies reported non‐operative management as a byline of people not included in a study of postoperative outcomes, hence the lack of detailed information about the patients. There may be smaller studies that by chance had zero non‐operative management rates but did not report this explicitly, so were not captured in our searches. However, the lack of difference between data solely from administrative and registry‐based studies and results from all the cohorts would suggest the impact of this is small. We have undertaken extensive sensitivity analyses assessing the impact of outliers and there are no meaningful differences in results. Importantly, these data are not from randomised trials. We do not believe the data support a particular optimal proportion of non‐operative management. Personal and societal attitudes towards quantity and quality of life and ageing will differ. We do suggest, however, that there is unexplained and probably unwarranted variation.

Moving forward, we suggest that there are practical steps that may improve our understanding and practice in this area. The Fragility Fracture Network and individual registries could consider explicit inclusion of data fields related to characteristics and outcomes of those having non‐operative management. There is opportunity for national, regional and international consensus on the principles of decision‐making and pathways of care for those offered or receiving non‐operative management. We would suggest that these discussions must include patients and their carers, wider society and regulatory authorities. We must avoid any retreat into a nihilistic view of hip fracture management in general, which is rightly predicated on offering surgery to the vast majority of patients.

Bar the FRAIL‐Hip studies, there were few data on what non‐operative care looked like. In settings where non‐operative management is more common, we would suggest that this pathway is similar in quality to an operative pathway. There is scope for carefully considered research in this area which might include: identifying patients where non‐operative management may be the preferred option for them and their carers, either before or after fracture; understanding the reasons for inter‐individual, inter‐hospital and national variation; identifying optimal approaches to palliation in those who are dying; and rehabilitation in those where operative management is viewed as riskier than not operating.

In summary, non‐operative management of hip fracture is relatively common, proportions vary between and within countries, and this is not explained by differences in the populations. There is a distinct lack of data on who receives non‐operative management and what their outcomes are, especially for those who survive.

## Supporting information


**Plain Language Summary**.

**Appendix S1.** Search strategy.

**Figure S1.** Map of distribution of cohorts with characteristics data by country.
**Figure S2.** Map of distribution of cohorts with outcome data by country.
**Figure S3.** Risk of bias tables for cohort characteristics.
**Figure S4.** Risk of bias for mortality outcomes.
**Figure S5.** Risk of bias for delirium outcomes.
**Figure S6.** Risk of bias for pressure sore outcomes.
**Figure S7.** Completeness of characteristics reporting.
**Figure S8.** Completeness of outcome reporting.
**Figure S9.** Proportion of patients undergoing non‐operative management.
**Figure S10.** Age vs. proportion receiving non‐operative management.
**Figure S11.** Age vs. proportion receiving non‐operative management by World Bank Region.
**Figure S12.** Median date of cohort vs. proportion receiving non‐operative management.
**Figure S13.** Median date of cohort vs. proportion receiving non‐operative management by World Bank Region.
**Figure S14.** Dementia vs. proportion receiving non‐operative management.
**Figure S15.** Non‐operative proportion vs. 30‐day mortality.
**Figure S16.** Non‐operative proportion vs. 1‐year mortality.
**Figure S17.** Relative risk of delirium, pressure sores and complications with non‐operative management.
**Figure S18.** Risk ratio for female vs. non‐operative management.
**Figure S19.** Mean difference in age vs. non‐operative management.
**Figure S20.** Risk ratio for ASA physical status vs. non‐operative management.
**Figure S21.** Risk ratio for fracture type vs. non‐operative management.
**Figure S22.** Risk ratio for admission from own home vs. non‐operative management.
**Figure S23.** Mean difference in duration of hospital stay vs. non‐operative management.
**Figure S24.** Risk ratio for delirium vs. non‐operative management.
**Figure S25.** Risk ratio for pressure sores vs. non‐operative management.
**Figure S26.** Risk ratio for complications vs. non‐operative management

**Table S1.** Country cohort data.
**Table S2.** Study characteristics.
**Table S3.** Non‐operative proportions reported by study.
**Table S4.** Overall characteristics of included patients.
**Table S5.** Age: summary of age of patients.
**Table S6.** Sex: proportion of female patients.
**Table S7.** ASA physical status: proportion of ASA physical status three to five patients.
**Table S8.** Comorbidity: summary statistics for reported Charlson Comorbidity Index.
**Table S9.** Proportion of patients with dementia: proportion of patients with dementia.
**Table S10.** Proportion of patients with cancer.
**Table S11.** Proportion of patients admitted from home.
**Table S12.** Ambulatory status on admission: proportion of patients independently mobile.
**Table S13.** Activities of daily living: proportion of patients independent in ADLs.
**Table S14.** Ethnicity: proportion with majority ethnicity for the relevant country by study.
**Table S15.** Fracture type: proportion of patients with intracapsular fracture.
**Table S16.** In‐hospital mortality incidence.
**Table S17.** 7‐day mortality incidence.
**Table S18.** 30‐day mortality incidence.
**Table S19.** 90‐day mortality incidence.
**Table S20.** 180‐day mortality incidence.
**Table S21.** 1‐year mortality incidence.
**Table S22.** 2‐year mortality incidence.
**Table S23.** 3‐year mortality incidence.
**Table S24.** 5‐year mortality incidence.
**Table S25.** Duration of hospital stay.
**Table S26.** Discharge destination: proportion of patients returning to own home or nursing home.
**Table S27.** Ambulatory status at discharge.
**Table S28.** Delirium: proportion of patients with delirium.
**Table S29.** Sensitivity analyses for proportions of patients receiving non‐operative management.
**Table S30.** Sensitivity analyses for patient characteristics.
**Table S31.** Sensitivity analyses for outcomes (mortality, length of hospital stay, ambulatory status at discharge, delirium and complications).
