# Supplementary material for: Epidemiology, patient outcome and complications after non‐operative management of hip fracture: a systematic review
Source: Anaesthesia. 2025 Aug 25;80(11):1397–413. doi: 10.1111/anae.16732 (PMC12519934; doi:10.1111/anae.16732)
Supplement: Supplementary file 1 — Plain Language Summary. [file ANAE-80-1397-s002.docx]

**Plain Language Summary**

Older people who break their hip are usually treated with surgery. But some people do not get surgery. We don’t know much about who these people are or what happens to them. This study looked at how often people don’t get surgery and what the results are in different countries. We looked at many past studies of older people who went to the hospital with a broken hip. We compared those who had surgery to those who did not. We checked how many people didn’t have surgery, how many died and other health outcomes. We also checked how trustworthy each study was. We looked at 185 studies from 44 countries across six continents. These studies included over 10 million patients. On average, about 8 out of every 100 people with a hip fracture did not get surgery. This number was very different from country to country. Whether someone had surgery or not did not seem to depend on their sex, type of hip break or ethnic background. People who didn’t have surgery were more likely to die at any time after their injury. Most studies were well done, but many did not explain why patients didn’t have surgery. Not having surgery for a broken hip is quite common, but there are big differences in how often this happens in different places. These differences don’t seem to be caused by patient health or other easy to see reasons. Many studies didn’t give full details about the patients or their outcomes. More research is needed to understand why doctors sometimes choose not to do surgery.
