# Supplementary material for: Epidemiology, patient outcome and complications after non‐operative management of hip fracture: a systematic review
Source: Anaesthesia. 2025 Aug 25;80(11):1397–413. doi: 10.1111/anae.16732 (PMC12519934; doi:10.1111/anae.16732)
Supplement: Supplementary file 2 — Appendix S1. Search strategy. [file ANAE-80-1397-s004.docx]

# **Appendix S1 Search strategy**

### PubMed

((trochanter*) OR (subcapital OR sub-capital) OR (neck AND femur) OR (neck AND femor*) OR (hip) OR (intracapsul*) OR (extracapsul*) OR (basi-cervical OR basicervical) OR (transcervical OR trans-cervical) OR ((femur* OR femor*) AND (proxim*)) AND (fracture* OR trauma* OR break* OR broke* OR crack* OR fracture) OR (Hip fracture) OR (femoral neck fracture) OR (neck of femur fracture)) AND ((nonoperative) OR (non-operative) OR (non-operative*) OR (conservative) OR (conservativ*))

### Web of Science

(((ALL=(hip fracture) OR ALL=(proximal femoral fracture) OR ALL=(neck of femur fracture) OR ALL=(femoral neck fracture)))) AND ALL=((nonoperative OR non-operative OR conservative OR nonop OR non-op))
