# Supplementary material for: Epidemiology, patient outcome and complications after non‐operative management of hip fracture: a systematic review
Source: Anaesthesia. 2025 Aug 25;80(11):1397–413. doi: 10.1111/anae.16732 (PMC12519934; doi:10.1111/anae.16732)
Supplement: Supplementary file 3 — Figure S1. Map of distribution of cohorts with characteristics data by country. Figure S2. Map of distribution of cohorts with outcome data by country. Figure S3. Risk of bias tables for cohort characteristics. Figure S4. Risk of bias for mortality outcomes. Figure S5. Risk of bias for delirium outcomes. Figure S6. Risk of bias for pressure sore outcomes. Figure S7. Completeness of characteristics reporting. Figure S8. Completeness of outcome reporting. Figure S9. Proportion of patients undergoing non‐operative management. Figure S10. Age vs. proportion receiving non‐operative management. Figure S11. Age vs. proportion receiving non‐operative management by World Bank Region. Figure S12. Median date of cohort vs. proportion receiving non‐operative management. Figure S13. Median date of cohort vs. proportion receiving non‐operative management by World Bank Region. Figure S14. Dementia vs. proportion receiving non‐operative management. Figure S15. Non‐operative proportion vs. 30‐day mortality. Figure S16. Non‐operative proportion vs. 1‐year mortality. Figure S17. Relative risk of delirium, pressure sores and complications with non‐operative management. Figure S18. Risk ratio for female vs. non‐operative management. Figure S19. Mean difference in age vs. non‐operative management. Figure S20. Risk ratio for ASA physical status vs. non‐operative management. Figure S21. Risk ratio for fracture type vs. non‐operative management. Figure S22. Risk ratio for admission from own home vs. non‐operative management. Figure S23. Mean difference in duration of hospital stay vs. non‐operative management. Figure S24. Risk ratio for delirium vs. non‐operative management. Figure S25. Risk ratio for pressure sores vs. non‐operative management. Figure S26. Risk ratio for complications vs. non‐operative management [file ANAE-80-1397-s001.docx]

## Geographical distributions

### Figure S1: Map of distribution of cohorts with characteristics data by country.

#### A) Distribution of number of included cohorts with characteristics data by country.

#### B) Distribution of number of included patients with characteristics data by country.

### Figure S2: Map of distribution of cohorts with outcome data by country.

#### A): Distribution of number of included cohorts with outcome data by country.

#### B): Distribution of number of included patients with outcome data by country.

## Risk of bias

Administrative: cohorts derived from prospectively gathered routine administrative datasets; registries: cohorts derived from prospectively gathered regional / national registries; matched: cohorts with control (operative) populations matched on key factors (e.g. age, comorbidities)

### Figure S3: Risk of bias tables for cohort characteristics

**Risk of bias tables for cohort characteristics**

Rep non-op/op: representativeness of cohort non-operative and operative

Ascertain: ascertainment of exposure

Outcome: Outcome not present before exposure

Comparability: comparability of cohorts

**Risk of bias tables for main outcomes**

Assessment: appropriate assessment of outcome

Duration: duration of follow-up

Adequacy: adequacy of follow-up (missingness)

### Figure S4 Risk of bias for mortality outcomes.

The assessment was based on mortality at any time point in the cohorts.

### Figure S5: Risk of bias for delirium outcomes.

### Figure S6: Risk of bias for pressure sore outcomes.

## Completeness of data reporting.

### Figure S7: Completeness of characteristics reporting

Multicentre: cohorts from more than one centre but not administrative or registry data; administrative: cohorts derived from prospectively gathered routine administrative datasets; registries: cohorts derived from prospectively gathered regional / national registries; matched: cohorts with control (operative) populations matched on key factors (e.g. age, comorbidities)

 

Other: single-centre reports

### Figure S8: Completeness of outcome reporting

Multicentre: cohorts from more than one centre but not administrative or registry data; administrative: cohorts derived from prospectively gathered routine administrative datasets; registries: cohorts derived from prospectively gathered regional / national registries; matched: cohorts with control (operative) populations matched on key factors (e.g. age, comorbidities)

Other: single-centre reports.

## Variation of non-operative proportions

Forest plots are presented grouped by World Bank subgroups and with clustering by country.

### Figure S9: Proportion of patients undergoing non-operative management

## Relationship between cohort characteristics and proportions receiving non-operative management.

### Figure S10: Age vs proportion receiving non-operative management

Multicentre: cohorts from more than one centre but not administrative or registry data; administrative: cohorts derived from prospectively gathered routine administrative datasets; registries: cohorts derived from prospectively gathered regional / national registries; matched: cohorts with control (operative) populations matched on key factors (e.g. age, comorbidities); other: single-centre reports.

### Figure S11: Age vs proportion receiving non-operative management by World Bank Region

### Figure S12: Median date of cohort vs proportion receiving non-operative management

Relationship between median date of cohort and proportion receiving non-operative management. Multicentre: cohorts from more than one centre but not administrative or registry data; administrative: cohorts derived from prospectively gathered routine administrative datasets; registries: cohorts derived from prospectively gathered regional / national registries; matched: cohorts with control (operative) populations matched on key factors (e.g. age, comorbidities); other: single-centre reports.

### Figure S13: Median date of cohort vs proportion receiving non-operative management by World Bank Region

### Figure S14: Dementia vs proportion receiving non-operative management.

Multicentre: cohorts from more than one centre but not administrative or registry data; administrative: cohorts derived from prospectively gathered routine administrative datasets.

## Relationship between proportions receiving non-operative management and mortality

### Figure S15: Non-operative proportion vs. 30-day mortality

Administrative: cohorts derived from prospectively gathered routine administrative datasets; registries: cohorts derived from prospectively gathered regional / national registries; other: single-centre reports.

### Figure S16: Non-operative proportion vs. 1-year mortality

Multicentre: cohorts from more than one centre but not administrative or registry data; administrative: cohorts derived from prospectively gathered routine administrative datasets; registries: cohorts derived from prospectively gathered regional / national registries; matched: cohorts with control (operative) populations matched on key factors (e.g. age, comorbidities); other: single-centre reports.

## Relative risks of outcomes

### Figure S17: Relative risk of delirium, pressure sores and complications with non-operative management

Unselected: cohorts which include all patients presenting with hip fracture; high risk: cohorts where the population has been stratified by one or more risk factors for expected worse outcome (e.g. greater age, presence of dementia); matched: cohorts with control (operative) populations matched on key factors (e.g. age, comorbidities)

## Supplementary forest plots

### Figure S18: Risk ratio for female vs. non-operative management

### Figure S19: Mean difference in age vs. non-operative management

### Figure S20: Risk ratio for ASA physical status vs. non-operative management

### Figure S21: Risk ratio for fracture type vs non-operative management

IC – intracapsular

### Figure S22: Risk ratio for admission from own home vs. non-operative management

### Figure S23: Mean difference in duration of hospital stay vs. non-operative management

###

### Figure S24: Risk ratio for delirium vs. non-operative management

### Figure S25: Risk ratio for pressure sores vs. non-operative management

### Figure S26: Risk ratio for complications vs. non-operative management
