# Supplementary material for: Epidemiology, patient outcome and complications after non‐operative management of hip fracture: a systematic review
Source: Anaesthesia. 2025 Aug 25;80(11):1397–413. doi: 10.1111/anae.16732 (PMC12519934; doi:10.1111/anae.16732)
Supplement: Supplementary file 4 — Table S1. Country cohort data. Table S2. Study characteristics. Table S3. Non‐operative proportions reported by study. Table S4. Overall characteristics of included patients. Table S5. Age: summary of age of patients. Table S6. Sex: proportion of female patients. Table S7. ASA physical status: proportion of ASA physical status three to five patients. Table S8. Comorbidity: summary statistics for reported Charlson Comorbidity Index. Table S9. Proportion of patients with dementia: proportion of patients with dementia. Table S10. Proportion of patients with cancer. Table S11. Proportion of patients admitted from home. Table S12. Ambulatory status on admission: proportion of patients independently mobile. Table S13. Activities of daily living: proportion of patients independent in ADLs. Table S14. Ethnicity: proportion with majority ethnicity for the relevant country by study. Table S15. Fracture type: proportion of patients with intracapsular fracture. Table S16. In‐hospital mortality incidence. Table S17. 7‐day mortality incidence. Table S18. 30‐day mortality incidence. Table S19. 90‐day mortality incidence. Table S20. 180‐day mortality incidence. Table S21. 1‐year mortality incidence. Table S22. 2‐year mortality incidence. Table S23. 3‐year mortality incidence. Table S24. 5‐year mortality incidence. Table S25. Duration of hospital stay. Table S26. Discharge destination: proportion of patients returning to own home or nursing home. Table S27. Ambulatory status at discharge. Table S28. Delirium: proportion of patients with delirium. Table S29. Sensitivity analyses for proportions of patients receiving non‐operative management. Table S30. Sensitivity analyses for patient characteristics. Table S31. Sensitivity analyses for outcomes (mortality, length of hospital stay, ambulatory status at discharge, delirium and complications). [file ANAE-80-1397-s003.docx]

### **Study data tables**

### **Table S1:** Country cohort data. Data supporting figure 1 and figure S1 and S2

| **Country / region** | **Number of characteristic cohorts** | **Total number of characteristic patients** | **Number of outcome cohorts** | **Total number of outcome patients** |
| --- | --- | --- | --- | --- |
| Albania | 1 | 2180 | NA | NA |
| Australia | 10 | 117493 | 2 | 7464 |
| Bosnia and Herzegovina | 1 | 66 | 1 | 66 |
| Brazil | 3 | 69121 | 1 | 67845 |
| Brunei | 1 | 31 | 1 | NA |
| Canada | 3 | 162251 | 2 | 79284 |
| Chile | 2 | 34215 | 2 | 34215 |
| China | 11 | 316859 | 3 | 305400 |
| Colombia | 1 | 70 | NA | NA |
| Estonia | 2 | 11571 | 1 | 11210 |
| Finland | 1 | 37459 | NA | NA |
| Germany | 1 | 636 | NA | NA |
| Greece | 1 | 979 | 1 | 979 |
| Hong Kong | 2 | 3470 | 1 | 2914 |
| India | 3 | 1364 | NA | NA |
| Ireland | 1 | 27119 | NA | NA |
| Israel | 6 | 68438 | 4 | 44344 |
| Italy | 3 | 1569048 | 2 | 78906 |
| Japan | 13 | 491896 | 5 | 86184 |
| Kazakhstan | 2 | 610 | 1 | 344 |
| Lithuania | 2 | 505 | 2 | 505 |
| Malaysia | 1 | 80 | NA | NA |
| Malta | 1 | 454 | 1 | 454 |
| Mexico | 1 | 1042 | 1 | 1042 |
| Netherlands | 6 | 184340 | 2 | 1451 |
| New Zealand | 4 | 10278 | 1 | 144 |
| Pakistan | 1 | 138 | 1 | 138 |
| Peru | 1 | 140 | 1 | 140 |
| Romania | 3 | 3821 | 2 | 2876 |
| Russia | 1 | 45491 | 1 | 45491 |
| Saudi Arabia | 1 | 802 | NA | NA |
| Senegal | 1 | 66 | NA | NA |
| Singapore | 15 | 12754 | 12 | 9335 |
| Slovenia | 2 | 1190 | 2 | 1190 |
| South Africa | 2 | 2196 | 1 | 200 |
| South Korea | 11 | 32358 | 7 | 4804 |
| Spain | 6 | 122503 | 2 | 30883 |
| Sri Lanka | 3 | 606 | 1 | 180 |
| Sweden | 1 | 50472 | NA | NA |
| Taiwan | 4 | 142730 | 2 | 7851 |
| Thailand | 5 | 3349 | 4 | 3047 |
| Turkey | 2 | 830 | 2 | 830 |
| United Kingdom | 16 | 805641 | 11 | 62152 |
| United States of America | 14 | 5628735 | 8 | 472418 |

### **Table S2:** Study characteristics

| **Cohort** | **Country** | **Region** | **Cohort date (centre [range])** | **Additional cohort criteria** | **Reasons for non-operative management** |
| --- | --- | --- | --- | --- | --- |
| Australia (VOTOR) | Australia | E Asia and Pacific | 2013 [2009-2016] |  | Not stated |
| Australia & New Zealand (ANZHFR) | Australia | E Asia and Pacific | 2021 [2021-2021] |  | Not indicated; palliation; other |
| Australia & New Zealand (ANZHFR) | New Zealand | E Asia and Pacific | 2021 [2021-2021] |  | Not indicated; palliation; other |
| Australia & New Zealand (ANZHFR) | Australia | E Asia and Pacific | 2022 [2022-2022] |  | Not indicated; palliation; other |
| Australia & New Zealand (ANZHFR) | New Zealand | E Asia and Pacific | 2022 [2022-2022] |  | Not indicated; palliation; other |
| Australia & New Zealand (ANZHFR) | Australia | E Asia and Pacific | 2023 [2023-2023] |  | Not indicated; palliation; other |
| Australia & New Zealand (ANZHFR) | New Zealand | E Asia and Pacific | 2023 [2023-2023] |  | Not indicated; palliation; other |
| England, Wales & NI (NHFD) | England | Europe and Central Asia | 2011 [-] |  | Not stated |
| England, Wales & NI (NHFD) | England | Europe and Central Asia | 2012 [-] |  | Not stated |
| England, Wales & NI (NHFD) | England | Europe and Central Asia | 2013 [-] |  | Not stated |
| England, Wales & NI (NHFD) | England | Europe and Central Asia | 2014 [-] |  | Not stated |
| England, Wales & NI (NHFD) | England | Europe and Central Asia | 2015 [-] |  | Not stated |
| England, Wales & NI (NHFD) | England | Europe and Central Asia | 2016 [-] |  | Not stated |
| England, Wales & NI (NHFD) | England | Europe and Central Asia | 2017 [-] |  | Not stated |
| England, Wales & NI (NHFD) | England | Europe and Central Asia | 2018 [-] |  | Not stated |
| England, Wales & NI (NHFD) | England | Europe and Central Asia | 2019 [-] |  | Not stated |
| England, Wales & NI (NHFD) | England | Europe and Central Asia | 2020 [-] |  | Not stated |
| England, Wales & NI (NHFD) | England | Europe and Central Asia | 2021 [-] |  | Not stated |
| England, Wales & NI (NHFD) | England | Europe and Central Asia | 2022 [-] |  | Not stated |
| England, Wales & NI (NHFD) | England | Europe and Central Asia | 2023 [2023-2023] |  | Died; not indicated; surgery not possible |
| England, Wales & NI (NHFD) | NI | Europe and Central Asia | 2011 [-] |  | Not stated |
| England, Wales & NI (NHFD) | NI | Europe and Central Asia | 2012 [-] |  | Not stated |
| England, Wales & NI (NHFD) | NI | Europe and Central Asia | 2013 [-] |  | Not stated |
| England, Wales & NI (NHFD) | NI | Europe and Central Asia | 2014 [-] |  | Not stated |
| England, Wales & NI (NHFD) | NI | Europe and Central Asia | 2015 [-] |  | Not stated |
| England, Wales & NI (NHFD) | NI | Europe and Central Asia | 2016 [-] |  | Not stated |
| England, Wales & NI (NHFD) | NI | Europe and Central Asia | 2017 [-] |  | Not stated |
| England, Wales & NI (NHFD) | NI | Europe and Central Asia | 2018 [-] |  | Not stated |
| England, Wales & NI (NHFD) | NI | Europe and Central Asia | 2019 [-] |  | Not stated |
| England, Wales & NI (NHFD) | NI | Europe and Central Asia | 2020 [-] |  | Not stated |
| England, Wales & NI (NHFD) | NI | Europe and Central Asia | 2021 [-] |  | Not stated |
| England, Wales & NI (NHFD) | NI | Europe and Central Asia | 2022 [-] |  | Not stated |
| England, Wales & NI (NHFD) | NI | Europe and Central Asia | 2023 [2023-2023] |  | Died; not indicated; surgery not possible |
| England, Wales & NI (NHFD) | Wales | Europe and Central Asia | 2011 [-] |  | Not stated |
| England, Wales & NI (NHFD) | Wales | Europe and Central Asia | 2012 [-] |  | Not stated |
| England, Wales & NI (NHFD) | Wales | Europe and Central Asia | 2013 [-] |  | Not stated |
| England, Wales & NI (NHFD) | Wales | Europe and Central Asia | 2014 [-] |  | Not stated |
| England, Wales & NI (NHFD) | Wales | Europe and Central Asia | 2015 [-] |  | Not stated |
| England, Wales & NI (NHFD) | Wales | Europe and Central Asia | 2016 [-] |  | Not stated |
| England, Wales & NI (NHFD) | Wales | Europe and Central Asia | 2017 [-] |  | Not stated |
| England, Wales & NI (NHFD) | Wales | Europe and Central Asia | 2018 [-] |  | Not stated |
| England, Wales & NI (NHFD) | Wales | Europe and Central Asia | 2019 [-] |  | Not stated |
| England, Wales & NI (NHFD) | Wales | Europe and Central Asia | 2020 [-] |  | Not stated |
| England, Wales & NI (NHFD) | Wales | Europe and Central Asia | 2021 [-] |  | Not stated |
| England, Wales & NI (NHFD) | Wales | Europe and Central Asia | 2022 [-] |  | Not stated |
| England, Wales & NI (NHFD) | Wales | Europe and Central Asia | 2023 [2023-2023] |  | Died; not indicated; surgery not possible |
| Finland (PERFECT) | Finland | Europe and Central Asia | 1999 [-] | Home dwelling | Not stated |
| Finland (PERFECT) | Finland | Europe and Central Asia | 2000 [-] | Home dwelling | Not stated |
| Finland (PERFECT) | Finland | Europe and Central Asia | 2001 [-] | Home dwelling | Not stated |
| Finland (PERFECT) | Finland | Europe and Central Asia | 2002 [-] | Home dwelling | Not stated |
| Finland (PERFECT) | Finland | Europe and Central Asia | 2003 [-] | Home dwelling | Not stated |
| Finland (PERFECT) | Finland | Europe and Central Asia | 2004 [-] | Home dwelling | Not stated |
| Finland (PERFECT) | Finland | Europe and Central Asia | 2005 [-] | Home dwelling | Not stated |
| Finland (PERFECT) | Finland | Europe and Central Asia | 2006 [-] | Home dwelling | Not stated |
| Finland (PERFECT) | Finland | Europe and Central Asia | 2007 [-] | Home dwelling | Not stated |
| Greece (GHFR) | Greece | Europe and Central Asia | 2023 [2022-2023] |  | Not stated |
| Hong Kong (FFR) | Hong Kong | E Asia and Pacific | 2012 [2012-2012] |  | Not stated |
| Hong Kong (FFR) | Hong Kong | E Asia and Pacific | 2012 [2012-2012] |  | Not stated |
| Ireland (IHFD) | Ireland | Europe and Central Asia | 2013 [2012-2013] |  | Not stated |
| Ireland (IHFD) | Ireland | Europe and Central Asia | 2015 [2015-2015] |  | Not stated |
| Ireland (IHFD) | Ireland | Europe and Central Asia | 2018 [2016-2020] |  | Not stated |
| Ireland (IHFD) | Ireland | Europe and Central Asia | 2021 [2021-2021] |  | Not stated |
| Ireland (IHFD) | Ireland | Europe and Central Asia | 2022 [2022-2022] |  | Not stated |
| Israel (INTR) | Israel | Middle East and N Africa | 2010 [2010-2010] |  | Not stated |
| Israel (INTR) | Israel | Middle East and N Africa | 2011 [2011-2011] |  | Not stated |
| Israel (INTR) | Israel | Middle East and N Africa | 2012 [2012-2012] |  | Not stated |
| Israel (INTR) | Israel | Middle East and N Africa | 2013 [2013-2013] |  | Not stated |
| Israel (INTR) | Israel | Middle East and N Africa | 2014 [2014-2014] |  | Not stated |
| Israel (INTR) | Israel | Middle East and N Africa | 2015 [2015-2015] |  | Not stated |
| Israel (INTR) | Israel | Middle East and N Africa | 2016 [2016-2016] |  | Not stated |
| Israel (INTR) | Israel | Middle East and N Africa | 2017 [2017-2017] |  | Not stated |
| Israel (INTR) | Israel | Middle East and N Africa | 2018 [2018-2018] |  | Not stated |
| Israel (INTR) | Israel | Middle East and N Africa | 2019 [2019-2019] |  | Not stated |
| Johansen | United Kingdom | Europe and Central Asia | 2015 [2015-2015] |  | Not stated |
| Mexico (IMSS) | Mexico | Latin America & the Caribbean | 2022 [2021-2022] |  | Not stated |
| Netherlands (DHFA) | Netherlands | Europe and Central Asia | 2020 [2016-2023] |  | Life expectancy |
| S Korea (Hip Fracture Network) | S Korea | E Asia and Pacific | 2015 [2014-2016] |  | Not stated |
| S Korea (KHFR) | S Korea | E Asia and Pacific | 2018 [2014-2016] | Over 50 | Not stated |
| Spain (RNFC) | Spain | Europe and Central Asia | 2018 [2017-2019] |  | Not stated |
| Spain (RNFC) | Spain | Europe and Central Asia | 2018 [2017-2019] | Aged > 95 | Not stated |
| Spain (RNFC) | Spain | Europe and Central Asia | 2018 [2017-2019] | Aged > 100 | Not stated |
| Spain (RNFC) | Spain | Europe and Central Asia | 2018 [2017-2019] | Aged 77 | Not stated |
| Spain (RNFC) | Spain | Europe and Central Asia | 2018 [2017-2019] | Aged 85 | Not stated |
| Sweden (Rikshoft) | Sweden | Europe and Central Asia | 2018 [2016-2020] |  | Not stated |
| Adler | USA | N America | 2018 [2017-2018] | Medicare, community dwelling with dementia | Not stated |
| Barahona | Chile | Latin America & the Caribbean | 2015 [2012-2017] |  | Not stated |
| Berry | USA | N America | 2011 [2008-2013] | Nursing home; dementia | Not stated |
| Burrack Canada | Canada | N America | 2011 [2011-2011] |  | Functional/ambulatory status; medically unfit |
| Burrack Canada | Canada | N America | 2012 [2012-2012] |  | Functional/ambulatory status; medically unfit |
| Burrack Canada | Canada | N America | 2013 [2013-2013] |  | Functional/ambulatory status; medically unfit |
| Burrack Canada | Canada | N America | 2014 [2014-2014] |  | Functional/ambulatory status; medically unfit |
| Burrack Canada | Canada | N America | 2015 [2015-2015] |  | Functional/ambulatory status; medically unfit |
| Burrack Canada | Canada | N America | 2016 [2016-2016] |  | Functional/ambulatory status; medically unfit |
| Burrack Canada | Canada | N America | 2017 [2017-2017] |  | Functional/ambulatory status; medically unfit |
| Burrack Canada | Canada | N America | 2018 [2018-2018] |  | Functional/ambulatory status; medically unfit |
| Burrack Israel | Israel | Middle East and N Africa | 2011 [2011-2011] |  | Functional/ambulatory status; medically unfit |
| Burrack Israel | Israel | Middle East and N Africa | 2012 [2012-2012] |  | Functional/ambulatory status; medically unfit |
| Burrack Israel | Israel | Middle East and N Africa | 2013 [2013-2013] |  | Functional/ambulatory status; medically unfit |
| Burrack Israel | Israel | Middle East and N Africa | 2014 [2014-2014] |  | Functional/ambulatory status; medically unfit |
| Burrack Israel | Israel | Middle East and N Africa | 2015 [2015-2015] |  | Functional/ambulatory status; medically unfit |
| Burrack Israel | Israel | Middle East and N Africa | 2016 [2016-2016] |  | Functional/ambulatory status; medically unfit |
| Burrack Israel | Israel | Middle East and N Africa | 2017 [2017-2017] |  | Functional/ambulatory status; medically unfit |
| Burrack Israel | Israel | Middle East and N Africa | 2018 [2018-2018] |  | Functional/ambulatory status; medically unfit |
| Burrack Netherlands | Netherlands | Europe and Central Asia | 2013 [2013-2013] |  | Functional/ambulatory status; medically unfit |
| Burrack Netherlands | Netherlands | Europe and Central Asia | 2014 [2014-2014] |  | Functional/ambulatory status; medically unfit |
| Burrack Netherlands | Netherlands | Europe and Central Asia | 2015 [2015-2015] |  | Functional/ambulatory status; medically unfit |
| Burrack Netherlands | Netherlands | Europe and Central Asia | 2016 [2016-2016] |  | Functional/ambulatory status; medically unfit |
| Burrack Netherlands | Netherlands | Europe and Central Asia | 2017 [2017-2017] |  | Functional/ambulatory status; medically unfit |
| Burrack Netherlands | Netherlands | Europe and Central Asia | 2018 [2018-2018] |  | Functional/ambulatory status; medically unfit |
| Burrack Taiwan | Taiwan | E Asia and Pacific | 2011 [2011-2011] |  | Functional/ambulatory status; medically unfit |
| Burrack Taiwan | Taiwan | E Asia and Pacific | 2012 [2012-2012] |  | Functional/ambulatory status; medically unfit |
| Burrack Taiwan | Taiwan | E Asia and Pacific | 2013 [2013-2013] |  | Functional/ambulatory status; medically unfit |
| Burrack Taiwan | Taiwan | E Asia and Pacific | 2014 [2014-2014] |  | Functional/ambulatory status; medically unfit |
| Burrack Taiwan | Taiwan | E Asia and Pacific | 2015 [2015-2015] |  | Functional/ambulatory status; medically unfit |
| Burrack Taiwan | Taiwan | E Asia and Pacific | 2016 [2016-2016] |  | Functional/ambulatory status; medically unfit |
| Burrack Taiwan | Taiwan | E Asia and Pacific | 2017 [2017-2017] |  | Functional/ambulatory status; medically unfit |
| Burrack Taiwan | Taiwan | E Asia and Pacific | 2018 [2018-2018] |  | Functional/ambulatory status; medically unfit |
| Burrack USA | USA | N America | 2011 [2011-2011] |  | Functional/ambulatory status; medically unfit |
| Burrack USA | USA | N America | 2012 [2012-2012] |  | Functional/ambulatory status; medically unfit |
| Burrack USA | USA | N America | 2013 [2013-2013] |  | Functional/ambulatory status; medically unfit |
| Burrack USA | USA | N America | 2014 [2014-2014] |  | Functional/ambulatory status; medically unfit |
| Burrack USA | USA | N America | 2015 [2015-2015] |  | Functional/ambulatory status; medically unfit |
| Burrack USA | USA | N America | 2016 [2016-2016] |  | Functional/ambulatory status; medically unfit |
| Burrack USA | USA | N America | 2017 [2017-2017] |  | Functional/ambulatory status; medically unfit |
| Burrack USA | USA | N America | 2018 [2018-2018] |  | Functional/ambulatory status; medically unfit |
| Cancio | Spain | Europe and Central Asia | 2014 [2012-2015] |  | Not stated |
| Castronuovo | Italy | Europe and Central Asia | 2006 [2006-2006] |  | Not stated |
| Cichos | USA | N America | 2009 [2004-2014] |  | Not stated |
| Cram | Canada | N America | 1997 [1995-1999] |  | Not stated |
| Cram | Canada | N America | 2002 [2000-2004] |  | Not stated |
| Cram | Canada | N America | 2007 [2005-2009] |  | Not stated |
| Cram | Canada | N America | 2012 [2010-2014] |  | Not stated |
| Dash | India | S Asia | 2013 [2012-2014] |  | Not stated |
| De Virgilio-Salgado | USA | N America | 2012 [2008-2015] |  | Not stated |
| Fanuele | USA | N America | 2001 [1999-2003] | Medicare | Not stated |
| Hagino | Japan | E Asia and Pacific | 1997 [1997-1997] |  | Patient choice; family choice; severe cognitive impairment; comorbidities; medically unfit |
| Hagino | Japan | E Asia and Pacific | 1997 [1997-1997] |  | Patient choice; family choice; severe cognitive impairment; comorbidities; medically unfit |
| Ireland | Australia | E Asia and Pacific | 2009 [2008-2009] | Veterans affairs | Not stated |
| Jain | Canada | N America | 1995 [1992-1998] |  | Not stated |
| Jang | S Korea | E Asia and Pacific | 2012 [2004-2019] |  | Patient choice; medically unfit |
| Li | China | E Asia and Pacific | 2015 [2013-2017] |  | Not stated |
| Librero | Spain | Europe and Central Asia | 2004 [2002-2005] |  | Not stated |
| Lo | Taiwan | E Asia and Pacific | 2005 [2001-2010] |  | Not stated |
| Longo | Italy | Europe and Central Asia | 2008 [2001-2016] |  | Not stated |
| Miller | USA | N America | 1998 [1997-1999] | Basi-cervical excluded | Not stated |
| Miller | USA | N America | 2001 [2000-2002] | Basi-cervical excluded | Not stated |
| Miller | USA | N America | 2004 [2003-2005] | Basi-cervical excluded | Not stated |
| Miller | USA | N America | 2007 [2006-2008] | Basi-cervical excluded | Not stated |
| Miller | USA | N America | 1992 [1991-1993] | Basi-cervical excluded | Not stated |
| Miller | USA | N America | 1995 [1994-1996] | Basi-cervical excluded | Not stated |
| Miller | USA | N America | 1998 [1997-1999] | Basi-cervical excluded | Not stated |
| Miller | USA | N America | 2001 [2000-2002] | Basi-cervical excluded | Not stated |
| Mitchell | USA | N America | 2004 [2003-2005] | Basi-cervical excluded | Not stated |
| Mitchell | USA | N America | 2007 [2006-2008] | Basi-cervical excluded | Not stated |
| Neuman | USA | N America | 2004 [2002-2006] | Medicare | Not stated |
| Neuman | USA | N America | 2007 [2005-2009] | Nursing home | Not stated |
| Nikkel | USA | N America | 2006 [2000-2011] | In hospital deaths excluded | Not stated |
| Novack | Israel | Middle East and N Africa | 2003 [2001-2005] |  | Not stated |
| Prommik All | Estonia | Europe and Central Asia | 2013 [2009-2017] |  | Surgical/anaesthetic risk; comorbidities; functional/ambulatory status; late presentation; patient choice |
| Rowe | S Korea | E Asia and Pacific | 2001 [2001-2001] |  | Not stated |
| Shoda | Japan | E Asia and Pacific | 2008 [2007-2009] |  | Not stated |
| Stolnicki | Brazil | Latin America & the Caribbean | 2013 [2008-2017] |  | Not stated |
| Stolnicki | Brazil | Latin America & the Caribbean | 2013 [2008-2017] |  | Not stated |
| Vigano | Italy | Europe and Central Asia | 2014 [2011-2016] |  | Not stated |
| Yang | USA | N America | 2005 [2012-2017] | Alzheimer's disease | Not stated |
| Barahona | Chile | Latin America & the Caribbean | 2015 [2012-2018] |  | Not stated |
| Barrett-Lee | England | Europe and Central Asia | 2016 [2014-2019] | Aged > 100 years | Not stated |
| Bugeja | Malta | Europe and Central Asia | 2016 [2015-2016] |  | Not stated |
| Chariyalertsak | Thailand | E Asia and Pacific | 1998 [1997-1998] |  | Not stated |
| Choi | S Korea | E Asia and Pacific | 2006 [2002-2009] |  | Not stated |
| Creeper | Australia | E Asia and Pacific | 2017 [2017-2017] |  | Not stated |
| Daraphongsataporn | Thailand | E Asia and Pacific | 2016 [2014-2018] |  | Not stated |
| Dela | South Africa | Sub-saharan Africa | 2018 [2017-2018] |  | Not stated |
| Dhibar | India | S Asia | 2012 [2011-2012] |  | Not stated |
| Issayeva | Kazakhstan | Europe and Central Asia | 2016 [2015-2016] |  | Not stated |
| Jang B-W | S Korea | E Asia and Pacific | 2009 [2004-2014] | Aged > 100 years | Not stated |
| Loggers All | Netherlands | Europe and Central Asia | 2019 [2018-2020] | Frail instiutionalised | Patient choice; family choice |
| Mafirakureva | South Africa | Sub-saharan Africa | 2011 [2010-2011] |  | Comorbidities |
| McNamara | England | Europe and Central Asia | 1996 [-] | Palliative care | Medically unfit |
| Shubnyakov | Russia | Europe and Central Asia | 2010 [-] |  | Not stated |
| Shubnyakov | Russia | Europe and Central Asia | 2011 [-] |  | Not stated |
| Shubnyakov | Russia | Europe and Central Asia | 2012 [-] |  | Not stated |
| Shubnyakov | Russia | Europe and Central Asia | 2013 [-] |  | Not stated |
| Shubnyakov | Russia | Europe and Central Asia | 2014 [-] |  | Not stated |
| Shubnyakov | Russia | Europe and Central Asia | 2015 [-] |  | Not stated |
| Shubnyakov | Russia | Europe and Central Asia | 2016 [-] |  | Not stated |
| Shubnyakov | Russia | Europe and Central Asia | 2017 [-] |  | Not stated |
| Shubnyakov | Russia | Europe and Central Asia | 2018 [-] |  | Not stated |
| Shubnyakov | Russia | Europe and Central Asia | 2019 [-] |  | Not stated |
| Tamulaitiene | Lithuania | Europe and Central Asia | 2010 [2010-2010] |  | Not stated |
| Zhang | China | E Asia and Pacific | 2019 [2018-2020] |  | Not stated |
| Zhang | China | E Asia and Pacific | 2019 [2018-2020] |  | Not stated |
| Zielinski | Netherlands | Europe and Central Asia | 2009 [2008-2009] |  | Not stated |
| Abeygunasekara | Sri Lanka | S Asia | 2018 [2017-2020] |  | Advanced age; medically unfit, comorbidities, patient choice, family choice |
| Ahmad | Pakistan | S Asia | 2017 [2015-2018] |  | Not stated |
| Alsheikh | Saudi Arabia | Middle East and N Africa | 2013 [2008-2018] |  | Not stated |
| Amphansap | Thailand | E Asia and Pacific | 2013 [2013-2013] |  | Medically unfit; patient choice; family choice |
| Amrayev | Kazakhstan | Europe and Central Asia | 2014 [2013-2014] |  | Patient choice |
| Atthakomol | Thailand | E Asia and Pacific | 2016 [2014-2018] |  | Not stated |
| Bell | Australia | E Asia and Pacific | 2011 [2010-2011] | Hip fracture | Not stated |
| Beloosesky | Israel | Middle East and N Africa | 1997 [1996-1998] |  | Medically unfit; patient choice; comorbidities; functional/ambulatory status |
| Beringer | NI | Europe and Central Asia | 2000 [1999-2001] |  | Not stated |
| Chaysri | Thailand | E Asia and Pacific | 2007 [2006-2007] |  | Not stated |
| Chen | China | E Asia and Pacific | 2013 [2010-2015] |  | Not stated |
| Chen | China | E Asia and Pacific | 2018 [2017-2018] |  | Not stated |
| Chia | Australia | E Asia and Pacific | 2010 [2009-2010] |  | Not stated |
| Chlebeck All | USA | N America | 2008 [2004-2012] |  | Not stated |
| Coventry | Australia | E Asia and Pacific | 2013 [2012-2014] |  | Not stated |
| Dawe | England | Europe and Central Asia | 2010 [2010-2010] |  | Not stated |
| de Miranda | Brazil | Latin America & the Caribbean | 2017 [2014-2019] |  | Medically unfit; surgical/anaesthetic risk; comorbidities |
| Declarador | Singapore | E Asia and Pacific | 2012 [2011-2012] |  | Not stated |
| Dedovic | Bosnia and Herzegovina | Europe and Central Asia | 2011 [2011-2012] | Three or more comorbidities | Not stated |
| Dick | England | Europe and Central Asia | 2012 [2008-2015] | Aged > 100 | Not stated |
| Dobre | Romania | Europe and Central Asia | 2018 [2017-2018] |  | Clinician recommendation; patient choice |
| Doshi | Singapore | E Asia and Pacific | 2012 [2011-2012] |  | Comorbidities; patient choice |
| Duni | Albania | Europe and Central Asia | 2009 [2004-2014] |  | Patient choice |
| Faraj | England | Europe and Central Asia | 1999 [1995-2002] |  | Surgical/anaesthetic risk; patient choice |
| Frenkel Rutenberg | Israel | Middle East and N Africa | 2013 [2011-2016] |  | Medically unfit; functional/ambulatory status; patient choice; family choice; late presentation |
| Gheorghevici | Romania | Europe and Central Asia | 2018 [2017-2019] |  | Not stated |
| Gonul | Turkey | Europe and Central Asia | 2021 [2020-2021] |  | Not stated |
| Gregory | England | Europe and Central Asia | 2009 [2009-2009] | Intracapsular | Comorbidities; medically unfit; severe cognitive impairment; patient choice; family choice |
| Guo | China | E Asia and Pacific | 2016 [2013-2018] |  | Not stated |
| Hagino | Japan | E Asia and Pacific | 1997 [1997-1997] | Aged > 90 | Patient choice; family choice; severe cognitive impairment; comorbidities; medically unfit |
| Hagino | Japan | E Asia and Pacific | 1997 [1997-1997] | Aged > 95 | Patient choice; family choice; severe cognitive impairment; comorbidities; medically unfit |
| Hagino | Japan | E Asia and Pacific | 2004 [1997-2010] | Aged 70 | Patient choice; family choice; severe cognitive impairment; comorbidities; medically unfit |
| Hagino | Japan | E Asia and Pacific | 2004 [2001-2008] | Aged 80 | Not stated |
| Hay | England | Europe and Central Asia | 2001 [1989-1995] | Immobile before fracture | Medically unfit |
| Heyes | NI | Europe and Central Asia | 2011 [2010-2012] |  | Surgical/anaesthetic risk;, medically unfit; patient choice; severe cognitive impairment; cancer |
| Heyzer | Singapore | E Asia and Pacific | 2012 [2011-2012] |  | Not stated |
| Heyzer | Singapore | E Asia and Pacific | 2013 [2011-2016] |  | Not stated |
| Holloway | Australia | E Asia and Pacific | 2014 [2014-2014] | Women only; hip fracture group is identifiable | Not stated |
| Hwang | S Korea | E Asia and Pacific | 2005 [2000-2009] |  | Not stated |
| Ishimaru | Japan | E Asia and Pacific | 2005 [2001-2009] |  | Cancer; functional/ambulatory status; severe cognitive impairment; clinician recommendation; surgical/anaesthetic risk |
| Jayasekera | Sri Lanka | S Asia | 2017 [2017-2017] |  | Not stated |
| Jiang | China | E Asia and Pacific | 2010 [2000-2019] |  | Not stated |
| Jimenez-Mola | Spain | Europe and Central Asia | 2014 [2013-2014] |  | Not stated |
| Jimenez-Mola | Spain | Europe and Central Asia | 2014 [2013-2014] | Aged > 95 | Not stated |
| Jimenez-Mola | Spain | Europe and Central Asia | 2014 [2013-2014] | Aged 80 | Not stated |
| Jimenez-Mola | Spain | Europe and Central Asia | 2014 [2013-2014] | Aged 85 | Not stated |
| Jin | China | E Asia and Pacific | 2010 [2008-2012] |  | Not stated |
| Kau | Singapore | E Asia and Pacific | 2009 [2009-2010] |  | Patient choice; medically unfit; comorbidities; functional/ambulatory status |
| Kawaji | Japan | E Asia and Pacific | 1999 [1993-2006] |  | Medically unfit; surgical/anaesthetic risk; comorbidities; family choice; cancer |
| Kent | England | Europe and Central Asia | 2005 [2004-2005] | Aged > 100 | Medically unfit; patient choice |
| Kim | S Korea | E Asia and Pacific | 2012 [2010-2014] |  | Not stated |
| Kimura | Japan | E Asia and Pacific | 2015 [2013-2016] |  | Not stated |
| Kir | Turkey | Europe and Central Asia | 2007 [2004-2010] |  | Not stated |
| Kristan | Slovenia | Europe and Central Asia | 2016 [2016-2016] |  | Surgical/anaesthetic risk; comorbidities; functional/ambulatory status; undisplaced fracture |
| Krusic | Slovenia | Europe and Central Asia | 2015 [2014-2015] |  | Not stated |
| Lee A | Singapore | E Asia and Pacific | 2004 [2004-2004] |  | Died; not stated |
| Lee G | S Korea | E Asia and Pacific | 2012 [2010-2014] |  | Not stated |
| Lee J | S Korea | E Asia and Pacific | 2016 [2013-2019] |  | Medically unfit; comorbidities; patient choice; family choice |
| Lee Y | Singapore | E Asia and Pacific | 2001 [2001-2001] |  | Surgical/anaesthetic risk; comorbidities; patient choice |
| Lekamwasam | Sri Lanka | S Asia | 2015 [2014-2015] |  | Not stated |
| Lim | Hong Kong | E Asia and Pacific | 2011 [2011-2012] |  | Medically unfit; surgical/anaesthetic risk; patient choice |
| Lin | USA | N America | 2018 [2018-2019] |  | Not stated |
| Lin | Singapore | E Asia and Pacific | 2001 [2001-2001] |  | Not stated |
| Liu | China | E Asia and Pacific | 2011 [2007-2016] | Aged > 90 | Not stated |
| Lv | China | E Asia and Pacific | 2006 [2000-2011] |  | Not stated |
| Malhotra | Singapore | E Asia and Pacific | 2011 [2010-2012] | Aged 90-99 | Patient choice; family choice |
| Marshall | Malaysia | E Asia and Pacific | 2018 [2016-2016] |  | Not stated |
| Marya | India | S Asia | 2006 [2001-2004] |  | Functional/ambulatory status; comorbidities |
| Mebouinz | Senegal | Sub-saharan Africa | 2013 [2008-2017] | Aged > 90 | Not stated |
| Morris | New Zealand | E Asia and Pacific | 2011 [2011-2011] |  | Not stated |
| Morris | New Zealand | E Asia and Pacific | 2017 [2017-2017] |  | Not stated |
| Moulton | England | Europe and Central Asia | 2011 [2010-2012] |  | Not stated |
| Murray | Germany | Europe and Central Asia | 2014 [2012-2016] | Terminal malignancy excluded | Not stated |
| Nakamura | Japan | E Asia and Pacific | 2016 [2014-2015] |  | Comorbidities |
| Ooi | Singapore | E Asia and Pacific | 1999 [1998-1999] | Aged > 90 | Medically unfit; clinician recommendation |
| Ovidiu | Romania | Europe and Central Asia | 2011 [2007-2015] | Aged > 90 | Surgical/anaesthetic risk; clinician recommendation |
| Poh | Singapore | E Asia and Pacific | 2010 [2010-2010] |  | Patient choice; family choice |
| Reguant | Spain | Europe and Central Asia | 2009 [2008-2009] |  | Not stated |
| Ribeiro | Brazil | Latin America & the Caribbean | 2009 [2005-2012] |  | Not stated |
| Rondon | Peru | Latin America & the Caribbean | 2017 [2015-2018] |  | Not stated |
| Sanz-Reig | Spain | Europe and Central Asia | 2013 [2011-2014] |  | Not stated |
| Shabat | Israel | Middle East and N Africa | 1996 [1990-2001] | Aged > 100 | Not stated |
| Shen | Taiwan | E Asia and Pacific | 2015 [2011-2019] |  | Not stated |
| Shigemoto | Japan | E Asia and Pacific | 2016 [2014-2018] |  | Not stated |
| Shin All | USA | N America | 2014 [2009-2018] |  | Patient choice; family choice; clinician recommendation |
| Sivakumar | Australia | E Asia and Pacific | 2011 [2010-2011] |  | Not stated |
| Stone | USA | N America | 2003 [2000-2004] |  | Family choice; cancer; severe cognitive impairment; functional/ambulatory status |
| Sura-Amonrattana | Thailand | E Asia and Pacific | 2017 [2016-2018] |  | Not stated |
| Takahashi | Japan | E Asia and Pacific | 2017 [2016-2018] |  | Referral; surgical/anaesthetic risk |
| Takamine | Japan | E Asia and Pacific | 2008 [2007-2008] |  | Comorbidities |
| Tan C | Singapore | E Asia and Pacific | 2016 [2015-2017] |  | Comorbidities; functional/ambulatory status |
| Tan L | Singapore | E Asia and Pacific | 2011 [2011-2011] |  | Medically unfit; patient choice; family choice; clinician recommendation |
| Tan S | Singapore | E Asia and Pacific | 2003 [2000-2005] |  | Not stated |
| Tan W | Singapore | E Asia and Pacific | 2015 [2000-2010] |  | Not stated |
| Tay | Singapore | E Asia and Pacific | 2008 [2008-2008] |  | Medically unfit; surgical/anaesthetic risk |
| Teo (Brunei) | Brunei | E Asia and Pacific | 2014 [2014-2014] |  | Not stated |
| Teo (NZ) | New Zealand | E Asia and Pacific | 2010 [2010-2010] |  | Not stated |
| Tian M | China | E Asia and Pacific | 2010 [2009-2011] |  | Not stated |
| Tian W | China | E Asia and Pacific | 2014 [2013-2016] |  | Not stated |
| Ueoka | Japan | E Asia and Pacific | 2015 [2014-2016] |  | Not stated |
| Valaviciene | Lithuania | Europe and Central Asia | 2008 [2008-2008] |  | Medically unfit; comorbidities; |
| Vallejo-Gonzalez | Colombia | Latin America & the Caribbean | 2018 [2018-2018] |  | Not stated |
| van der Zwaard | Netherlands | Europe and Central Asia | 2014 [2014-2014] |  | Patient choice; severe cognitive impairment; functional/ambulatory status; comorbidities |
| van der Zwaard | Netherlands | Europe and Central Asia | 2015 [2015-2015] |  | Medically unfit; functional/ambulatory status; patient choice; family choice; comorbidities; clinician recommendation |
| Vaseenon | Thailand | E Asia and Pacific | 2001 [1998-2003] |  | Comorbidities |
| Viru | Estonia | Europe and Central Asia | 2017 [2017-2017] |  | Not stated |
| Wang | Taiwan | E Asia and Pacific | 2015 [2010-2020] | Aged > 90 | Not stated |
| Wang Z (b) | China | E Asia and Pacific | 2016 [2014-2018] |  | Not stated |
| Wijnen | Netherlands | Europe and Central Asia | 2015 [2011-2019] | Orthogeriatric ward only | Patient choice; family choice; clinician recommendation |
| Wimalasena | New Zealand | E Asia and Pacific | 2013 [2013-2013] |  | Not stated |
| Yagi | Japan | E Asia and Pacific | 2015 [2013-2018] |  | Not stated |
| Yamauchi | Japan | E Asia and Pacific | 2012 [2010-2013] | COPD | Not stated |
| Yoon All | S Korea | E Asia and Pacific | 2010 [2009-2011] |  | Not stated |

### **Table S3:** Non-operative proportions reported by study

| **Cohort type** | **Cohort** | **Cohort detail** | **Study** | **Number** | **Total** | **Risk** | **Proportion (%)** |
| --- | --- | --- | --- | --- | --- | --- | --- |
| Registry | Australia (VOTOR) |  | Giummarra | 307 | 4912 | Unselected | 6.3% |
| Registry | Australia & New Zealand (ANZHFR) | Australia 2021 | ANZHFR | 280 | 11763 | Unselected | 2.4% |
| Registry | Australia & New Zealand (ANZHFR) | New Zealand 2021 | ANZHFR | 71 | 3074 | Unselected | 2.3% |
| Registry | Australia & New Zealand (ANZHFR) | Australia 2022 | ANZHFR | 253 | 12502 | Unselected | 2.0% |
| Registry | Australia & New Zealand (ANZHFR) | New Zealand 2022 | ANZHFR | 97 | 3276 | Unselected | 3.0% |
| Registry | Australia & New Zealand (ANZHFR) | Australia 2023 | ANZHFR | 306 | 13658 | Unselected | 2.2% |
| Registry | Australia & New Zealand (ANZHFR) | New Zealand 2023 | ANZHFR | 101 | 3512 | Unselected | 2.9% |
| Registry | England, Wales & NI (NHFD) | England 2011 | NHFD | 1305 | 56765 | Unselected | 2.3% |
| Registry | England, Wales & NI (NHFD) | England 2012 | NHFD | 1349 | 59208 | Unselected | 2.3% |
| Registry | England, Wales & NI (NHFD) | England 2013 | NHFD | 1250 | 58978 | Unselected | 2.1% |
| Registry | England, Wales & NI (NHFD) | England 2014 | NHFD | 1288 | 59642 | Unselected | 2.2% |
| Registry | England, Wales & NI (NHFD) | England 2015 | NHFD | 1289 | 59689 | Unselected | 2.2% |
| Registry | England, Wales & NI (NHFD) | England 2016 | NHFD | 1336 | 60736 | Unselected | 2.2% |
| Registry | England, Wales & NI (NHFD) | England 2017 | NHFD | 1301 | 60553 | Unselected | 2.1% |
| Registry | England, Wales & NI (NHFD) | England 2018 | NHFD | 1393 | 61385 | Unselected | 2.3% |
| Registry | England, Wales & NI (NHFD) | England 2019 | NHFD | 1538 | 59626 | Unselected | 2.6% |
| Registry | England, Wales & NI (NHFD) | England 2020 | NHFD | 1393 | 59797 | Unselected | 2.3% |
| Registry | England, Wales & NI (NHFD) | England 2021 | NHFD | 1593 | 65580 | Unselected | 2.4% |
| Registry | England, Wales & NI (NHFD) | England 2022 | NHFD | 1525 | 65743 | Unselected | 2.3% |
| Registry | England, Wales & NI (NHFD) | England 2023 | NHFD | 1770 | 65827 | Unselected | 2.7% |
| Registry | England, Wales & NI (NHFD) | NI 2011 | NHFD | 41 | 1820 | Unselected | 2.3% |
| Registry | England, Wales & NI (NHFD) | NI 2012 | NHFD | 42 | 1911 | Unselected | 2.2% |
| Registry | England, Wales & NI (NHFD) | NI 2013 | NHFD | 40 | 1839 | Unselected | 2.2% |
| Registry | England, Wales & NI (NHFD) | NI 2014 | NHFD | 40 | 1918 | Unselected | 2.1% |
| Registry | England, Wales & NI (NHFD) | NI 2015 | NHFD | 39 | 1903 | Unselected | 2.0% |
| Registry | England, Wales & NI (NHFD) | NI 2016 | NHFD | 45 | 2073 | Unselected | 2.2% |
| Registry | England, Wales & NI (NHFD) | NI 2017 | NHFD | 47 | 2008 | Unselected | 2.3% |
| Registry | England, Wales & NI (NHFD) | NI 2018 | NHFD | 45 | 2168 | Unselected | 2.1% |
| Registry | England, Wales & NI (NHFD) | NI 2019 | NHFD | 48 | 1999 | Unselected | 2.4% |
| Registry | England, Wales & NI (NHFD) | NI 2020 | NHFD | 47 | 2104 | Unselected | 2.2% |
| Registry | England, Wales & NI (NHFD) | NI 2021 | NHFD | 48 | 2262 | Unselected | 2.1% |
| Registry | England, Wales & NI (NHFD) | NI 2022 | NHFD | 55 | 2277 | Unselected | 2.4% |
| Registry | England, Wales & NI (NHFD) | NI 2023 | NHFD | 67 | 2287 | Unselected | 2.9% |
| Registry | England, Wales & NI (NHFD) | Wales 2011 | NHFD | 126 | 3583 | Unselected | 3.5% |
| Registry | England, Wales & NI (NHFD) | Wales 2012 | NHFD | 126 | 3866 | Unselected | 3.3% |
| Registry | England, Wales & NI (NHFD) | Wales 2013 | NHFD | 118 | 3678 | Unselected | 3.2% |
| Registry | England, Wales & NI (NHFD) | Wales 2014 | NHFD | 127 | 3653 | Unselected | 3.5% |
| Registry | England, Wales & NI (NHFD) | Wales 2015 | NHFD | 135 | 3870 | Unselected | 3.5% |
| Registry | England, Wales & NI (NHFD) | Wales 2016 | NHFD | 115 | 3878 | Unselected | 3.0% |
| Registry | England, Wales & NI (NHFD) | Wales 2017 | NHFD | 109 | 3994 | Unselected | 2.7% |
| Registry | England, Wales & NI (NHFD) | Wales 2018 | NHFD | 97 | 4118 | Unselected | 2.4% |
| Registry | England, Wales & NI (NHFD) | Wales 2019 | NHFD | 118 | 4114 | Unselected | 2.9% |
| Registry | England, Wales & NI (NHFD) | Wales 2020 | NHFD | 123 | 4046 | Unselected | 3.0% |
| Registry | England, Wales & NI (NHFD) | Wales 2021 | NHFD | 112 | 4560 | Unselected | 2.5% |
| Registry | England, Wales & NI (NHFD) | Wales 2022 | NHFD | 127 | 4336 | Unselected | 2.9% |
| Registry | England, Wales & NI (NHFD) | Wales 2023 | NHFD | 209 | 4336 | Unselected | 4.8% |
| Registry | Finland (PERFECT) | 1999 | Sund | 249 | 4086 | Not high risk | 6.1% |
| Registry | Finland (PERFECT) | 2000 | Sund | 259 | 4115 | Not high risk | 6.3% |
| Registry | Finland (PERFECT) | 2001 | Sund | 262 | 4237 | Not high risk | 6.2% |
| Registry | Finland (PERFECT) | 2002 | Sund | 256 | 4278 | Not high risk | 6.0% |
| Registry | Finland (PERFECT) | 2003 | Sund | 255 | 4186 | Not high risk | 6.1% |
| Registry | Finland (PERFECT) | 2004 | Sund | 226 | 4052 | Not high risk | 5.6% |
| Registry | Finland (PERFECT) | 2005 | Sund | 277 | 4208 | Not high risk | 6.6% |
| Registry | Finland (PERFECT) | 2006 | Sund | 274 | 4163 | Not high risk | 6.6% |
| Registry | Finland (PERFECT) | 2007 | Sund | 260 | 4134 | Not high risk | 6.3% |
| Registry | Greece (GHFR) |  | Ileopoulos | 41 | 979 | Unselected | 4.2% |
| Registry | Hong Kong (FFR) |  | Chow | 60 | 1050 | Unselected | 5.7% |
| Registry | Hong Kong (FFR) |  | Leung | 140 | 2914 | Unselected | 4.8% |
| Registry | Ireland (IHFD) |  | Ellanti | 9 | 826 | Unselected | 1.1% |
| Registry | Ireland (IHFD) | 2015 | IHFD | 124 | 2962 | Unselected | 4.2% |
| Registry | Ireland (IHFD) | 2018 | IHFD | 665 | 15616 | Unselected | 4.3% |
| Registry | Ireland (IHFD) | 2021 | IHFD | 167 | 3806 | Unselected | 4.4% |
| Registry | Ireland (IHFD) | 2022 | IHFD | 186 | 3909 | Unselected | 4.8% |
| Registry | Israel (INTR) | 2010 | Lotan | 285 | 3480 | Unselected | 8.2% |
| Registry | Israel (INTR) | 2011 | Lotan | 287 | 3498 | Unselected | 8.2% |
| Registry | Israel (INTR) | 2012 | Lotan | 220 | 3489 | Unselected | 6.3% |
| Registry | Israel (INTR) | 2013 | Lotan | 208 | 3673 | Unselected | 5.7% |
| Registry | Israel (INTR) | 2014 | Lotan | 194 | 3836 | Unselected | 5.1% |
| Registry | Israel (INTR) | 2015 | Lotan | 238 | 4255 | Unselected | 5.6% |
| Registry | Israel (INTR) | 2016 | Lotan | 251 | 4278 | Unselected | 5.9% |
| Registry | Israel (INTR) | 2017 | Lotan | 192 | 4009 | Unselected | 4.8% |
| Registry | Israel (INTR) | 2018 | Lotan | 209 | 4053 | Unselected | 5.2% |
| Registry | Israel (INTR) | 2019 | Lotan | 222 | 4270 | Unselected | 5.2% |
| Registry | Johansen |  | Johansen | 1427 | 60796 | Unselected | 2.3% |
| Registry | Mexico (IMSS) |  | Duarte-Flores | 47 | 1042 | Unselected | 4.5% |
| Registry | Netherlands (DHFA) |  | van Bremen | 3065 | 94390 | Unselected | 3.2% |
| Registry | S Korea (Hip Fracture Network) | | Ko | 34 | 1841 | Unselected | 1.8% |
| Registry | S Korea (KHFR) |  | Park | 160 | 4963 | Unselected | 3.2% |
| Registry | Spain (RNFC) | All | Bermejo Boixareu | 669 | 25938 | Unselected | 2.6% |
| Registry | Spain (RNFC) | Age 95 | Bermejo Boixareu | 281 | 8035 | High risk | 3.5% |
| Registry | Spain (RNFC) | Age > 100 | Bermejo Boixareu | 17 | 253 | High risk | 6.7% |
| Registry | Spain (RNFC) | Age 77 | Bermejo Boixareu | 61 | 2888 | Not high risk | 2.1% |
| Registry | Spain (RNFC) | Age 80 | Bermejo Boixareu | 310 | 14762 | Not high risk | 2.1% |
| Registry | Sweden (Rikshoft) |  | Hernefalk | 2624 | 50472 | Unselected | 5.2% |
| Administrative | Adler |  | Adler | 23067 | 56209 | High risk | 41.0% |
| Administrative | Barahona |  | Barahona | 7443 | 30975 | Unselected | 24.0% |
| Administrative | Berry |  | Berry | 468 | 3083 | High risk | 15.2% |
| Administrative | Burrack Canada | 2011 | Burrack* | 532 | 9495 | Unselected | 5.6% |
| Administrative | Burrack Canada | 2012 | Burrack | 521 | 10021 | Unselected | 5.2% |
| Administrative | Burrack Canada | 2013 | Burrack | 573 | 10410 | Unselected | 5.5% |
| Administrative | Burrack Canada | 2014 | Burrack | 603 | 10574 | Unselected | 5.7% |
| Administrative | Burrack Canada | 2015 | Burrack | 645 | 10411 | Unselected | 6.2% |
| Administrative | Burrack Canada | 2016 | Burrack | 708 | 10408 | Unselected | 6.8% |
| Administrative | Burrack Canada | 2017 | Burrack | 745 | 10642 | Unselected | 7.0% |
| Administrative | Burrack Canada | 2018 | Burrack | 748 | 11006 | Unselected | 6.8% |
| Administrative | Burrack Israel | 2011 | Burrack | 355 | 2628 | Unselected | 13.5% |
| Administrative | Burrack Israel | 2012 | Burrack | 299 | 2624 | Unselected | 11.4% |
| Administrative | Burrack Israel | 2013 | Burrack | 291 | 3130 | Unselected | 9.3% |
| Administrative | Burrack Israel | 2014 | Burrack | 280 | 2977 | Unselected | 9.4% |
| Administrative | Burrack Israel | 2015 | Burrack | 243 | 2931 | Unselected | 8.3% |
| Administrative | Burrack Israel | 2016 | Burrack | 235 | 3220 | Unselected | 7.3% |
| Administrative | Burrack Israel | 2017 | Burrack | 172 | 3133 | Unselected | 5.5% |
| Administrative | Burrack Israel | 2018 | Burrack | 201 | 3298 | Unselected | 6.1% |
| Administrative | Burrack Netherlands | 2013 | Burrack | 828 | 12945 | Unselected | 6.4% |
| Administrative | Burrack Netherlands | 2014 | Burrack | 1246 | 14004 | Unselected | 8.9% |
| Administrative | Burrack Netherlands | 2015 | Burrack | 1300 | 14446 | Unselected | 9.0% |
| Administrative | Burrack Netherlands | 2016 | Burrack | 1099 | 14654 | Unselected | 7.5% |
| Administrative | Burrack Netherlands | 2017 | Burrack | 1113 | 14834 | Unselected | 7.5% |
| Administrative | Burrack Netherlands | 2018 | Burrack | 1229 | 15170 | Unselected | 8.1% |
| Administrative | Burrack Taiwan | 2011 | Burrack | 853 | 15792 | Unselected | 5.4% |
| Administrative | Burrack Taiwan | 2012 | Burrack | 780 | 15910 | Unselected | 4.9% |
| Administrative | Burrack Taiwan | 2013 | Burrack | 777 | 16181 | Unselected | 4.8% |
| Administrative | Burrack Taiwan | 2014 | Burrack | 691 | 16457 | Unselected | 4.2% |
| Administrative | Burrack Taiwan | 2015 | Burrack | 627 | 16498 | Unselected | 3.8% |
| Administrative | Burrack Taiwan | 2016 | Burrack | 841 | 17519 | Unselected | 4.8% |
| Administrative | Burrack Taiwan | 2017 | Burrack | 803 | 17467 | Unselected | 4.6% |
| Administrative | Burrack Taiwan | 2018 | Burrack | 824 | 17917 | Unselected | 4.6% |
| Administrative | Burrack USA | 2011 | Burrack | 9374 | 161626 | Unselected | 5.8% |
| Administrative | Burrack USA | 2012 | Burrack | 8821 | 152083 | Unselected | 5.8% |
| Administrative | Burrack USA | 2013 | Burrack | 9130 | 152159 | Unselected | 6.0% |
| Administrative | Burrack USA | 2014 | Burrack | 9265 | 157026 | Unselected | 5.9% |
| Administrative | Burrack USA | 2015 | Burrack | 10234 | 155066 | Unselected | 6.6% |
| Administrative | Burrack USA | 2016 | Burrack | 11151 | 150695 | Unselected | 7.4% |
| Administrative | Burrack USA | 2017 | Burrack | 11226 | 147712 | Unselected | 7.6% |
| Administrative | Burrack USA | 2018 | Burrack | 11036 | 143329 | Unselected | 7.7% |
| Administrative | Cancio |  | Cancio | 1650 | 30552 | Unselected | 5.4% |
| Administrative | Castronuovo |  | Castronuovo | 1117 | 6986 | Unselected | 16.0% |
| Administrative | Cichos |  | Cichos | 66964 | 3348207 | Unselected | 2.0% |
| Administrative | Cram | 1997 | Cram | 327 | 4069 | Unselected | 8.0% |
| Administrative | Cram | 2002 | Cram | 225 | 3500 | Unselected | 6.4% |
| Administrative | Cram | 2007 | Cram | 228 | 4165 | Unselected | 5.5% |
| Administrative | Cram | 2012 | Cram | 200 | 3953 | Unselected | 5.1% |
| Administrative | Dash |  | Dash | 304 | 1016 | Unselected | 29.9% |
| Administrative | De Virgilio-Salgado |  | De Virgilio-Salgado | 109 | 268 | Unselected | 40.7% |
| Administrative | Fanuele |  | Fanuele | 3350 | 140195 | Unselected | 2.4% |
| Administrative | Hagino |  | Hagino | 22740 | 402760 | Unselected | 5.6% |
| Administrative | Hagino |  | Hagino | 43 | 512 | Unselected | 8.4% |
| Administrative | Ireland |  | Ireland | 398 | 2552 | Unselected | 15.6% |
| Administrative | Jain | All | Jain All | 5311 | 50235 | Unselected | 10.6% |
| Administrative | Jang |  | Jang | 1478 | 19915 | Unselected | 7.4% |
| Administrative | Li |  | Li | 123002 | 304279 | Unselected | 40.4% |
| Administrative | Librero |  | Librero | 7273 | 64609 | Unselected | 11.3% |
| Administrative | Lo |  | Lo | 1000 | 7646 | Unselected | 13.1% |
| Administrative | Longo |  | Longo | 303693 | 1490142 | Unselected | 20.4% |
| Administrative | Miller | High volume 1998 | Miller | 3971 | 115264 | Unselected | 3.4% |
| Administrative | Miller | High volume 2001 | Miller | 3626 | 101797 | Unselected | 3.6% |
| Administrative | Miller | High volume 2004 | Miller | 3767 | 98623 | Unselected | 3.8% |
| Administrative | Miller | High volume 2007 | Miller | 3427 | 93216 | Unselected | 3.7% |
| Administrative | Miller | Low volume 1998 | Miller | 908 | 10691 | Unselected | 8.5% |
| Administrative | Miller | Low volume 2001 | Miller | 878 | 10059 | Unselected | 8.7% |
| Administrative | Miller | Low volume 2004 | Miller | 847 | 9041 | Unselected | 9.4% |
| Administrative | Miller | Low volume 2007 | Miller | 828 | 8979 | Unselected | 9.2% |
| Administrative | Mitchell |  | Mitchell | 10307 | 69370 | Unselected | 14.9% |
| Administrative | Mitchell |  | Mitchell | 2303 | 18783 | High risk | 12.3% |
| Administrative | Neuman |  | Neuman | 10283 | 165861 | Unselected | 6.2% |
| Administrative | Neuman |  | Neuman | 7069 | 60111 | High risk | 11.8% |
| Administrative | Nikkel |  | Nikkel | 18950 | 180844 | Unselected | 10.5% |
| Administrative | Novack |  | Novack | 818 | 4633 | Unselected | 17.7% |
| Administrative | Prommik All |  | Prommik All | 766 | 11210 | Unselected | 6.8% |
| Administrative | Rowe |  | Rowe | 142 | 1152 | Unselected | 12.3% |
| Administrative | Shoda |  | Shoda | 13907 | 80800 | Unselected | 17.2% |
| Administrative | Stolnicki | 2008 | Stolnicki | 2653 | 27913 | Unselected | 9.5% |
| Administrative | Stolnicki | 2017 | Stolnicki | 1945 | 39932 | Unselected | 4.9% |
| Administrative | Vigano |  | Vigano | 7624 | 71920 | Unselected | 10.6% |
| Administrative | Yang |  | Yang | 315 | 4157 | High risk | 7.6% |
| Multicentre | Barahona |  | Barahona | 235 | 3240 | Unselected | 7.3% |
| Multicentre | Barrett-Lee |  | Barrett-Lee | 4 | 60 | High risk | 6.7% |
| Multicentre | Bugeja |  | Bugeja | 5 | 454 | Unselected | 1.1% |
| Multicentre | Chariyalertsak |  | Chariyalertsak | 155 | 330 | Unselected | 47.0% |
| Multicentre | Choi |  | Choi | 117 | 1388 | Unselected | 8.4% |
| Multicentre | Creeper |  | Creeper | 14 | 1240 | Unselected | 1.1% |
| Multicentre | Daraphongsataporn |  | Daraphongsataporn | 166 | 1180 | Unselected | 14.1% |
| Multicentre | Dela |  | Dela | 153 | 1996 | Unselected | 7.7% |
| Multicentre | Dhibar |  | Dhibar | 21 | 264 | Unselected | 8.0% |
| Multicentre | Issayeva |  | Issayeva | 66 | 266 | Unselected | 24.8% |
| Multicentre | Jang B-W |  | Jang B-W | 6 | 68 | High risk | 8.8% |
| Multicentre | Loggers All |  | Loggers All | 88 | 172 | High risk | 51.2% |
| Multicentre | Mafirakureva |  | Mafirakureva | 27 | 200 | Unselected | 13.5% |
| Multicentre | McNamara |  | McNamara | 28 | 40 | High risk | 70.0% |
| Multicentre | Shubnyakov | 2010 | Shubnyakov | 979 | 3559 | Unselected | 27.5% |
| Multicentre | Shubnyakov | 2011 | Shubnyakov | 916 | 4163 | Unselected | 22.0% |
| Multicentre | Shubnyakov | 2012 | Shubnyakov | 848 | 5049 | Unselected | 16.8% |
| Multicentre | Shubnyakov | 2013 | Shubnyakov | 951 | 4082 | Unselected | 23.3% |
| Multicentre | Shubnyakov | 2014 | Shubnyakov | 1042 | 4395 | Unselected | 23.7% |
| Multicentre | Shubnyakov | 2015 | Shubnyakov | 1124 | 5325 | Unselected | 21.1% |
| Multicentre | Shubnyakov | 2016 | Shubnyakov | 738 | 4584 | Unselected | 16.1% |
| Multicentre | Shubnyakov | 2017 | Shubnyakov | 945 | 4894 | Unselected | 19.3% |
| Multicentre | Shubnyakov | 2018 | Shubnyakov | 763 | 4595 | Unselected | 16.6% |
| Multicentre | Shubnyakov | 2019 | Shubnyakov | 703 | 4845 | Unselected | 14.5% |
| Multicentre | Tamulaitiene |  | Tamulaitiene | 12 | 441 | Unselected | 2.7% |
| Multicentre | Zhang | Comanagement | Zhang | 19 | 1110 | Unselected | 1.7% |
| Multicentre | Zhang | Usual care | Zhang | 100 | 961 | Unselected | 10.4% |
| Multicentre | Zielinski |  | Zielinski | 22 | 1250 | Unselected | 1.8% |
| Single centre | Abeygunasekara |  | Abeygunasekara | 73 | 180 | Unselected | 40.6% |
| Single centre | Ahmad |  | Ahmad | 4 | 138 | Unselected | 2.9% |
| Single centre | Alsheikh |  | Alsheikh | 57 | 802 | Unselected | 7.1% |
| Single centre | Amphansap |  | Amphansap | 21 | 120 | Unselected | 17.5% |
| Single centre | Amrayev |  | Amrayev | 78 | 344 | Unselected | 22.7% |
| Single centre | Atthakomol |  | Atthakomol | 87 | 775 | Unselected | 11.2% |
| Single centre | Bell |  | Bell | 6 | 328 | Unselected | 1.8% |
| Single centre | Beloosesky |  | Beloosesky | 26 | 153 | Unselected | 17.0% |
| Single centre | Beringer |  | Beringer | 111 | 2834 | Unselected | 3.9% |
| Single centre | Chaysri |  | Chaysri | 72 | 275 | Unselected | 26.2% |
| Single centre | Chen | 2013 | Chen 2019 | 183 | 1539 | Unselected | 11.9% |
| Single centre | Chen | 2018 | Chen 2022 | 42 | 620 | Unselected | 6.8% |
| Single centre | Chia |  | Chia | 2 | 185 | Unselected | 1.1% |
| Single centre | Chlebeck All | All | Chlebeck | 77 | 1113 | Unselected | 6.9% |
| Single centre | Coventry |  | Coventry | 3 | 446 | Unselected | 0.7% |
| Single centre | Dawe |  | Dawe | 12 | 251 | Unselected | 4.8% |
| Single centre | de Miranda |  | de Miranda | 28 | 732 | Unselected | 3.8% |
| Single centre | Declarador |  | Declarador | 108 | 554 | Unselected | 19.5% |
| Single centre | Dedovic |  | Dedovic | 32 | 66 | High risk | 48.5% |
| Single centre | Dick |  | Dick | 2 | 23 | High risk | 8.7% |
| Single centre | Dobre |  | Dobre | 450 | 2742 | Unselected | 16.4% |
| Single centre | Doshi |  | Doshi | 40 | 219 | Unselected | 18.3% |
| Single centre | Duni |  | Duni | 478 | 2180 | Unselected | 21.9% |
| Single centre | Faraj |  | Faraj | 21 | 1420 | Unselected | 1.5% |
| Single centre | Frenkel Rutenberg |  | Frenkel Rutenberg | 94 | 847 | Unselected | 11.1% |
| Single centre | Gheorghevici |  | Gheorghevici | 237 | 945 | Unselected | 25.1% |
| Single centre | Gonul |  | Gonul | 2 | 120 | Unselected | 1.7% |
| Single centre | Gregory |  | Gregory | 22 | 102 | Unselected | 21.6% |
| Single centre | Guo |  | Guo | 133 | 1017 | Unselected | 13.1% |
| Single centre | Hagino | Age 90 | Hagino | 18 | 180 | High risk | 10.0% |
| Single centre | Hagino | Age 95 | Hagino | 3 | 23 | High risk | 13.0% |
| Single centre | Hagino | Age 70 | Hagino | 9 | 55 | Not high risk | 16.4% |
| Single centre | Hagino | Age 80 | Hagino | 17 | 172 | Not high risk | 9.9% |
| Single centre | Hay |  | Hay | 9 | 152 | High risk | 5.9% |
| Single centre | Heyes | All | Heyes | 12 | 384 | Unselected | 3.1% |
| Single centre | Heyzer | 2012 | Heyzer | 109 | 557 | Unselected | 19.6% |
| Single centre | Heyzer | 2016 | Heyzer | 298 | 2500 | Unselected | 11.9% |
| Single centre | Holloway |  | Holloway | 25 | 209 | Unselected | 12.0% |
| Single centre | Hwang |  | Hwang | 116 | 807 | Unselected | 14.4% |
| Single centre | Ishimaru |  | Ishimaru | 20 | 666 | Unselected | 3.0% |
| Single centre | Jayasekera |  | Jayasekera | 21 | 117 | Unselected | 17.9% |
| Single centre | Jiang |  | Jiang | 270 | 3075 | Unselected | 8.8% |
| Single centre | Jimenez-Mola | All | Jimenez-Mola | 35 | 534 | Unselected | 6.6% |
| Single centre | Jimenez-Mola | Age 95 | Jimenez-Mola | 14 | 165 | High risk | 8.5% |
| Single centre | Jimenez-Mola | Age 80 | Jimenez-Mola | 14 | 189 | Not high risk | 7.4% |
| Single centre | Jimenez-Mola | Age 85 | Jimenez-Mola | 7 | 180 | Not high risk | 3.9% |
| Single centre | Jin |  | Jin | 28 | 482 | Unselected | 5.8% |
| Single centre | Kau |  | Kau | 59 | 197 | Unselected | 29.9% |
| Single centre | Kawaji |  | Kawaji | 21 | 229 | Unselected | 9.2% |
| Single centre | Kent |  | Kent | 4 | 12 | High risk | 33.3% |
| Single centre | Kim |  | Kim | 4 | 198 | Unselected | 2.0% |
| Single centre | Kimura |  | Kimura | 1 | 496 | Unselected | 0.2% |
| Single centre | Kir |  | Kir | 28 | 710 | Unselected | 3.9% |
| Single centre | Kristan |  | Kristan | 52 | 695 | Unselected | 7.5% |
| Single centre | Krusic |  | Krusic | 34 | 495 | Unselected | 6.9% |
| Single centre | Lee A |  | Lee A | 3 | 70 | Unselected | 4.3% |
| Single centre | Lee G |  | Lee G | 17 | 489 | Unselected | 3.5% |
| Single centre | Lee J |  | Lee J | 93 | 1090 | Unselected | 8.5% |
| Single centre | Lee Y |  | Lee Y | 7 | 69 | Unselected | 10.1% |
| Single centre | Lekamwasam |  | Lekamwasam | 141 | 309 | Unselected | 45.6% |
| Single centre | Lim |  | Lim | 87 | 556 | Unselected | 15.6% |
| Single centre | Lin |  | Lin | 18 | 294 | Unselected | 6.1% |
| Single centre | Lin |  | Lin | 8 | 68 | Unselected | 11.8% |
| Single centre | Liu |  | Liu | 67 | 104 | High risk | 64.4% |
| Single centre | Lv |  | Lv | 138 | 1598 | Unselected | 8.6% |
| Single centre | Malhotra |  | Malhotra | 23 | 65 | High risk | 35.4% |
| Single centre | Marshall |  | Marshall | 19 | 80 | Unselected | 23.8% |
| Single centre | Marya |  | Marya | 4 | 84 | Unselected | 4.8% |
| Single centre | Mebouinz |  | Mebouinz | 9 | 66 | High risk | 13.6% |
| Single centre | Morris | 2011 | Morris | 2 | 74 | Unselected | 2.7% |
| Single centre | Morris | 2017 | Morris | 3 | 107 | Unselected | 2.8% |
| Single centre | Moulton |  | Moulton | 38 | 642 | Unselected | 5.9% |
| Single centre | Murray |  | Murray | 0 | 636 | Not high risk | 0.0% |
| Single centre | Nakamura |  | Nakamura | 57 | 196 | Unselected | 29.1% |
| Single centre | Ooi |  | Ooi | 38 | 84 | High risk | 45.2% |
| Single centre | Ovidiu | Age 95 | Ovidiu Cohort | 40 | 134 | High risk | 29.9% |
| Single centre | Poh |  | Poh | 52 | 294 | Unselected | 17.7% |
| Single centre | Reguant |  | Reguant | 27 | 539 | Unselected | 5.0% |
| Single centre | Ribeiro |  | Ribeiro | 94 | 544 | Unselected | 17.3% |
| Single centre | Rondon |  | Rondon | 16 | 140 | Unselected | 11.4% |
| Single centre | Sanz-Reig |  | Sanz-Reig | 21 | 331 | Unselected | 6.3% |
| Single centre | Shabat |  | Shabat | 4 | 23 | High risk | 17.4% |
| Single centre | Shen |  | Shen | 20 | 1138 | Unselected | 1.8% |
| Single centre | Shigemoto |  | Shigemoto | 24 | 702 | Unselected | 3.4% |
| Single centre | Shin All |  | Shin All | 171 | 772 | Unselected | 22.2% |
| Single centre | Sivakumar |  | Sivakumar | 6 | 328 | Unselected | 1.8% |
| Single centre | Stone |  | Stone | 5 | 255 | Unselected | 2.0% |
| Single centre | Sura-Amonrattana |  | Sura-Amonrattana | 19 | 302 | Unselected | 6.3% |
| Single centre | Takahashi |  | Takahashi | 34 | 228 | Unselected | 14.9% |
| Single centre | Takamine |  | Takamine | 10 | 113 | Unselected | 8.8% |
| Single centre | Tan C |  | Tan C | 233 | 1014 | Unselected | 23.0% |
| Single centre | Tan L |  | Tan L | 57 | 244 | Unselected | 23.4% |
| Single centre | Tan S |  | Tan S | 727 | 2756 | Unselected | 26.4% |
| Single centre | Tan W |  | Tan W | 554 | 2702 | Unselected | 20.5% |
| Single centre | Tay |  | Tay | 114 | 340 | Unselected | 33.5% |
| Single centre | Teo (Brunei) |  | Teo (Brunei) | 8 | 31 | Unselected | 25.8% |
| Single centre | Teo (NZ) |  | Teo (NZ) | 6 | 144 | Unselected | 4.2% |
| Single centre | Tian M |  | Tian M | 78 | 780 | Unselected | 10.0% |
| Single centre | Tian W |  | Tian W | 137 | 644 | Unselected | 21.3% |
| Single centre | Ueoka |  | Ueoka | 18 | 436 | Unselected | 4.1% |
| Single centre | Valaviciene |  | Valaviciene | 5 | 64 | Unselected | 7.8% |
| Single centre | Vallejo-Gonzalez |  | Vallejo-Gonzalez | 10 | 70 | Unselected | 14.3% |
| Single centre | van der Zwaard | 2014 | van der Zwaard | 5 | 185 | Unselected | 2.7% |
| Single centre | van der Zwaard | 2025 | van der Zwaard | 18 | 197 | Unselected | 9.1% |
| Single centre | Vaseenon |  | Vaseenon | 71 | 367 | Unselected | 19.3% |
| Single centre | Viru |  | Viru | 48 | 361 | Unselected | 13.3% |
| Single centre | Wang | Age 90 | Wang Cohort | 32 | 205 | High risk | 15.6% |
| Single centre | Wang Z (b) |  | Wang Z | 221 | 650 | Unselected | 34.0% |
| Single centre | Wijnen | Frail | Wijnen | 91 | 1279 | High risk | 7.1% |
| Single centre | Wimalasena |  | Wimalasena | 1 | 91 | Unselected | 1.1% |
| Single centre | Yagi |  | Yagi | 16 | 269 | Unselected | 5.9% |
| Single centre | Yamauchi |  | Yamauchi | 839 | 4059 | High risk | 20.7% |
| Single centre | Yoon All |  | Yoon All | 28 | 447 | Unselected | 6.3% |

The Burrack data for England are not included as they overlap completely with the NHFD data.

## **Cohort characteristics**

Summaries are presented as mean (SD); median (LQ,, UQ); median [min, max]; ‘average’ value (mean, median or ‘average’ as reported in original paper); number as appropriate. Where a total is given this represents the total number of patients in the non-operative / operative cohort with data for that characteristic.

### **Table S4:** Overall characteristics of included patients. Data are weighted averages, using random effects and clustered by world bank and country. These summary data are provided purely to provide an overview of the populations. Details are elaborated in the tables below.

| **Characteristic** | **Conservative** | **Operative** | **All** |
| --- | --- | --- | --- |
| Age (years) | 85.0 (83.1 - 86.9) | 83.7 (81.0 - 86.3) | 84.0 (81.4 - 86.6) |
| Sex (Female) | 0.69 (0.67 - 0.71) | 0.72 (0.69 - 0.74) | 0.7 (0.69 - 0.72) |
| ASA (3-5) | 0.75 (0.56 - 0.88) | 0.63 (0.37 - 0.83) | 0.62 (0.38 - 0.81) |
| Comorbidity (mean (SD)) | 3.27 (1.96 - 4.58) | 2.68 (1.43 - 3.93) | 2.79 (1.49 - 4.09) |
| Dementia | 0.34 (0.2 - 0.52) | 0.2 (0.11 - 0.35) | 0.26 (0.16 - 0.4) |
| Cancer | 0.12 (0.05 - 0.27) | 0.08 (0.04 - 0.16) | 0.08 (0.04 - 0.17) |
| Admitted from home | 0.61 (0.39 - 0.8) | 0.7 (0.57 - 0.8) | 0.59 (0.36 - 0.78) |
| Independently mobile | 0.41 (0.25 - 0.59) | 0.61 (0.48 - 0.73) | 0.59 (0.46 - 0.71) |
| Independent of ADLs | 0.47 (0.09 - 0.9) | 0.46 (0.02 - 0.97) | 0.36 (0.01 - 0.96) |
| Ethnicity (majority) | 0.72 (0.42 - 0.9) | 0.76 (0.44 - 0.93) | 0.75 (0.44 - 0.92) |
| Fracture type (intracapsular) | 0.51 (0.43 - 0.6) | 0.44 (0.4 - 0.48) | 0.47 (0.38 - 0.56) |

### **Table S5:** Age: summary of age of patients

| **Study** | **Age total** | **Age non-operative** | **Age operative** |
| --- | --- | --- | --- |
| Abeygunasekara | 76.5 (9.15) | 82 (8.5) | 73.6 (8.0) |
| Amrayev | 71.4 (11.2) | 76 (12) | 70 (11) |
| Chlebeck All | 83.9 (7.9) | 86.7 (5.7) | 83.7 (8.0) |
| Chow | 81.2 (9.0) | 82.5 (10) | 81.1 (8.9) |
| Cram Cohort: 1997 | 80.7 (10.5) | 83.2 (8.6) | 80.5 (10.7) |
| Cram Cohort: 2002 | 80.7 (11.2) | 82.2 (10.4) | 80.6 (11.3) |
| Cram Cohort: 2007 | 81.0 (11.2) | 83.5 (11.1) | 80.9 (11.2) |
| Cram Cohort: 2012 | 80.9 (11.3) | 81.9 (12.2) | 81.3 (11) |
| De Virgilio-Salgado | 82.5 (7.1) | 84.8 (6.9) | 81.0 (7.2) |
| Dick | 101.7 (2.1) | 101 (1.4) | 101.8 (2.2) |
| Dobre | 79 (10) | 79 (10) | 79 (10) |
| Frenkel Rutenberg | 84.9 (7.5) | 85 (8.0) | 84.9 (7.4) |
| Gregory | 84 | 84 | 84 |
| Heyes All | 77 (12) | 86.5 (7.6) | 81.5 (17.5) |
| Kau | 80.03 (9.2) | 82.95 (9.58) | 78.78 (8.97) |
| Kent | 101.3 (0.8) | 100.6 (0.5) | 101.5 (0.9) |
| Lee Y | 79.65 [60, 98] | 81 [73, 92] | 79.5 [60, 98] |
| Liu | 92.3 (3.4) | 92.00 (4.00) | 92.83 (2.66) |
| Loggers All | 91 (85, 93) | 88 (84, 93) | 88 (85, 91) |
| Neuman | 83.4 (7.1) | 84.5 (7.1) | 83.3 (7.1) |
| Novack | 82.3 (7.4) | 82.0 (7.5) | 82.3 (7.4) |
| Prommik All | 79.2 (10.7) | 80.5 (11.4) | 79.1 (10.7) |
| Shabat | 101.8 (2.1) | 101 (1.4) | 101.8 (2.2) |
| Tan C | 79.2 | 81.7 | 78.4 |
| Tay | 80.1 (8.2) | 82.8 (8.3) | 78.8 (8.2) |
| Wijnen Cohort: Frail | 84.2 (6.7) | 87 (6.3) | 84 (6.7) |
| Marshall | 75 (7.8) | 79 (8) | 74 (7.7) |
| Tan L | 80.9 | 85.3 | 79.56 |
| Wang Cohort: Age 90 | 92 (91, 94) | 92 (91, 94) | 92 (91,94) |
| van Bremen | 999 | 86 (79, 91) | 81 (72, 87) |
| Ishimaru | 85.3 (6.6) | 83.7 (6.8) | 85.4 (6.6) |
| Yang | 81 (75, 87) | 80 (73, 86) | 81.5 (75, 87) |
| Berry | 84.2 (7.2) | 85.1 (7.5) | 84.0 (7.1) |
| Adler | 86.4 (7.0) | 86.8 (7.0) | 86.1 (7.0) |
| Doshi | 82.1 | 87 | 81 |
| Declarador | 80.9 (8.5) | 83.7 (8.9) | 80.2 (8.4) |
| Guo | 78.4 (8.4) | 79.5 (8.0) | 78.3 (8.5) |
| Greece (GHFR) | 82.2 (8.5) | 87.0 (7.3) | 82.0 (8.6) |
| Tan S | 79.6 | 81.9 | 78.8 |

### **Table S6:** Sex: proportion of female patients

| **Study label** | **Female conservative** | **Total conservative** | **Female conservative proportion** | **Female operative** | **Total operative** | **Female operative proportion** |
| --- | --- | --- | --- | --- | --- | --- |
| Abeygunasekara | 59 | 73 | 80.8% | 90 | 107 | 84.1% |
| Adler | 17066 | 23067 | 74.0% | 23962 | 33142 | 72.3% |
| Amrayev | 51 | 78 | 65.4% | 171 | 266 | 64.3% |
| Berry | 385 | 468 | 82.3% | 2052 | 2615 | 78.5% |
| Chen M 19 | 120 | 183 | 65.6% | 1016 | 1356 | 74.9% |
| Chia | 2 | 2 | 100.0% | 130 | 183 | 71.0% |
| Chlebeck All | 49 | 77 | 63.6% | 734 | 1036 | 70.8% |
| Chow | 34 | 60 | 56.7% | 702 | 990 | 70.9% |
| Cram Cohort: 1997 | 224 | 327 | 68.5% | 2738 | 3742 | 73.2% |
| Cram Cohort: 2002 | 170 | 225 | 75.6% | 2406 | 3275 | 73.5% |
| Cram Cohort: 2007 | 146 | 228 | 64.0% | 2789 | 3937 | 70.8% |
| Cram Cohort: 2012 | 125 | 200 | 62.5% | 2731 | 3753 | 72.8% |
| De Virgilio-Salgado | 0 | 109 | 0.0% | 0 | 159 | 0.0% |
| Dedovic | 24 | 32 | 75.0% | 23 | 34 | 67.6% |
| Dela | 103 | 153 | 67.3% | 1243 | 1843 | 67.4% |
| Dick | 1 | 2 | 50.0% | 19 | 21 | 90.5% |
| Greece (GHFR) | 30 | 41 | 73.2% | 673 | 938 | 71.7% |
| Guo | 83 | 133 | 62.4% | 589 | 884 | 66.6% |
| Ishimaru | 16 | 20 | 80.0% | 527 | 646 | 81.6% |
| Kau | 35 | 59 | 59.3% | 72 | 138 | 52.2% |
| Kent | 4 | 4 | 100.0% | 8 | 8 | 100.0% |
| Liu | 34 | 66 | 51.5% | 19 | 37 | 51.4% |
| Loggers All | 68 | 88 | 77.3% | 67 | 84 | 79.8% |
| Marshall | 13 | 19 | 68.4% | 37 | 61 | 60.7% |
| Neuman | 7115 | 10283 | 69.2% | 116527 | 155578 | 74.9% |
| Neuman | 5163 | 7069 | 73.0% | 40182 | 53042 | 75.8% |
| Novack | 543 | 818 | 66.4% | 2926 | 3815 | 76.7% |
| Ovidiu Cohort: Age 95 | 30 | 40 | 75.0% | 55 | 94 | 58.5% |
| Prommik All | 530 | 766 | 69.2% | 7528 | 10444 | 72.1% |
| Shabat | 4 | 4 | 100.0% | 13 | 19 | 68.4% |
| Tamulaitiene | 9 | 12 | 75.0% | 326 | 429 | 76.0% |
| Tan C | 149 | 233 | 63.9% | 551 | 781 | 70.6% |
| Tan L | 44 | 57 | 77.2% | 141 | 187 | 75.4% |
| Tan S | 540 | 727 | 74.3% | 1511 | 2029 | 74.5% |
| Tay | 82 | 114 | 71.9% | 194 | 226 | 85.8% |
| van Bremen | 2005 | 3065 | 65.4% | 60309 | 91865 | 65.6% |
| Wang Cohort: Age 90 | 19 | 32 | 59.4% | 128 | 173 | 74.0% |
| Wijnen Cohort: Frail | 59 | 91 | 64.8% | 877 | 1188 | 73.8% |
| Yang | 195 | 315 | 61.9% | 2487 | 4155 | 59.9% |
| Zielinski | 18 | 22 | 81.8% | 786 | 1228 | 64.0% |

### **Table S7:** ASA physical status: proportion of ASA physical status 3-5 patients

| **Study label** | **ASA 3-5 conservative** | **Total conservative** | **ASA 3-5 conservative proportion** | **ASA 3-5 operative** | **Total operative** | **ASA 3-5 operative proportion** |
| --- | --- | --- | --- | --- | --- | --- |
| Amrayev | 70 | 78 | 89.7% | 218 | 266 | 82.0% |
| Gregory | 19 | 22 | 86.4% | 48 | 80 | 60.0% |
| Guo | 90 | 133 | 67.7% | 257 | 634 | 40.5% |
| Liu | 32 | 67 | 47.8% | 17 | 37 | 45.9% |
| Loggers All | 84 | 88 | 95.5% | 83 | 84 | 98.8% |
| Tay | 69 | 114 | 60.5% | 119 | 226 | 52.7% |
| Zielinski | 11 | 22 | 50.0% | 372 | 1228 | 30.3% |

###

### **Table S8:** Comorbidity: summary statistics for reported Charlson Comorbidity Index

| **Study** | **Comorbidity non-operative** | **Comorbidity operative** |
| --- | --- | --- |
| Chlebeck All | 2.13 (1.29) | 1.5 (1.27) |
| Doshi | 6 | 5.6 |
| Kau | 5.92 (2.38) | 4.59 (1.88) |
| Loggers All | 3 (2, 5) | 3 (2, 5) |
| Neuman | 4.3 | 4.6 |
| Novack | 4.7 (1.50) | 4.4 (1.3) |
| Prommik All | 2.0 (1.8) | 1.6 (1.6) |
| Wang Cohort: Age 90 | 2 (1,2) | 1 (0,2) |

###

### **Table S9:** Proportion of patients with dementia

| **Study label** | **Dementia conservative** | **Total conservative** | **Dementia conservative proportion** | **Dementia operative** | **Total operative** | **Dementia operative proportion** |
| --- | --- | --- | --- | --- | --- | --- |
| Abeygunasekara | 10 | 73 | 13.7% | 2 | 107 | 1.9% |
| Adler | 999 | 1998 | 50.0% | 999 | 1998 | 50.0% |
| Chlebeck All | 47 | 77 | 61.0% | 283 | 1036 | 27.3% |
| De Virgilio-Salgado | 62 | 109 | 56.9% | 58 | 159 | 36.5% |
| Ishimaru | 10 | 20 | 50.0% | 247 | 646 | 38.2% |
| Kent | 0 | 4 | 0.0% | 4 | 8 | 50.0% |
| Loggers All | 83 | 88 | 94.3% | 75 | 84 | 89.3% |
| Mitchell | 2303 | 10307 | 22.3% | 16480 | 59063 | 27.9% |
| Neuman | 3928 | 10283 | 38.2% | 51030 | 155578 | 32.8% |
| Novack | 97 | 818 | 11.9% | 348 | 3815 | 9.1% |
| Prommik All | 208 | 1197 | 17.4% | 913 | 10431 | 8.8% |
| Romero Pisonero | 350 | 9052 | 3.9% | 126 | 8190 | 1.5% |
| Tan L | 11 | 57 | 19.3% | 24 | 187 | 12.8% |
| van Bremen | 1140 | 3065 | 37.2% | 13963 | 91865 | 15.2% |
| Wijnen Cohort: Frail | 48 | 91 | 52.7% | 317 | 1188 | 26.7% |
| Zielinski | 9 | 22 | 40.9% | 229 | 1229 | 18.6% |

### **Table S10:** Proportion of patients cancer

| **Study label** | **Cancer diagnosis conservative** | **Total conservative** | **Cancer conservative proportion** | **Cancer diagnosis operative** | **Total operative** | **Cancer operative proportion** |
| --- | --- | --- | --- | --- | --- | --- |
| De Virgilio-Salgado | 33 | 109 | 30.3% | 38 | 159 | 23.9% |
| Guo | 2 | 133 | 1.5% | 19 | 884 | 2.1% |
| Ishimaru | 4 | 20 | 20.0% | 60 | 646 | 9.3% |
| Novack | 34 | 818 | 4.2% | 96 | 3815 | 2.5% |
| Prommik All | 151 | 766 | 19.7% | 1042 | 10444 | 10.0% |
| Zielinski | 5 | 22 | 22.7% | 179 | 1228 | 14.6% |

### **Table S11:** Proportion of patients admitted from home

| **Study label** | **Admitted from home conservative** | **Total conservative** | **Home conservative proportion** | **Admitted from home operative** | **Total operative** | **Home operative proportion** |
| --- | --- | --- | --- | --- | --- | --- |
| Adler | 20174 | 23067 | 87.5% | 28958 | 33142 | 87.4% |
| Amrayev | 75 | 78 | 96.2% | 225 | 266 | 84.6% |
| Chlebeck All | 27 | 77 | 35.1% | 675 | 1036 | 65.2% |
| Cram Cohort: 1997 | 262 | 327 | 80.1% | 2608 | 3742 | 69.7% |
| Cram Cohort: 2002 | 153 | 225 | 68.0% | 2151 | 3275 | 65.7% |
| Cram Cohort: 2007 | 138 | 228 | 60.5% | 2792 | 3937 | 70.9% |
| Cram Cohort: 2012 | 143 | 200 | 71.5% | 2886 | 3753 | 76.9% |
| De Virgilio-Salgado | 74 | 109 | 67.9% | 136 | 159 | 85.5% |
| Ireland | 301 | 999 | 30.1% | 1543 | 999 | 154.5% |
| Kent | 2 | 4 | 50.0% | 1 | 8 | 12.5% |
| Loggers All | 0 | 88 | 0.0% | 0 | 84 | 0.0% |
| Rios-German | 270 | 999 | 27.0% | 13570 | 999 | 1358.4% |
| van Bremen | 776 | 3065 | 25.3% | 50077 | 91865 | 54.5% |
| Wijnen Cohort: Frail | 26 | 91 | 28.6% | 725 | 1188 | 61.0% |
| Zielinski | 12 | 22 | 54.5% | 688 | 1228 | 56.0% |

### **Table S12**: Ambulatory status on admission: proportion of patients independently mobile

| **Study label** | **Independently mobile conservative** | **Total conservative** | **Mobility conservative proportion** | **Independently mobile operative** | **Total operative** | **Mobility operative proportion** |
| --- | --- | --- | --- | --- | --- | --- |
| Berry | 49 | 468 | 10.5% | 830 | 2615 | 31.7% |
| Chow | 16 | 60 | 26.7% | 399 | 990 | 40.3% |
| De Virgilio-Salgado | 61 | 109 | 56.0% | 92 | 159 | 57.9% |
| Hagino Cohort: Age 70 | 7 | 9 | 77.8% | 42 | 46 | 91.3% |
| Hagino Cohort: Age 80 | 13 | 17 | 76.5% | 139 | 155 | 89.7% |
| Hagino Cohort: Age 90 | 8 | 18 | 44.4% | 130 | 162 | 80.2% |
| Hagino Cohort: Age 95 | 2 | 3 | 66.7% | 12 | 20 | 60.0% |
| Kau | 42 | 59 | 71.2% | 81 | 138 | 58.7% |
| Ko | 19 | 34 | 55.9% | 1412 | 1807 | 78.1% |
| Loggers All | 41 | 88 | 46.6% | 47 | 84 | 56.0% |
| Shabat | 2 | 4 | 50.0% | 11 | 19 | 57.9% |
| van Bremen | 600 | 3065 | 19.6% | 43341 | 91865 | 47.2% |
| Wijnen Cohort: Frail | 9 | 91 | 9.9% | 511 | 1188 | 43.0% |
| Zielinski | 17 | 22 | 77.3% | 1062 | 1228 | 86.5% |

### **Table S13:** Activities of daily living: proportion of patients independent in ADLs

| **Study label** | **Independent of ADLs conservative** | **Total conservative** | **Independent of ADLs conservative proportion** | **Independent of ADLs operative** | **Total operative** | **Independent of ADLs operative proportion** |
| --- | --- | --- | --- | --- | --- | --- |
| Berry | 347 | 468 | 74.1% | 2468 | 2615 | 94.4% |
| Loggers All | 0 | 88 | 0.0% | 2 | 84 | 2.4% |
| van Bremen | 680 | 3065 | 22.2% | 50494 | 91865 | 55.0% |

### **Table S14:** Ethnicity: proportion with majority ethnicity for the relevant country by study. Data are as reported and relevant to the study country as country majority ethnicity varies. E.g. White for UK, Chinese for Singapore.

| **Study label** | **Ethnicity majority conservative** | **Total conservative** | **Ethnicity majority conservative proportion** | **Ethnicity majority operative** | **Total operative** | **Ethnicity majority operative proportion** |
| --- | --- | --- | --- | --- | --- | --- |
| Berry | 385 | 468 | 82.3% | 2345 | 2615 | 89.7% |
| Fanuele | 3146 | 3350 | 93.9% | 128748 | 136845 | 94.1% |
| Kau | 53 | 59 | 89.8% | 120 | 128 | 93.8% |
| Marshall | 5 | 19 | 26.3% | 20 | 61 | 32.8% |
| Neuman | 9080 | 10283 | 88.3% | 143132 | 155578 | 92.0% |
| Tan C | 36 | 233 | 15.5% | 100 | 781 | 12.8% |
| Yang | 204 | 315 | 64.8% | 2522 | 3842 | 65.6% |

### **Table S15:** Fracture type: proportion of patients with intracapsular fracture

| **Study label** | **Intracapsular conservative** | **Total conservative** | **Intracapsular fracture conservative proportion** | **Intracapsular operative** | **Total operative** | **Intracapsular fracture operative proportion** |
| --- | --- | --- | --- | --- | --- | --- |
| Amrayev | 34 | 78 | 43.6% | 159 | 266 | 59.8% |
| Chaysri | 26 | 72 | 36.1% | 68 | 203 | 33.5% |
| Chlebeck All | 44 | 77 | 57.1% | 526 | 1036 | 50.8% |
| Cram Cohort: 1997 | 111 | 327 | 33.9% | 1692 | 3742 | 45.2% |
| Cram Cohort: 2002 | 90 | 225 | 40.0% | 1565 | 3275 | 47.8% |
| Cram Cohort: 2007 | 106 | 228 | 46.5% | 2045 | 3937 | 51.9% |
| Cram Cohort: 2012 | 86 | 200 | 43.0% | 1929 | 3753 | 51.4% |
| Daraphongsataporn | 213 | 331 | 64.4% | 177 | 722 | 24.5% |
| De Virgilio-Salgado | 55 | 109 | 50.5% | 72 | 159 | 45.3% |
| Dedovic | 16 | 32 | 50.0% | 2 | 34 | 5.9% |
| Dick | 2 | 2 | 100.0% | 11 | 21 | 52.4% |
| Dobre | 259 | 450 | 57.6% | 865 | 2292 | 37.7% |
| Fanuele | 1456 | 3350 | 43.5% | 48299 | 136845 | 35.3% |
| Gregory | 22 | 22 | 100.0% | 80 | 80 | 100.0% |
| Hagino | 11151 | 22740 | 49.0% | 167549 | 369264 | 45.4% |
| Hay | 8 | 9 | 88.9% | 55 | 143 | 38.5% |
| Ishimaru | 8 | 20 | 40.0% | 119 | 646 | 18.4% |
| Kau | 41 | 59 | 69.5% | 72 | 138 | 52.2% |
| Kent | 3 | 4 | 75.0% | 4 | 8 | 50.0% |
| Lee J | 32 | 93 | 34.4% | 461 | 997 | 46.2% |
| Liu | 67 | 67 | 100.0% | 37 | 37 | 100.0% |
| Loggers All | 54 | 88 | 61.4% | 45 | 84 | 53.6% |
| Longo | 86386 | 302693 | 28.5% | 419248 | 1187450 | 35.3% |
| Malhotra | 11 | 23 | 47.8% | 19 | 42 | 45.2% |
| Neuman | 6343 | 10280 | 61.7% | 73119 | 155574 | 47.0% |
| Ovidiu Cohort: Age 95 | 13 | 40 | 32.5% | 35 | 94 | 37.2% |
| Prommik All | 569 | 765 | 74.4% | 5112 | 10444 | 48.9% |
| Rowe | 67 | 142 | 47.2% | 490 | 1010 | 48.5% |
| Shabat | 2 | 4 | 50.0% | 2 | 19 | 10.5% |
| Takahashi | 15 | 34 | 44.1% | 85 | 194 | 43.8% |
| Tan C | 118 | 233 | 50.6% | 387 | 781 | 49.6% |
| Tan L | 26 | 57 | 45.6% | 105 | 187 | 56.1% |
| Tan S | 387 | 999 | 38.7% | 1081 | 999 | 108.2% |
| Tian M | 40 | 78 | 51.3% | 257 | 696 | 36.9% |
| van Bremen | 1796 | 3065 | 58.6% | 50005 | 91865 | 54.4% |
| Wang Cohort: Age 90 | 22 | 32 | 68.8% | 61 | 173 | 35.3% |
| Wijnen Cohort: Frail | 53 | 91 | 58.2% | 639 | 1188 | 53.8% |
| Zielinski | 22 | 1021 | 2.2% | 1228 | 2227 | 55.1% |

## **Cohort outcomes**

### Mortality

### **Table S16:** In-hospital mortality incidence

| **Study** | **Death in hospital days non-operative** | **Total in hospital days non-operative** | **Death in hospital days proportion non-operative** | **Death in hospital days operative** | **Total in hospital days operative** | **Death in hospital days proportion operative** |
| --- | --- | --- | --- | --- | --- | --- |
| Cram Cohort: 2012 | 251 | 1307 | 19.2% | 834 | 18319 | 4.6% |
| De Virgilio-Salgado | 10 | 109 | 9.2% | 3 | 159 | 1.9% |
| Dick | 2 | 2 | 100.0% | 6 | 21 | 28.6% |
| Dobre | 52 | 450 | 11.6% | 65 | 2292 | 2.8% |
| Hay | 3 | 9 | 33.3% | 16 | 143 | 11.2% |
| Kawaji | 1 | 22 | 4.5% | 8 | 208 | 3.8% |
| Kent | 2 | 4 | 50.0% | 2 | 8 | 25.0% |
| Kir | 12 | 28 | 42.9% | 24 | 682 | 3.5% |
| Lotan Cohort: 2010 | 28 | 285 | 9.8% | 65 | 3195 | 2.0% |
| Lotan Cohort: 2011 | 31 | 287 | 10.8% | 70 | 3211 | 2.2% |
| Lotan Cohort: 2012 | 17 | 220 | 7.7% | 72 | 3269 | 2.2% |
| Lotan Cohort: 2013 | 20 | 208 | 9.6% | 76 | 3465 | 2.2% |
| Lotan Cohort: 2014 | 24 | 194 | 12.4% | 110 | 3642 | 3.0% |
| Lotan Cohort: 2015 | 23 | 238 | 9.7% | 116 | 4017 | 2.9% |
| Lotan Cohort: 2016 | 24 | 251 | 9.6% | 79 | 4027 | 2.0% |
| Lotan Cohort: 2017 | 24 | 192 | 12.5% | 103 | 3817 | 2.7% |
| Lotan Cohort: 2018 | 29 | 209 | 13.9% | 116 | 3844 | 3.0% |
| Lotan Cohort: 2019 | 23 | 222 | 10.4% | 93 | 4048 | 2.3% |
| Novack | 89 | 818 | 10.9% | 120 | 3815 | 3.1% |
| Shoda | 1309 | 13907 | 9.4% | 1372 | 66893 | 2.1% |
| Tan C | 14 | 233 | 6.0% | 0 | 781 | 0.0% |
| Johansen | 694 | 1427 | 48.6% | 3914 | 59369 | 6.6% |
| Krusic | 10 | 34 | 29.4% | 17 | 461 | 3.7% |
| Sanz-Reig | 14 | 21 | 66.7% | 24 | 310 | 7.7% |
| Yamauchi | 129 | 839 | 15.4% | 170 | 3220 | 5.3% |
| Teo (NZ) | 4 | 6 | 66.7% | 9 | 138 | 6.5% |
| Doshi | 3 | 40 | 7.5% | 3 | 179 | 1.7% |
| Declarador | 5 | 108 | 4.6% | 4 | 446 | 0.9% |
| Stolnicki : 2008 | 235 | 2653 | 8.9% | 867 | 25260 | 3.4% |
| Stolnicki : 2017 | 218 | 1945 | 11.2% | 1564 | 37987 | 4.1% |
| Tamulaitiene | 3 | 12 | 25.0% | 6 | 429 | 1.4% |
| Valaviciene | 3 | 5 | 60.0% | 2 | 59 | 3.4% |
| Bugeja | 3 | 5 | 60.0% | 24 | 449 | 5.3% |
| Duarte-Flores | 31 | 46 | 67.4% | 14 | 995 | 1.4% |

### **Table S17:** 7-day mortality incidence

| **Study** | **Death 7 days non-operative** | **Total 7 days non-operative** | **Death 7 days proportion non-operative** | **Death 7 days operative** | **Total 7 days operative** | **Death 7 days proportion operative** |
| --- | --- | --- | --- | --- | --- | --- |
| Dick | 2 | 2 | 100.0% | 3 | 21 | 14.3% |
| Loggers All | 2 | 88 | 2.3% | 0 | 84 | 0% |
| Neuman | 1933 | 10283 | 18.8% | 2489 | 155578 | 1.6% |

### **Table S18:** 30-day mortality incidence

| **Study** | **Death 30 days non-operative** | **Total 30 days non-operative** | **Death 30 days proportion non-operative** | **Death 30days operative** | **Total 30 days operative** | **Death 30 days proportion operative** |
| --- | --- | --- | --- | --- | --- | --- |
| Castronuovo | 181 | 1117 | 16.2% | 256 | 5869 | 4.4% |
| De Virgilio-Salgado | 19 | 109 | 17.4% | 8 | 159 | 5.0% |
| Dick | 2 | 2 | 100.0% | 6 | 21 | 28.6% |
| Dobre | 104 | 450 | 23.1% | 159 | 2292 | 6.9% |
| Gregory | 7 | 22 | 31.8% | 3 | 82 | 3.7% |
| Kau | 11 | 59 | 18.6% | 5 | 138 | 3.6% |
| Kawaji | 1 | 22 | 4.5% | 3 | 208 | 1.4% |
| Kent | 2 | 4 | 50.0% | 3 | 8 | 37.5% |
| Liu | 9 | 67 | 13.4% | 3 | 37 | 8.1% |
| Loggers All | 74 | 88 | 84.1% | 22 | 84 | 26.2% |
| Neuman | 3773 | 10283 | 36.7% | 10579 | 155578 | 6.8% |
| Novack | 109 | 818 | 13.3% | 169 | 3815 | 4.4% |
| Ooi | 2 | 38 | 5.3% | 1 | 46 | 2.2% |
| Ovidiu Cohort: Age 95 | 9 | 40 | 22.5% | 16 | 94 | 17.0% |
| Prommik All | 183 | 766 | 23.9% | 690 | 10444 | 6.6% |
| Shabat | 3 | 4 | 75.0% | 1 | 19 | 5.3% |
| Tan C | 20 | 233 | 8.6% | 0 | 781 | 0.0% |
| Vigano | 1480 | 7624 | 19.4% | 3955 | 64296 | 6.2% |
| Wijnen Cohort: Frail | 79 | 91 | 86.8% | 83 | 1188 | 7.0% |
| Li | 20910 | 123002 | 17.0% | 16496 | 181277 | 9.1% |
| Wang Cohort: Age 90 | 3 | 32 | 9.4% | 3 | 173 | 1.7% |
| Dedovic | 7 | 32 | 21.9% | 5 | 34 | 14.7% |
| Adler | 699 | 23067 | 3.0% | 3204 | 33142 | 9.7% |
| Kristan | 8 | 52 | 15.4% | 33 | 643 | 5.1% |
| Greece (GHFR) | 7 | 41 | 17.1% | 66 | 938 | 7.0% |
| Tan S | 54 | 727 | 7.4% | 34 | 2029 | 1.7% |
| Moulton | 14 | 36 | 38.9% | 36 | 600 | 6.0% |

### **Table S19:** 90-day mortality incidence

| **Study** | **Death 90 days non-operative** | **Total 90 days non-operative** | **Death 90 days proportion non-operative** | **Death 90 days operative** | **Total 90 days operative** | **Death 90 days proportion operative** |
| --- | --- | --- | --- | --- | --- | --- |
| Dick | 2 | 2 | 100.0% | 8 | 21 | 38.1% |
| Loggers All | 80 | 88 | 90.9% | 34 | 84 | 40.5% |
| Ovidiu Cohort: Age 95 | 16 | 40 | 40.0% | 32 | 94 | 34.0% |
| Prommik All | 330 | 766 | 43.1% | 1646 | 10444 | 15.8% |
| Shabat | 4 | 4 | 100.0% | 5 | 19 | 26.3% |
| Vigano | 2130 | 7624 | 27.9% | 6943 | 64296 | 10.8% |
| Rowe | 23 | 142 | 16.2% | 56 | 1010 | 5.5% |
| Berry | 205 | 468 | 43.8% | 586 | 2615 | 22.4% |
| Adler | 5392 | 23067 | 23.4% | 6658 | 33142 | 20.1% |
| Amphansap | 4 | 21 | 19.0% | 3 | 99 | 3.0% |

### **Table S20:** 180-day mortality incidence

| **Study** | **Death 180 days non-operative** | **Total 180 days non-operative** | **Death 180 days proportion non-operative** | **Death 180 days operative** | **Total 180 days operative** | **Death 180 days proportion operative** |
| --- | --- | --- | --- | --- | --- | --- |
| Castronuovo | 359 | 1117 | 32.1% | 418 | 5869 | 7.1% |
| Cram Cohort: 2012 | 547 | 1307 | 41.9% | 3086 | 18319 | 16.8% |
| Dick | 2 | 2 | 100.0% | 10 | 20 | 50.0% |
| Loggers All | 83 | 88 | 94.3% | 40 | 84 | 47.6% |
| Ooi | 11 | 38 | 28.9% | 11 | 46 | 23.9% |
| Ovidiu Cohort: Age 95 | 20 | 40 | 50.0% | 36 | 94 | 38.3% |
| Prommik All | 455 | 766 | 59.4% | 2236 | 10444 | 21.4% |
| Shabat | 4 | 4 | 100.0% | 8 | 19 | 42.1% |
| Vigano | 2553 | 7624 | 33.5% | 10985 | 64296 | 17.1% |
| Rowe | 33 | 142 | 23.2% | 78 | 1010 | 7.7% |
| Cancio | 805 | 1650 | 48.8% | 4508 | 28864 | 15.6% |
| Dedovic | 18 | 32 | 56.3% | 8 | 34 | 23.5% |
| Berry | 250 | 468 | 53.4% | 818 | 2615 | 31.3% |
| Adler | 7326 | 23067 | 31.8% | 9562 | 33142 | 28.9% |
| Amphansap | 4 | 21 | 19.0% | 4 | 99 | 4.0% |
| Jang B-W | 6 | 6 | 100.0% | 15 | 56 | 26.8% |

### **Table S21:** 1-year mortality incidence

| **Study** | **Death one year non-operative** | **Total one year non-operative** | **Death one year proportion non-operative** | **Death one year operative** | **Total one year operative** | **Death one year proportion operative** |
| --- | --- | --- | --- | --- | --- | --- |
| Abeygunasekara | 27 | 73 | 37.0% | 6 | 107 | 5.6% |
| Amrayev | 51 | 78 | 65.4% | 55 | 266 | 20.7% |
| Chaysri | 30 | 72 | 41.7% | 28 | 203 | 13.8% |
| Chow | 32 | 60 | 53.3% | 124 | 990 | 12.5% |
| Cram Cohort: 2012 | 543 | 1307 | 41.5% | 4172 | 18319 | 22.8% |
| De Virgilio-Salgado | 53 | 109 | 48.6% | 37 | 159 | 23.3% |
| Dick | 2 | 2 | 100.0% | 16 | 20 | 80.0% |
| Dobre | 254 | 450 | 56.4% | 561 | 2292 | 24.5% |
| Gregory | 11 | 22 | 50.0% | 23 | 80 | 28.7% |
| Hay | 6 | 9 | 66.7% | 73 | 143 | 51.0% |
| Heyes All | 9 | 12 | 75.0% | 58 | 372 | 15.6% |
| Kau | 19 | 59 | 32.2% | 10 | 138 | 7.2% |
| Kent | 2 | 4 | 50.0% | 4 | 8 | 50.0% |
| Liu | 28 | 67 | 41.8% | 7 | 37 | 18.9% |
| Lotan Cohort: 2019 | 999 | 999 | 100.0% | 999 | 999 | 100.0% |
| Malhotra | 4 | 23 | 17.4% | 6 | 42 | 14.3% |
| Novack | 296 | 818 | 36.2% | 742 | 3815 | 19.4% |
| Ooi | 17 | 38 | 44.7% | 14 | 46 | 30.4% |
| Ovidiu Cohort: Age 95 | 25 | 40 | 62.5% | 43 | 94 | 45.7% |
| Prommik All | 470 | 766 | 61.4% | 2880 | 10444 | 27.6% |
| Shabat | 4 | 4 | 100.0% | 8 | 19 | 42.1% |
| Tan C | 77 | 233 | 33.0% | 70 | 781 | 9.0% |
| Tay | 34 | 114 | 29.8% | 15 | 226 | 6.6% |
| Vigano | 3067 | 7624 | 40.2% | 14699 | 64296 | 22.9% |
| Wijnen Cohort: Frail | 90 | 91 | 98.9% | 333 | 1188 | 28.0% |
| Hwang | 51 | 116 | 44.0% | 83 | 691 | 12.0% |
| Rowe | 48 | 142 | 33.8% | 124 | 1010 | 12.3% |
| Wang Cohort: Age 90 | 5 | 32 | 15.6% | 21 | 173 | 12.1% |
| Cancio | 919 | 1650 | 55.7% | 6421 | 28864 | 22.2% |
| Ishimaru | 9 | 20 | 45.0% | 53 | 646 | 8.2% |
| Yang | 94 | 315 | 29.8% | 786 | 3842 | 20.5% |
| Berry | 296 | 468 | 63.2% | 1109 | 2615 | 42.4% |
| Daraphongsataporn | 89 | 166 | 53.6% | 264 | 1014 | 26.0% |
| Giummarra | 109 | 307 | 35.5% | 1190 | 4499 | 26.5% |
| Ahmad | 1 | 4 | 25.0% | 8 | 134 | 6.0% |
| Declarador | 24 | 108 | 22.2% | 33 | 446 | 7.4% |
| Lo | 743 | 1000 | 74.3% | 4019 | 6646 | 60.5% |
| Amphansap | 5 | 21 | 23.8% | 6 | 99 | 6.1% |
| Guo | 59 | 133 | 44.4% | 71 | 884 | 8.0% |
| Barahona | 3126 | 7443 | 42.0% | 4688 | 23532 | 19.9% |
| Barahona | 141 | 235 | 60.0% | 601 | 3005 | 20.0% |
| Kristan | 24 | 52 | 46.2% | 119 | 643 | 18.5% |
| Lee A | 2 | 3 | 66.7% | 17 | 67 | 25.4% |
| Moulton | 22 | 36 | 61.1% | 63 | 600 | 10.5% |
| Jang B-W | 6 | 6 | 100.0% | 22 | 56 | 39.3% |

### **Table S22:** 2-year mortality incidence

| **Study** | **Death two years non-operative** | **Total two years non-operative** | **Death two years proportion non-operative** | **Death two years operative** | **Total two years operative** | **Death two years proportion operative** |
| --- | --- | --- | --- | --- | --- | --- |
| De Virgilio-Salgado | 69 | 109 | 63.3% | 58 | 159 | 36.5% |
| Dick | 2 | 2 | 100.0% | 18 | 20 | 90.0% |
| Liu | 33 | 67 | 49.3% | 10 | 37 | 27.0% |
| Ooi | 22 | 38 | 57.9% | 19 | 46 | 41.3% |
| Ovidiu Cohort: Age 95 | 31 | 40 | 77.5% | 53 | 94 | 56.4% |
| Prommik All | 538 | 766 | 70.2% | 3947 | 10444 | 37.8% |
| Tay | 52 | 114 | 45.6% | 31 | 226 | 13.7% |
| Vigano | 3742 | 7624 | 49.1% | 20675 | 64296 | 32.2% |
| Hwang | 71 | 116 | 61.2% | 247 | 691 | 35.7% |
| Wang Cohort: Age 90 | 8 | 32 | 25.0% | 36 | 173 | 20.8% |
| Cancio | 1020 | 1650 | 61.8% | 9543 | 28864 | 33.1% |
| Berry | 357 | 468 | 76.3% | 1535 | 2615 | 58.7% |
| Guo | 64 | 133 | 48.1% | 150 | 884 | 17.0% |

### **Table S23:** 3-year mortality incidence

| **Study** | **Death three years non-operative** | **Total three years non-operative** | **Death three years proportion non-operative** | **Death three years operative** | **Total three years operative** | **Death three years proportion operative** |
| --- | --- | --- | --- | --- | --- | --- |
| Dick | 2 | 2 | 100.0% | 19 | 20 | 95.0% |
| Liu | 41 | 67 | 61.2% | 14 | 37 | 37.8% |
| Prommik All | 574 | 766 | 74.9% | 4817 | 10444 | 46.1% |

### **Table S24:** 5-year mortality incidence

| **Study** | **Death five years non-operative** | **Total five years non-operative** | **Death five years proportion non-operative** | **Death five years operative** | **Total five years operative** | **Death five years proportion operative** |
| --- | --- | --- | --- | --- | --- | --- |
| Dick | 2 | 2 | 100.0% | 20 | 20 | 100.0% |
| Liu | 49 | 67 | 73.1% | 18 | 37 | 48.6% |

###

### **Other outcomes**

###

### **Table S25:** Duration of hospital stay; days

| **Study** | **Length of stay non-operative** | **Length of stay operative** |
| --- | --- | --- |
| De Virgilio-Salgado | 6.9 (9.2) | 9.3 (7.6) |
| Dick | 6.5 (6.4) | 25.5 (12.9) |
| Dobre | 7.67 | 10.94 |
| Gregory | 27 (4, 69) | 25 (3-119) |
| Liu | 8 (0, 17) | 18 (0, 59) |
| Loggers All | 2 (2, 3) | 6 (4,7) |
| Novack | 8 (9.65) | 9.7 (6.95) |
| Ovidiu Cohort: Age 95 | 7.25 (7.33) | 15.0 (10.7) |
| Tan C | 12.8 | 13.1 |
| Tay | 14.1 | 14.3 |
| Tan L | 19.25 | 15.97 |
| Wang Cohort: Age 90 | 8 (1, 18,5) | 8 (6, 13) |
| Doshi | 6 | 5.6 |
| Amphansap | 25.86 | 23.16 |
| Stolnicki : 2008 | 9 | 8.5 |
| Stolnicki : 2017 | 9.1 | 9.3 |
| Tan S | 22.4 | 15.7 |

### **Table S26**: Discharge destination: proportion of patients returning to own home or nursing home

| **Study** | **Home conservative** | **Return to own residence conservative** | **Nursing home conservative** | **Home operative** | **Return to own residence operative** | **Nursing home operative** |
| --- | --- | --- | --- | --- | --- | --- |
| Amrayev | - | 27 | - | - | 211 | - |
| Cram Cohort: 2012 | 532 | - | 232 | 9320 | - | 4827 |
| De Virgilio-Salgado | 66 | - | 23 | 44 | - | 18 |
| Kau | 17 | - | 20 | 19 | - | 12 |
| Kent | 1 | - | 1 | 0 | - | 6 |
| Tay | 58 | - | 25 | 72 | - | 13 |
| Adler | 1573 | - | - | 2068 | - | - |
| Tan S | 397 | - | 239 | 1011 | - | 369 |

### **Table S27:** Ambulatory status at discharge

| **Study** | **Ambulatory on discharge non-operative** | **Ambulatory on discharge operative** |
| --- | --- | --- |
| Hagino Cohort: Age 70 | 5 | 38 |
| Hagino Cohort: Age 80 | 4 | 109 |
| Hagino Cohort: Age 90 | 3 | 83 |
| Hagino Cohort: Age 95 | 0 | 5 |
| Malhotra | 1 | 20 |
| Ooi | 3 | 17 |
| Shabat | 0 | 4 |

### **Table S28:** Delirium: proportion of patients with delirium

| **Study** | **Delirium non-operative** | **Delirium operative** |
| --- | --- | --- |
| Liu | 30 | 14 |
| Loggers All | 10 | 27 |
| Tan C | 12 | 86 |
| Adler | 3694 | 5443 |

## **Sensitivity analyses**

Columns marked * are analyses with outlier studies removed

### **Table S29:** Sensitivity analyses for proportions of patients receiving non-operative management. Results are reported by cohort type, region and with outliers removed.

| **Comparison** | **Metric** | **Event** | **Group** | **Cohort** | **Number of cohorts** | **Total events** | **Total cohort** | **Estimate - all** | **Total events*** | **Total cohort*** | **Estimate*** |
| --- | --- | --- | --- | --- | --- | --- | --- | --- | --- | --- | --- |
| A | Non-op rate | Conservative management | All | Unselected | 262 | 778515 | 9865523 | 0.083 (0.072 - 0.097) | 645603 | 9517467 | 0.078 (0.068 - 0.091) |
| A | Non-op rate | Conservative management | All | Admin / registries | 145 | 761262 | 9748664 | 0.074 (0.058 - 0.094) | 325080 | 7911381 | 0.06 (0.048 - 0.073) |
| A | Non-op rate | Conservative management | All | High risk | 27 | 34871 | 157611 | 0.192 (0.132 - 0.27) | 11737 | 101298 | 0.171 (0.118 - 0.241) |
| A | Non-op rate | Conservative management | E Asia and Pacific | Unselected | 89 | 186109 | 1126312 | 0.088 (0.07 - 0.111) | 62952 | 821703 | 0.084 (0.067 - 0.104) |
| A | Non-op rate | Conservative management | Europe and Central Asia | Unselected | 96 | 369339 | 2957273 | 0.066 (0.051 - 0.087) | 368360 | 2953714 | 0.066 (0.051 - 0.087) |
| A | Non-op rate | Conservative management | Latin America & the Caribbean | Unselected | 9 | 12471 | 104588 | 0.093 (0.059 - 0.144) | 4793 | 70373 | 0.081 (0.051 - 0.126) |
| A | Non-op rate | Conservative management | Middle East and N Africa | Unselected | 22 | 5377 | 69217 | 0.1 (0.069 - 0.143) | 4559 | 64584 | 0.088 (0.062 - 0.122) |
| A | Non-op rate | Conservative management | N America | Unselected | 37 | 204471 | 5603829 | 0.068 (0.042 - 0.108) | 204191 | 5602789 | 0.052 (0.039 - 0.068) |
| A | Non-op rate | Conservative management | S Asia | Unselected | 7 | 568 | 2108 | 0.163 (0.07 - 0.335) | 568 | 2108 | 0.163 (0.07 - 0.335) |
| A | Non-op rate | Conservative management | Sub-saharan Africa | Unselected | 2 | 180 | 2196 | 0.1 (0.056 - 0.17) | 180 | 2196 | 0.1 (0.056 - 0.17) |
| A | Non-op rate | Conservative management | E Asia and Pacific | Admin / registries | 27 | 180979 | 1085680 | 0.08 (0.049 - 0.125) | 57977 | 781401 | 0.069 (0.046 - 0.102) |
| A | Non-op rate | Conservative management | Europe and Central Asia | Admin / registries | 61 | 358491 | 2893296 | 0.059 (0.041 - 0.085) | 53681 | 1396168 | 0.048 (0.035 - 0.066) |
| A | Non-op rate | Conservative management | Latin America & the Caribbean | Admin / registries | 4 | 12088 | 99862 | 0.092 (0.033 - 0.23) | 4645 | 68887 | 0.06 (0.037 - 0.096) |
| A | Non-op rate | Conservative management | Middle East and N Africa | Admin / registries | 19 | 5200 | 67415 | 0.094 (0.05 - 0.17) | 4382 | 62782 | 0.071 (0.049 - 0.102) |
| A | Non-op rate | Conservative management | N America | Admin / registries | 33 | 204200 | 5601395 | 0.066 (0.036 - 0.117) | 204091 | 5601127 | 0.053 (0.038 - 0.073) |
| A | Non-op rate | Conservative management | S Asia | Admin / registries | 1 | 304 | 1016 | 0.299 (0.272 - 0.328) | 304 | 1016 | 0.299 (0.272 - 0.328) |
| A | Non-op rate | Conservative management | E Asia and Pacific | High risk | 9 | 3329 | 23571 | 0.231 (0.128 - 0.381) | 3262 | 23467 | 0.189 (0.115 - 0.295) |
| A | Non-op rate | Conservative management | Europe and Central Asia | High risk | 12 | 610 | 10391 | 0.184 (0.09 - 0.34) | 610 | 10391 | 0.184 (0.09 - 0.34) |
| A | Non-op rate | Conservative management | Middle East and N Africa | High risk | 1 | 4 | 23 | 0.174 (0.067 - 0.382) | 4 | 23 | 0.174 (0.067 - 0.382) |
| A | Non-op rate | Conservative management | N America | High risk | 4 | 30919 | 123560 | 0.161 (0.073 - 0.321) | 7852 | 67351 | 0.111 (0.074 - 0.163) |
| A | Non-op rate | Conservative management | Sub-saharan Africa | High risk | 1 | 9 | 66 | 0.136 (0.073 - 0.242) | 9 | 66 | 0.136 (0.073 - 0.242) |

### **Table S30:** Sensitivity analyses for patient characteristics. Results are reported by cohort type, region and with outliers removed.

| **Comparison** | **Metric** | **Event** | **Group** | **Cohort** | **Number of cohorts** | **Events conservative** | **Total conservative** | **Events operative** | **Total operative** | **Estimate - all** | **Number of cohorts*** | **Events conservative*** | **Total conservative*** | **Events operative*** | **Total operative*** | **Estimate*** |
| --- | --- | --- | --- | --- | --- | --- | --- | --- | --- | --- | --- | --- | --- | --- | --- | --- |
| B | Age | Mean age (years) | All | Unselected | 23 | NA | 7083 | NA | 130207 | 2.65 (1.7 - 3.6) | 22 | NA | 6633 | NA | 127915 | 2.79 (1.84 - 3.75) |
| B | Age | Mean age (years) | All | Admin / registries | 10 | NA | 5839 | NA | 122918 | 2.35 (1.2 - 3.5) | 9 | NA | 5073 | NA | 112474 | 2.47 (1.2 - 3.74) |
| B | Age | Mean age (years) | All | High risk | 10 | NA | 24138 | NA | 41129 | 0.05 (-0.76 - 0.86) | 8 | NA | 23732 | NA | 36099 | -0.02 (-0.64 - 0.6) |
| B | Age | Mean age (years) | E Asia and Pacific | Unselected | 8 | NA | 520 | NA | 3453 | 2.405 (0.959 - 3.852) | 8 | NA | 520 | NA | 3453 | 2.405 (0.959 - 3.852) |
| B | Age | Mean age (years) | Europe and Central Asia | Unselected | 6 | NA | 4412 | NA | 106177 | 3.466 (1.384 - 5.548) | 5 | NA | 3962 | NA | 103885 | 4.216 (2.343 - 6.088) |
| B | Age | Mean age (years) | Middle East and N Africa | Unselected | 2 | NA | 912 | NA | 4568 | -0.26 (-0.797 - 0.276) | 2 | NA | 912 | NA | 4568 | -0.26 (-0.797 - 0.276) |
| B | Age | Mean age (years) | N America | Unselected | 6 | NA | 1166 | NA | 15902 | 2.424 (1.666 - 3.182) | 6 | NA | 1166 | NA | 15902 | 2.424 (1.666 - 3.182) |
| B | Age | Mean age (years) | S Asia | Unselected | 1 | NA | 73 | NA | 107 | 8.4 (5.93 - 10.87) | 1 | NA | 73 | NA | 107 | 8.4 (5.93 - 10.87) |
| B | Age | Mean age (years) | E Asia and Pacific | Admin / registries | 1 | NA | 60 | NA | 990 | 1.4 (-1.19 - 3.99) | 1 | NA | 60 | NA | 990 | 1.4 (-1.19 - 3.99) |
| B | Age | Mean age (years) | Europe and Central Asia | Admin / registries | 3 | NA | 3872 | NA | 103247 | 3.724 (1.286 - 6.162) | 2 | NA | 3106 | NA | 92803 | 5 (4.68 - 5.32) |
| B | Age | Mean age (years) | Middle East and N Africa | Admin / registries | 1 | NA | 818 | NA | 3815 | -0.3 (-0.865 - 0.265) | 1 | NA | 818 | NA | 3815 | -0.3 (-0.865 - 0.265) |
| B | Age | Mean age (years) | N America | Admin / registries | 5 | NA | 1089 | NA | 14866 | 2.292 (1.366 - 3.219) | 5 | NA | 1089 | NA | 14866 | 2.292 (1.366 - 3.219) |
| B | Age | Mean age (years) | E Asia and Pacific | High risk | 2 | NA | 99 | NA | 210 | -0.248 (-0.974 - 0.477) | 2 | NA | 99 | NA | 210 | -0.248 (-0.974 - 0.477) |
| B | Age | Mean age (years) | Europe and Central Asia | High risk | 4 | NA | 185 | NA | 1301 | 0.352 (-1.505 - 2.208) | 3 | NA | 94 | NA | 113 | -0.747 (-1.43 - -0.065) |
| B | Age | Mean age (years) | Middle East and N Africa | High risk | 1 | NA | 4 | NA | 19 | -0.8 (-2.491 - 0.891) | 1 | NA | 4 | NA | 19 | -0.8 (-2.491 - 0.891) |
| B | Age | Mean age (years) | N America | High risk | 3 | NA | 23850 | NA | 39599 | 0.167 (-1.335 - 1.668) | 2 | NA | 23535 | NA | 35757 | 0.73 (0.523 - 0.937) |
| C | Sex | Female | All | Unselected | 26 | 12295 | 18084 | 207447 | 289896 | 0.97 (0.93 - 1.02) | 22 | 9050 | 13301 | 135693 | 182283 | 0.97 (0.91 - 1.02) |
| C | Sex | Female | All | Admin / registries | 10 | 10922 | 16122 | 199329 | 278496 | 0.94 (0.9 - 0.97) | 7 | 8217 | 12066 | 129086 | 172912 | 0.9 (0.87 - 0.94) |
| C | Sex | Female | All | High risk | 13 | 23052 | 31278 | 69892 | 94612 | 1.01 (0.97 - 1.05) | 10 | 17800 | 24078 | 28778 | 40288 | 1.02 (1.01 - 1.03) |
| C | Sex | Female | E Asia and Pacific | Unselected | 11 | 1118 | 1607 | 5470 | 7481 | 0.986 (0.89 - 1.092) | 10 | 578 | 880 | 3959 | 5452 | 0.985 (0.878 - 1.105) |
| C | Sex | Female | Europe and Central Asia | Unselected | 6 | 2643 | 3984 | 69793 | 105170 | 0.993 (0.96 - 1.027) | 4 | 108 | 153 | 1956 | 2861 | 1.079 (0.954 - 1.221) |
| C | Sex | Female | Middle East and N Africa | Unselected | 1 | 543 | 818 | 2926 | 3815 | 0.865 (0.822 - 0.912) | 1 | 543 | 818 | 2926 | 3815 | 0.865 (0.822 - 0.912) |
| C | Sex | Female | N America | Unselected | 6 | 7829 | 11449 | 127925 | 171480 | 0.931 (0.89 - 0.974) | 5 | 7659 | 11224 | 125519 | 168205 | 0.923 (0.911 - 0.935) |
| C | Sex | Female | S Asia | Unselected | 1 | 59 | 73 | 90 | 107 | 0.961 (0.836 - 1.104) | 1 | 59 | 73 | 90 | 107 | 0.961 (0.836 - 1.104) |
| C | Sex | Female | Sub-saharan Africa | Unselected | 1 | 103 | 153 | 1243 | 1843 | 0.998 (0.89 - 1.12) | 1 | 103 | 153 | 1243 | 1843 | 0.998 (0.89 - 1.12) |
| C | Sex | Female | E Asia and Pacific | Admin / registries | 1 | 34 | 60 | 702 | 990 | 0.799 (0.638 - 1.001) | 1 | 34 | 60 | 702 | 990 | 0.799 (0.638 - 1.001) |
| C | Sex | Female | Europe and Central Asia | Admin / registries | 3 | 2565 | 3872 | 68510 | 103247 | 0.986 (0.955 - 1.017) | 1 | 30 | 41 | 673 | 938 | 1.02 (0.844 - 1.233) |
| C | Sex | Female | Middle East and N Africa | Admin / registries | 1 | 543 | 818 | 2926 | 3815 | 0.865 (0.822 - 0.912) | 1 | 543 | 818 | 2926 | 3815 | 0.865 (0.822 - 0.912) |
| C | Sex | Female | N America | Admin / registries | 5 | 7780 | 11372 | 127191 | 170444 | 0.933 (0.888 - 0.981) | 4 | 7610 | 11147 | 124785 | 167169 | 0.923 (0.911 - 0.935) |
| C | Sex | Female | E Asia and Pacific | High risk | 2 | 53 | 98 | 147 | 210 | 0.872 (0.687 - 1.106) | 2 | 53 | 98 | 147 | 210 | 0.872 (0.687 - 1.106) |
| C | Sex | Female | Europe and Central Asia | High risk | 6 | 186 | 257 | 1049 | 1429 | 1.008 (0.879 - 1.155) | 4 | 97 | 126 | 117 | 147 | 0.992 (0.871 - 1.129) |
| C | Sex | Female | Middle East and N Africa | High risk | 1 | 4 | 4 | 13 | 19 | 1.444 (1.074 - 1.942) | 1 | 4 | 4 | 13 | 19 | 1.444 (1.074 - 1.942) |
| C | Sex | Female | N America | High risk | 4 | 22809 | 30919 | 68683 | 92954 | 1.011 (0.97 - 1.054) | 3 | 17646 | 23850 | 28501 | 39912 | 1.025 (1.014 - 1.035) |
| D | ASA | ASA 1 or 2 | All | Unselected | 5 | 107 | 369 | 1418 | 2434 | 0.64 (0.5 - 0.84) | 3 | 88 | 269 | 514 | 940 | 0.6 (0.36 - 0.99) |
| D | ASA | ASA 1 or 2 | All | High risk | 2 | 39 | 155 | 21 | 121 | 1.25 (0.44 - 3.54) | 3 | 88 | 269 | 514 | 940 | 0.6 (0.36 - 0.99) |
| D | ASA | ASA 1 or 2 | E Asia and Pacific | Unselected | 2 | 87 | 247 | 484 | 860 | 0.665 (0.427 - 1.034) | 2 | 87 | 247 | 484 | 860 | 0.665 (0.427 - 1.034) |
| D | ASA | ASA 1 or 2 | Europe and Central Asia | Unselected | 3 | 20 | 122 | 934 | 1574 | 0.637 (0.447 - 0.908) | 1 | 1 | 22 | 30 | 80 | 0.121 (0.017 - 0.84) |
| D | ASA | ASA 1 or 2 | E Asia and Pacific | High risk | 1 | 35 | 67 | 20 | 37 | 0.966 (0.664 - 1.406) | 1 | 35 | 67 | 20 | 37 | 0.966 (0.664 - 1.406) |
| D | ASA | ASA 1 or 2 | Europe and Central Asia | High risk | 1 | 4 | 88 | 1 | 84 | 3.818 (0.436 - 33.468) | 1 | 4 | 88 | 1 | 84 | 3.818 (0.436 - 33.468) |
| E | Comorbidity | Mean comorbidity score | All | Unselected | 4 | NA | 1720 | NA | 15433 | 0.51 (0.24 - 0.78) | 4 | NA | 1720 | NA | 15433 | 0.51 (0.24 - 0.78) |
| E | Comorbidity | Mean comorbidity score | All | Admin / registries | 2 | NA | 1584 | NA | 14259 | 0.34 (0.25 - 0.44) | 2 | NA | 1584 | NA | 14259 | 0.34 (0.25 - 0.44) |
| E | Comorbidity | Mean comorbidity score | All | High risk | 2 | NA | 120 | NA | 257 | 0.55 (-0.43 - 1.52) | 2 | NA | 120 | NA | 257 | 0.55 (-0.43 - 1.52) |
| E | Comorbidity | Mean comorbidity score | E Asia and Pacific | Unselected | 1 | NA | 59 | NA | 138 | 1.3 (0.61 - 1.99) | 1 | NA | 59 | NA | 138 | 1.3 (0.61 - 1.99) |
| E | Comorbidity | Mean comorbidity score | Europe and Central Asia | Unselected | 1 | NA | 766 | NA | 10444 | 0.4 (0.269 - 0.531) | 1 | NA | 766 | NA | 10444 | 0.4 (0.269 - 0.531) |
| E | Comorbidity | Mean comorbidity score | Middle East and N Africa | Unselected | 1 | NA | 818 | NA | 3815 | 0.3 (0.189 - 0.411) | 1 | NA | 818 | NA | 3815 | 0.3 (0.189 - 0.411) |
| E | Comorbidity | Mean comorbidity score | N America | Unselected | 1 | NA | 77 | NA | 1036 | 0.6 (0.299 - 0.901) | 1 | NA | 77 | NA | 1036 | 0.6 (0.299 - 0.901) |
| E | Comorbidity | Mean comorbidity score | Europe and Central Asia | Admin / registries | 1 | NA | 766 | NA | 10444 | 0.4 (0.269 - 0.531) | 1 | NA | 766 | NA | 10444 | 0.4 (0.269 - 0.531) |
| E | Comorbidity | Mean comorbidity score | Middle East and N Africa | Admin / registries | 1 | NA | 818 | NA | 3815 | 0.3 (0.189 - 0.411) | 1 | NA | 818 | NA | 3815 | 0.3 (0.189 - 0.411) |
| E | Comorbidity | Mean comorbidity score | E Asia and Pacific | High risk | 1 | NA | 32 | NA | 173 | 1 (0.644 - 1.356) | 1 | NA | 32 | NA | 173 | 1 (0.644 - 1.356) |
| E | Comorbidity | Mean comorbidity score | Europe and Central Asia | High risk | 1 | NA | 88 | NA | 84 | 0 (-0.688 - 0.688) | 1 | NA | 88 | NA | 84 | 0 (-0.688 - 0.688) |
| F | Dementia | Dementia diagnosis | All | Unselected | 11 | 7825 | 26028 | 83577 | 324116 | 1.61 (1.26 - 2.05) | 6 | 199 | 1099 | 908 | 6143 | 1.49 (1.26 - 1.77) |
| F | Dementia | Dementia diagnosis | All | Admin / registries | 6 | 7738 | 25779 | 82792 | 320911 | 1.44 (1.04 - 2) | 6 | 7738 | 25779 | 82792 | 320911 | 1.44 (1.04 - 2) |
| F | Dementia | Dementia diagnosis | All | High risk | 3 | 131 | 183 | 396 | 1280 | 1.31 (0.7 - 2.44) | 1 | 0 | 4 | 4 | 8 | 0.21 (0.01 - 3.09) |
| F | Dementia | Dementia diagnosis | E Asia and Pacific | Unselected | 3 | 2324 | 10384 | 16751 | 59896 | 1.068 (0.706 - 1.615) | 2 | 21 | 77 | 271 | 833 | 1.368 (0.946 - 1.979) |
| F | Dementia | Dementia diagnosis | Europe and Central Asia | Unselected | 3 | 1357 | 4284 | 15105 | 103525 | 2.23 (1.879 - 2.647) | 1 | 9 | 22 | 229 | 1229 | 2.196 (1.311 - 3.677) |
| F | Dementia | Dementia diagnosis | Middle East and N Africa | Unselected | 1 | 97 | 818 | 348 | 3815 | 1.3 (1.052 - 1.607) | 1 | 97 | 818 | 348 | 3815 | 1.3 (1.052 - 1.607) |
| F | Dementia | Dementia diagnosis | N America | Unselected | 3 | 4037 | 10469 | 51371 | 156773 | 1.579 (1.078 - 2.312) | 1 | 62 | 109 | 58 | 159 | 1.559 (1.2 - 2.027) |
| F | Dementia | Dementia diagnosis | S Asia | Unselected | 1 | 10 | 73 | 2 | 107 | 7.329 (1.654 - 32.478) | 1 | 10 | 73 | 2 | 107 | 7.329 (1.654 - 32.478) |
| F | Dementia | Dementia diagnosis | E Asia and Pacific | Admin / registries | 1 | 2303 | 10307 | 16480 | 59063 | 0.801 (0.771 - 0.832) | 1 | 2303 | 10307 | 16480 | 59063 | 0.801 (0.771 - 0.832) |
| F | Dementia | Dementia diagnosis | Europe and Central Asia | Admin / registries | 2 | 1348 | 4262 | 14876 | 102296 | 2.227 (1.816 - 2.731) | 2 | 1348 | 4262 | 14876 | 102296 | 2.227 (1.816 - 2.731) |
| F | Dementia | Dementia diagnosis | Middle East and N Africa | Admin / registries | 1 | 97 | 818 | 348 | 3815 | 1.3 (1.052 - 1.607) | 1 | 97 | 818 | 348 | 3815 | 1.3 (1.052 - 1.607) |
| F | Dementia | Dementia diagnosis | N America | Admin / registries | 2 | 3990 | 10392 | 51088 | 155737 | 1.307 (0.988 - 1.729) | 2 | 3990 | 10392 | 51088 | 155737 | 1.307 (0.988 - 1.729) |
| F | Dementia | Dementia diagnosis | Europe and Central Asia | High risk | 3 | 131 | 183 | 396 | 1280 | 1.306 (0.698 - 2.444) | 1 | 0 | 4 | 4 | 8 | 0.21 (0.014 - 3.089) |
| G | Malignancy | Cancer diagnosis | All | Unselected | 6 | 229 | 1868 | 1434 | 17176 | 1.68 (1.35 - 2.1) | 6 | 229 | 1868 | 1434 | 17176 | 1.68 (1.35 - 2.1) |
| G | Malignancy | Cancer diagnosis | All | Admin / registries | 3 | 218 | 1693 | 1176 | 14418 | 1.69 (1.3 - 2.19) | 3 | 218 | 1693 | 1176 | 14418 | 1.69 (1.3 - 2.19) |
| G | Malignancy | Cancer diagnosis | E Asia and Pacific | Unselected | 2 | 6 | 153 | 79 | 1530 | 1.421 (0.49 - 4.116) | 2 | 6 | 153 | 79 | 1530 | 1.421 (0.49 - 4.116) |
| G | Malignancy | Cancer diagnosis | Europe and Central Asia | Unselected | 2 | 156 | 788 | 1221 | 11672 | 1.958 (1.684 - 2.278) | 2 | 156 | 788 | 1221 | 11672 | 1.958 (1.684 - 2.278) |
| G | Malignancy | Cancer diagnosis | Middle East and N Africa | Unselected | 1 | 34 | 818 | 96 | 3815 | 1.652 (1.125 - 2.425) | 1 | 34 | 818 | 96 | 3815 | 1.652 (1.125 - 2.425) |
| G | Malignancy | Cancer diagnosis | N America | Unselected | 1 | 33 | 109 | 38 | 159 | 1.267 (0.851 - 1.885) | 1 | 33 | 109 | 38 | 159 | 1.267 (0.851 - 1.885) |
| G | Malignancy | Cancer diagnosis | Europe and Central Asia | Admin / registries | 1 | 151 | 766 | 1042 | 10444 | 1.976 (1.694 - 2.305) | 1 | 151 | 766 | 1042 | 10444 | 1.976 (1.694 - 2.305) |
| G | Malignancy | Cancer diagnosis | Middle East and N Africa | Admin / registries | 1 | 34 | 818 | 96 | 3815 | 1.652 (1.125 - 2.425) | 1 | 34 | 818 | 96 | 3815 | 1.652 (1.125 - 2.425) |
| G | Malignancy | Cancer diagnosis | N America | Admin / registries | 1 | 33 | 109 | 38 | 159 | 1.267 (0.851 - 1.885) | 1 | 33 | 109 | 38 | 159 | 1.267 (0.851 - 1.885) |
| H | Admission source | Admitted from home | All | Unselected | 9 | 1660 | 4331 | 62238 | 109261 | 0.84 (0.68 - 1.04) | 7 | 857 | 1189 | 11486 | 16360 | 0.98 (0.88 - 1.1) |
| H | Admission source | Admitted from home | All | Admin / registries | 6 | 1546 | 4154 | 60650 | 106731 | 0.84 (0.65 - 1.09) | 5 | 1284 | 3827 | 58042 | 102989 | 0.79 (0.6 - 1.04) |
| H | Admission source | Admitted from home | All | High risk | 3 | 20202 | 23250 | 29684 | 34422 | 0.85 (0.38 - 1.9) | 1 | 20174 | 23155 | 28958 | 33226 | 1 (0.99 - 1.01) |
| H | Admission source | Admitted from home | Europe and Central Asia | Unselected | 3 | 863 | 3165 | 50990 | 93359 | 0.793 (0.453 - 1.391) | 2 | 87 | 100 | 913 | 1494 | 1.131 (1.058 - 1.21) |
| H | Admission source | Admitted from home | N America | Unselected | 6 | 797 | 1166 | 11248 | 15902 | 0.885 (0.737 - 1.061) | 5 | 770 | 1089 | 10573 | 14866 | 0.951 (0.836 - 1.083) |
| H | Admission source | Admitted from home | Europe and Central Asia | Admin / registries | 1 | 776 | 3065 | 50077 | 91865 | 0.464 (0.437 - 0.494) | 1 | 776 | 3065 | 50077 | 91865 | 0.464 (0.437 - 0.494) |
| H | Admission source | Admitted from home | N America | Admin / registries | 5 | 770 | 1089 | 10573 | 14866 | 0.951 (0.836 - 1.083) | 4 | 508 | 762 | 7965 | 11124 | 0.906 (0.812 - 1.01) |
| H | Admission source | Admitted from home | Europe and Central Asia | High risk | 2 | 28 | 183 | 726 | 1280 | 1.06 (0.138 - 8.163) | 0 | 0 | 88 | 0 | 84 | NA (NA - NA) |
| H | Admission source | Admitted from home | N America | High risk | 1 | 20174 | 23067 | 28958 | 33142 | 1.001 (0.995 - 1.007) | 1 | 20174 | 23067 | 28958 | 33142 | 1.001 (0.995 - 1.007) |
| I | Mobility | Independently mobile | All | Unselected | 6 | 755 | 3349 | 46387 | 96187 | 0.77 (0.56 - 1.05) | 5 | 713 | 3290 | 46306 | 96049 | 0.7 (0.5 - 0.96) |
| I | Mobility | Independently mobile | All | Admin / registries | 4 | 696 | 3268 | 45244 | 94821 | 0.65 (0.45 - 0.95) | 4 | 696 | 3268 | 45244 | 94821 | 0.65 (0.45 - 0.95) |
| I | Mobility | Independently mobile | All | High risk | 6 | 111 | 672 | 1541 | 4088 | 0.54 (0.33 - 0.87) | 6 | 111 | 672 | 1541 | 4088 | 0.54 (0.33 - 0.87) |
| I | Mobility | Independently mobile | E Asia and Pacific | Unselected | 3 | 77 | 153 | 1892 | 2935 | 0.854 (0.578 - 1.262) | 2 | 35 | 94 | 1811 | 2797 | 0.697 (0.545 - 0.891) |
| I | Mobility | Independently mobile | Europe and Central Asia | Unselected | 2 | 617 | 3087 | 44403 | 93093 | 0.604 (0.285 - 1.281) | 2 | 617 | 3087 | 44403 | 93093 | 0.604 (0.285 - 1.281) |
| I | Mobility | Independently mobile | N America | Unselected | 1 | 61 | 109 | 92 | 159 | 0.967 (0.782 - 1.197) | 1 | 61 | 109 | 92 | 159 | 0.967 (0.782 - 1.197) |
| I | Mobility | Independently mobile | E Asia and Pacific | Admin / registries | 2 | 35 | 94 | 1811 | 2797 | 0.697 (0.545 - 0.891) | 2 | 35 | 94 | 1811 | 2797 | 0.697 (0.545 - 0.891) |
| I | Mobility | Independently mobile | Europe and Central Asia | Admin / registries | 1 | 600 | 3065 | 43341 | 91865 | 0.415 (0.386 - 0.446) | 1 | 600 | 3065 | 43341 | 91865 | 0.415 (0.386 - 0.446) |
| I | Mobility | Independently mobile | N America | Admin / registries | 1 | 61 | 109 | 92 | 159 | 0.967 (0.782 - 1.197) | 1 | 61 | 109 | 92 | 159 | 0.967 (0.782 - 1.197) |
| I | Mobility | Independently mobile | E Asia and Pacific | High risk | 2 | 10 | 21 | 142 | 182 | 0.715 (0.37 - 1.38) | 2 | 10 | 21 | 142 | 182 | 0.715 (0.37 - 1.38) |
| I | Mobility | Independently mobile | Europe and Central Asia | High risk | 2 | 50 | 179 | 558 | 1272 | 0.451 (0.128 - 1.59) | 2 | 50 | 179 | 558 | 1272 | 0.451 (0.128 - 1.59) |
| I | Mobility | Independently mobile | Middle East and N Africa | High risk | 1 | 2 | 4 | 11 | 19 | 0.864 (0.302 - 2.474) | 1 | 2 | 4 | 11 | 19 | 0.864 (0.302 - 2.474) |
| I | Mobility | Independently mobile | N America | High risk | 1 | 49 | 468 | 830 | 2615 | 0.33 (0.252 - 0.432) | 1 | 49 | 468 | 830 | 2615 | 0.33 (0.252 - 0.432) |
| J | ADLs | Independent of ADLs | All | Unselected | 1 | 680 | 3065 | 50494 | 91865 | 0.4 (0.38 - 0.43) | 1 | 680 | 3065 | 50494 | 91865 | 0.4 (0.38 - 0.43) |
| J | ADLs | Independent of ADLs | All | Admin / registries | 1 | 680 | 3065 | 50494 | 91865 | 0.4 (0.38 - 0.43) | 1 | 680 | 3065 | 50494 | 91865 | 0.4 (0.38 - 0.43) |
| J | ADLs | Independent of ADLs | All | High risk | 2 | 347 | 556 | 2470 | 2699 | 0.79 (0.74 - 0.83) | 2 | 347 | 556 | 2470 | 2699 | 0.79 (0.74 - 0.83) |
| J | ADLs | Independent of ADLs | Europe and Central Asia | Unselected | 1 | 680 | 3065 | 50494 | 91865 | 0.404 (0.378 - 0.431) | 1 | 680 | 3065 | 50494 | 91865 | 0.404 (0.378 - 0.431) |
| J | ADLs | Independent of ADLs | Europe and Central Asia | Admin / registries | 1 | 680 | 3065 | 50494 | 91865 | 0.404 (0.378 - 0.431) | 1 | 680 | 3065 | 50494 | 91865 | 0.404 (0.378 - 0.431) |
| J | ADLs | Independent of ADLs | Europe and Central Asia | High risk | 1 | 0 | 88 | 2 | 84 | 0.191 (0.009 - 3.92) | 1 | 0 | 88 | 2 | 84 | 0.191 (0.009 - 3.92) |
| J | ADLs | Independent of ADLs | N America | High risk | 1 | 347 | 468 | 2468 | 2615 | 0.786 (0.744 - 0.829) | 1 | 347 | 468 | 2468 | 2615 | 0.786 (0.744 - 0.829) |
| K | Ethnicity | Majority ethnicity | All | Unselected | 5 | 12320 | 13944 | 272120 | 293393 | 0.98 (0.95 - 1.01) | 4 | 12267 | 13885 | 272000 | 293265 | 0.98 (0.94 - 1.02) |
| K | Ethnicity | Majority ethnicity | All | Admin / registries | 2 | 12226 | 13633 | 271880 | 292423 | 0.98 (0.94 - 1.02) | 2 | 12226 | 13633 | 271880 | 292423 | 0.98 (0.94 - 1.02) |
| K | Ethnicity | Majority ethnicity | All | High risk | 2 | 589 | 783 | 4867 | 6457 | 0.94 (0.88 - 1.01) | 2 | 589 | 783 | 4867 | 6457 | 0.94 (0.88 - 1.01) |
| K | Ethnicity | Majority ethnicity | E Asia and Pacific | Unselected | 3 | 94 | 311 | 240 | 970 | 0.982 (0.863 - 1.116) | 2 | 41 | 252 | 120 | 842 | 1.135 (0.821 - 1.569) |
| K | Ethnicity | Majority ethnicity | N America | Unselected | 2 | 12226 | 13633 | 271880 | 292423 | 0.979 (0.942 - 1.017) | 2 | 12226 | 13633 | 271880 | 292423 | 0.979 (0.942 - 1.017) |
| K | Ethnicity | Majority ethnicity | N America | Admin / registries | 2 | 12226 | 13633 | 271880 | 292423 | 0.979 (0.942 - 1.017) | 2 | 12226 | 13633 | 271880 | 292423 | 0.979 (0.942 - 1.017) |
| K | Ethnicity | Majority ethnicity | N America | High risk | 2 | 589 | 783 | 4867 | 6457 | 0.942 (0.88 - 1.01) | 2 | 589 | 783 | 4867 | 6457 | 0.942 (0.88 - 1.01) |
| L | Fracture type | Intracapsular fracture | All | Unselected | 26 | 109116 | 346749 | 775714 | 1977783 | 0.97 (0.75 - 1.27) | 24 | 108881 | 345397 | 774309 | 1974834 | 1.05 (0.95 - 1.16) |
| L | Fracture type | Intracapsular fracture | All | Admin / registries | 12 | 108216 | 344124 | 771125 | 1967318 | 1.02 (0.9 - 1.16) | 10 | 21261 | 40666 | 346765 | 769424 | 1.01 (0.9 - 1.13) |
| L | Fracture type | Intracapsular fracture | All | High risk | 11 | 251 | 392 | 910 | 1843 | 1.45 (1.12 - 1.88) | 8 | 154 | 284 | 757 | 1490 | 1.31 (1.01 - 1.69) |
| L | Fracture type | Intracapsular fracture | E Asia and Pacific | Unselected | 11 | 11737 | 23859 | 169770 | 374838 | 1.191 (0.95 - 1.493) | 10 | 11524 | 23528 | 169593 | 374116 | 1.075 (0.94 - 1.229) |
| L | Fracture type | Intracapsular fracture | Europe and Central Asia | Unselected | 7 | 89088 | 308094 | 476697 | 1294624 | 0.672 (0.264 - 1.709) | 6 | 89066 | 307073 | 475469 | 1292397 | 1.077 (0.846 - 1.371) |
| L | Fracture type | Intracapsular fracture | N America | Unselected | 8 | 8291 | 14796 | 129247 | 308321 | 1 (0.86 - 1.162) | 8 | 8291 | 14796 | 129247 | 308321 | 1 (0.86 - 1.162) |
| L | Fracture type | Intracapsular fracture | E Asia and Pacific | Admin / registries | 2 | 11218 | 22882 | 168039 | 370274 | 1.069 (1.005 - 1.138) | 2 | 11218 | 22882 | 168039 | 370274 | 1.069 (1.005 - 1.138) |
| L | Fracture type | Intracapsular fracture | Europe and Central Asia | Admin / registries | 3 | 88751 | 306523 | 474365 | 1289759 | 1.097 (0.767 - 1.568) | 1 | 1796 | 3065 | 50005 | 91865 | 1.076 (1.044 - 1.11) |
| L | Fracture type | Intracapsular fracture | N America | Admin / registries | 7 | 8247 | 14719 | 128721 | 307285 | 0.984 (0.832 - 1.163) | 7 | 8247 | 14719 | 128721 | 307285 | 0.984 (0.832 - 1.163) |
| L | Fracture type | Intracapsular fracture | E Asia and Pacific | High risk | 3 | 100 | 122 | 117 | 252 | 1.273 (0.82 - 1.975) | 1 | 11 | 23 | 19 | 42 | 1.057 (0.615 - 1.816) |
| L | Fracture type | Intracapsular fracture | Europe and Central Asia | High risk | 7 | 149 | 266 | 791 | 1572 | 1.51 (1.063 - 2.145) | 6 | 141 | 257 | 736 | 1429 | 1.327 (0.973 - 1.809) |
| L | Fracture type | Intracapsular fracture | Middle East and N Africa | High risk | 1 | 2 | 4 | 2 | 19 | 4.75 (0.924 - 24.407) | 1 | 2 | 4 | 2 | 19 | 4.75 (0.924 - 24.407) |

### **Table S31:** Sensitivity analyses for outcomes (mortality, length of hospital stay, ambulatory status at discharge, delirium and complications). Results are reported by cohort type, region and with outliers removed.

| **Comparison** | **Metric** | **Event** | **Group** | **Cohort** | **Number of cohorts** | **Events conservative** | **Total conservative** | **Events operative** | **Total operative** | **Estimate - all** | **Number of cohorts*** | **Events conservative*** | **Total conservative*** | **Events operative*** | **Total operative*** | **Estimate*** |
| --- | --- | --- | --- | --- | --- | --- | --- | --- | --- | --- | --- | --- | --- | --- | --- | --- |
| M1 | Mortality: in hospital | Death in hospital | All | Unselected | 30 | 3204 | 25482 | 9774 | 255766 | 5.57 (4.41 - 7.02) | 26 | 1771 | 10705 | 8259 | 183925 | 4.95 (4.13 - 5.92) |
| M1 | Mortality: in hospital | Death in hospital | All | Admin / registries | 18 | 3080 | 24518 | 9588 | 249332 | 4.72 (3.6 - 6.19) | 16 | 2960 | 23654 | 9454 | 244522 | 4.21 (3.58 - 4.94) |
| M1 | Mortality: in hospital | Death in hospital | All | High risk | 4 | 136 | 854 | 194 | 3392 | 2.93 (2.4 - 3.58) | 4 | 136 | 854 | 194 | 3392 | 2.93 (2.4 - 3.58) |
| M1 | Mortality: in hospital | Death in hospital | E Asia and Pacific | Unselected | 6 | 1336 | 14316 | 1396 | 68645 | 5.498 (3.458 - 8.74) | 4 | 23 | 403 | 15 | 1614 | 5.411 (1.597 - 18.336) |
| M1 | Mortality: in hospital | Death in hospital | Europe and Central Asia | Unselected | 8 | 791 | 1982 | 4076 | 64051 | 8.233 (5.938 - 11.416) | 8 | 791 | 1982 | 4076 | 64051 | 8.233 (5.938 - 11.416) |
| M1 | Mortality: in hospital | Death in hospital | Latin America & the Caribbean | Unselected | 3 | 484 | 4644 | 2445 | 64242 | 6.834 (1.049 - 44.496) | 2 | 453 | 4598 | 2431 | 63247 | 2.653 (2.409 - 2.921) |
| M1 | Mortality: in hospital | Death in hospital | Middle East and N Africa | Unselected | 11 | 332 | 3124 | 1020 | 40350 | 4.165 (3.691 - 4.7) | 10 | 243 | 2306 | 900 | 36535 | 4.376 (3.819 - 5.013) |
| M1 | Mortality: in hospital | Death in hospital | N America | Unselected | 2 | 261 | 1416 | 837 | 18478 | 4.224 (3.714 - 4.805) | 2 | 261 | 1416 | 837 | 18478 | 4.224 (3.714 - 4.805) |
| M1 | Mortality: in hospital | Death in hospital | E Asia and Pacific | Admin / registries | 1 | 1309 | 13907 | 1372 | 66893 | 4.589 (4.264 - 4.939) | 1 | 1309 | 13907 | 1372 | 66893 | 4.589 (4.264 - 4.939) |
| M1 | Mortality: in hospital | Death in hospital | Europe and Central Asia | Admin / registries | 1 | 694 | 1427 | 3914 | 59369 | 7.377 (6.938 - 7.843) | 1 | 694 | 1427 | 3914 | 59369 | 7.377 (6.938 - 7.843) |
| M1 | Mortality: in hospital | Death in hospital | Latin America & the Caribbean | Admin / registries | 3 | 484 | 4644 | 2445 | 64242 | 6.834 (1.049 - 44.496) | 2 | 453 | 4598 | 2431 | 63247 | 2.653 (2.409 - 2.921) |
| M1 | Mortality: in hospital | Death in hospital | Middle East and N Africa | Admin / registries | 11 | 332 | 3124 | 1020 | 40350 | 4.165 (3.691 - 4.7) | 10 | 243 | 2306 | 900 | 36535 | 4.376 (3.819 - 5.013) |
| M1 | Mortality: in hospital | Death in hospital | N America | Admin / registries | 2 | 261 | 1416 | 837 | 18478 | 4.224 (3.714 - 4.805) | 2 | 261 | 1416 | 837 | 18478 | 4.224 (3.714 - 4.805) |
| M1 | Mortality: in hospital | Death in hospital | E Asia and Pacific | High risk | 1 | 129 | 839 | 170 | 3220 | 2.912 (2.347 - 3.614) | 1 | 129 | 839 | 170 | 3220 | 2.912 (2.347 - 3.614) |
| M1 | Mortality: in hospital | Death in hospital | Europe and Central Asia | High risk | 3 | 7 | 15 | 24 | 172 | 3.049 (1.823 - 5.1) | 3 | 7 | 15 | 24 | 172 | 3.049 (1.823 - 5.1) |
| M2 | Mortality: 7 day | Death at 7 days | All | Unselected | 1 | 1933 | 10283 | 2489 | 155578 | 11.75 (11.11 - 12.43) | 1 | 1933 | 10283 | 2489 | 155578 | 11.75 (11.11 - 12.43) |
| M2 | Mortality: 7 day | Death at 7 days | All | Admin / registries | 1 | 1933 | 10283 | 2489 | 155578 | 11.75 (11.11 - 12.43) | 1 | 1933 | 10283 | 2489 | 155578 | 11.75 (11.11 - 12.43) |
| M2 | Mortality: 7 day | Death at 7 days | All | High risk | 2 | 4 | 90 | 3 | 105 | 6 (2.41 - 14.97) | 2 | 4 | 90 | 3 | 105 | 6 (2.41 - 14.97) |
| M2 | Mortality: 7 day | Death at 7 days | N America | Unselected | 1 | 1933 | 10283 | 2489 | 155578 | 11.75 (11.11 - 12.426) | 1 | 1933 | 10283 | 2489 | 155578 | 11.75 (11.11 - 12.426) |
| M2 | Mortality: 7 day | Death at 7 days | N America | Admin / registries | 1 | 1933 | 10283 | 2489 | 155578 | 11.75 (11.11 - 12.426) | 1 | 1933 | 10283 | 2489 | 155578 | 11.75 (11.11 - 12.426) |
| M2 | Mortality: 7 day | Death at 7 days | Europe and Central Asia | High risk | 2 | 4 | 90 | 3 | 105 | 6.003 (2.407 - 14.969) | 2 | 4 | 90 | 3 | 105 | 6.003 (2.407 - 14.969) |
| M3 | Mortality: 30 day | Death at 30 days | All | Unselected | 17 | 26883 | 145363 | 32510 | 429267 | 3.78 (3.1 - 4.63) | 14 | 4473 | 14504 | 12059 | 182913 | 4.08 (3.45 - 4.83) |
| M3 | Mortality: 30 day | Death at 30 days | All | Admin / registries | 8 | 26662 | 143760 | 32219 | 422376 | 3.24 (2.52 - 4.16) | 7 | 25182 | 136136 | 28264 | 358080 | 3.25 (2.43 - 4.35) |
| M3 | Mortality: 30 day | Death at 30 days | All | High risk | 11 | 889 | 23465 | 3347 | 34846 | 2.4 (1.18 - 4.89) | 8 | 798 | 23275 | 3258 | 33448 | 1.75 (0.82 - 3.74) |
| M3 | Mortality: 30 day | Death at 30 days | All | Matched | 4 | 117 | 320 | 42 | 527 | 4.5 (1.87 - 10.85) | 4 | 117 | 320 | 42 | 527 | 4.5 (1.87 - 10.85) |
| M3 | Mortality: 30 day | Death at 30 days | E Asia and Pacific | Unselected | 5 | 20996 | 124043 | 16538 | 184433 | 4.188 (1.877 - 9.343) | 3 | 66 | 808 | 42 | 2375 | 4.482 (3.057 - 6.572) |
| M3 | Mortality: 30 day | Death at 30 days | Europe and Central Asia | Unselected | 9 | 1986 | 10110 | 5216 | 85282 | 3.803 (3.188 - 4.536) | 8 | 506 | 2486 | 1261 | 20986 | 4.005 (3.255 - 4.928) |
| M3 | Mortality: 30 day | Death at 30 days | Middle East and N Africa | Unselected | 1 | 109 | 818 | 169 | 3815 | 3.008 (2.393 - 3.781) | 1 | 109 | 818 | 169 | 3815 | 3.008 (2.393 - 3.781) |
| M3 | Mortality: 30 day | Death at 30 days | N America | Unselected | 2 | 3792 | 10392 | 10587 | 155737 | 5.19 (4.06 - 6.635) | 2 | 3792 | 10392 | 10587 | 155737 | 5.19 (4.06 - 6.635) |
| M3 | Mortality: 30 day | Death at 30 days | E Asia and Pacific | Admin / registries | 1 | 20910 | 123002 | 16496 | 181277 | 1.868 (1.833 - 1.904) | 1 | 20910 | 123002 | 16496 | 181277 | 1.868 (1.833 - 1.904) |
| M3 | Mortality: 30 day | Death at 30 days | Europe and Central Asia | Admin / registries | 4 | 1851 | 9548 | 4967 | 81547 | 3.375 (3.032 - 3.757) | 3 | 371 | 1924 | 1012 | 17251 | 3.619 (3.236 - 4.046) |
| M3 | Mortality: 30 day | Death at 30 days | Middle East and N Africa | Admin / registries | 1 | 109 | 818 | 169 | 3815 | 3.008 (2.393 - 3.781) | 1 | 109 | 818 | 169 | 3815 | 3.008 (2.393 - 3.781) |
| M3 | Mortality: 30 day | Death at 30 days | N America | Admin / registries | 2 | 3792 | 10392 | 10587 | 155737 | 5.19 (4.06 - 6.635) | 2 | 3792 | 10392 | 10587 | 155737 | 5.19 (4.06 - 6.635) |
| M3 | Mortality: 30 day | Death at 30 days | E Asia and Pacific | High risk | 3 | 14 | 137 | 7 | 256 | 2.597 (1.058 - 6.375) | 1 | 2 | 38 | 1 | 46 | 2.421 (0.228 - 25.685) |
| M3 | Mortality: 30 day | Death at 30 days | Europe and Central Asia | High risk | 6 | 173 | 257 | 135 | 1429 | 2.917 (1.387 - 6.137) | 5 | 94 | 166 | 52 | 241 | 2.252 (1.439 - 3.523) |
| M3 | Mortality: 30 day | Death at 30 days | Middle East and N Africa | High risk | 1 | 3 | 4 | 1 | 19 | 14.25 (1.948 - 104.228) | 1 | 3 | 4 | 1 | 19 | 14.25 (1.948 - 104.228) |
| M3 | Mortality: 30 day | Death at 30 days | N America | High risk | 1 | 699 | 23067 | 3204 | 33142 | 0.313 (0.289 - 0.34) | 1 | 699 | 23067 | 3204 | 33142 | 0.313 (0.289 - 0.34) |
| M3 | Mortality: 30 day | Death at 30 days | Europe and Central Asia | Matched | 1 | 4 | 21 | 1 | 20 | 3.81 (0.465 - 31.234) | 1 | 4 | 21 | 1 | 20 | 3.81 (0.465 - 31.234) |
| M3 | Mortality: 30 day | Death at 30 days | Middle East and N Africa | Matched | 1 | 24 | 94 | 17 | 114 | 1.712 (0.98 - 2.992) | 1 | 24 | 94 | 17 | 114 | 1.712 (0.98 - 2.992) |
| M3 | Mortality: 30 day | Death at 30 days | N America | Matched | 2 | 89 | 205 | 24 | 393 | 7.254 (4.047 - 13.002) | 2 | 89 | 205 | 24 | 393 | 7.254 (4.047 - 13.002) |
| M4 | Mortality: 90 day | Death at 90 days | All | Unselected | 4 | 2487 | 8553 | 8648 | 75849 | 2.63 (2.51 - 2.75) | 4 | 2487 | 8553 | 8648 | 75849 | 2.63 (2.51 - 2.75) |
| M4 | Mortality: 90 day | Death at 90 days | All | Admin / registries | 3 | 2483 | 8532 | 8645 | 75750 | 2.62 (2.51 - 2.74) | 3 | 2483 | 8532 | 8645 | 75750 | 2.62 (2.51 - 2.74) |
| M4 | Mortality: 90 day | Death at 90 days | All | High risk | 6 | 5699 | 23669 | 7323 | 35975 | 1.84 (1.33 - 2.54) | 4 | 5603 | 23541 | 7257 | 35797 | 1.96 (1.24 - 3.08) |
| M4 | Mortality: 90 day | Death at 90 days | E Asia and Pacific | Unselected | 2 | 27 | 163 | 59 | 1109 | 3.149 (2.015 - 4.919) | 2 | 27 | 163 | 59 | 1109 | 3.149 (2.015 - 4.919) |
| M4 | Mortality: 90 day | Death at 90 days | Europe and Central Asia | Unselected | 2 | 2460 | 8390 | 8589 | 74740 | 2.617 (2.505 - 2.734) | 2 | 2460 | 8390 | 8589 | 74740 | 2.617 (2.505 - 2.734) |
| M4 | Mortality: 90 day | Death at 90 days | E Asia and Pacific | Admin / registries | 1 | 23 | 142 | 56 | 1010 | 2.921 (1.858 - 4.593) | 1 | 23 | 142 | 56 | 1010 | 2.921 (1.858 - 4.593) |
| M4 | Mortality: 90 day | Death at 90 days | Europe and Central Asia | Admin / registries | 2 | 2460 | 8390 | 8589 | 74740 | 2.617 (2.505 - 2.734) | 2 | 2460 | 8390 | 8589 | 74740 | 2.617 (2.505 - 2.734) |
| M4 | Mortality: 90 day | Death at 90 days | Europe and Central Asia | High risk | 3 | 98 | 130 | 74 | 199 | 1.903 (1.225 - 2.955) | 1 | 2 | 2 | 8 | 21 | 2.529 (1.5 - 4.266) |
| M4 | Mortality: 90 day | Death at 90 days | Middle East and N Africa | High risk | 1 | 4 | 4 | 5 | 19 | 3.545 (1.746 - 7.198) | 1 | 4 | 4 | 5 | 19 | 3.545 (1.746 - 7.198) |
| M4 | Mortality: 90 day | Death at 90 days | N America | High risk | 2 | 5597 | 23535 | 7244 | 35757 | 1.503 (0.904 - 2.498) | 2 | 5597 | 23535 | 7244 | 35757 | 1.503 (0.904 - 2.498) |
| M6 | Mortality: 1 year | Death at 1 year | All | Unselected | 31 | 10345 | 23233 | 41240 | 175268 | 2.77 (2.37 - 3.25) | 30 | 10323 | 23197 | 41177 | 174668 | 2.69 (2.31 - 3.14) |
| M6 | Mortality: 1 year | Death at 1 year | All | Admin / registries | 11 | 9406 | 21226 | 39096 | 162574 | 2.03 (1.67 - 2.46) | 8 | 5388 | 11892 | 17852 | 68424 | 1.85 (1.54 - 2.22) |
| M6 | Mortality: 1 year | Death at 1 year | All | High risk | 13 | 579 | 1099 | 2442 | 8283 | 1.71 (1.37 - 2.14) | 11 | 483 | 1002 | 2087 | 7039 | 1.46 (1.37 - 1.56) |
| M6 | Mortality: 1 year | Death at 1 year | All | Matched | 5 | 212 | 348 | 167 | 583 | 2.17 (1.49 - 3.16) | 5 | 212 | 348 | 167 | 583 | 2.17 (1.49 - 3.16) |
| M6 | Mortality: 1 year | Death at 1 year | E Asia and Pacific | Unselected | 15 | 1331 | 2554 | 6107 | 18340 | 3.038 (2.342 - 3.941) | 15 | 1331 | 2554 | 6107 | 18340 | 3.038 (2.342 - 3.941) |
| M6 | Mortality: 1 year | Death at 1 year | Europe and Central Asia | Unselected | 9 | 4827 | 10690 | 24879 | 107857 | 2.7 (2.092 - 3.485) | 8 | 4805 | 10654 | 24816 | 107257 | 2.445 (2.018 - 2.964) |
| M6 | Mortality: 1 year | Death at 1 year | Latin America & the Caribbean | Unselected | 2 | 3267 | 7678 | 5289 | 26537 | 2.501 (1.771 - 3.534) | 2 | 3267 | 7678 | 5289 | 26537 | 2.501 (1.771 - 3.534) |
| M6 | Mortality: 1 year | Death at 1 year | Middle East and N Africa | Unselected | 1 | 296 | 818 | 742 | 3815 | 1.86 (1.664 - 2.08) | 1 | 296 | 818 | 742 | 3815 | 1.86 (1.664 - 2.08) |
| M6 | Mortality: 1 year | Death at 1 year | N America | Unselected | 2 | 596 | 1416 | 4209 | 18478 | 1.834 (1.713 - 1.964) | 2 | 596 | 1416 | 4209 | 18478 | 1.834 (1.713 - 1.964) |
| M6 | Mortality: 1 year | Death at 1 year | S Asia | Unselected | 2 | 28 | 77 | 14 | 241 | 6.099 (2.858 - 13.014) | 2 | 28 | 77 | 14 | 241 | 6.099 (2.858 - 13.014) |
| M6 | Mortality: 1 year | Death at 1 year | E Asia and Pacific | Admin / registries | 4 | 932 | 1509 | 5457 | 13145 | 2.07 (1.158 - 3.703) | 3 | 900 | 1449 | 5333 | 12155 | 1.63 (1.002 - 2.65) |
| M6 | Mortality: 1 year | Death at 1 year | Europe and Central Asia | Admin / registries | 3 | 4456 | 10040 | 24000 | 103604 | 2.138 (1.743 - 2.624) | 1 | 470 | 766 | 2880 | 10444 | 2.225 (2.087 - 2.373) |
| M6 | Mortality: 1 year | Death at 1 year | Latin America & the Caribbean | Admin / registries | 1 | 3126 | 7443 | 4688 | 23532 | 2.108 (2.032 - 2.188) | 1 | 3126 | 7443 | 4688 | 23532 | 2.108 (2.032 - 2.188) |
| M6 | Mortality: 1 year | Death at 1 year | Middle East and N Africa | Admin / registries | 1 | 296 | 818 | 742 | 3815 | 1.86 (1.664 - 2.08) | 1 | 296 | 818 | 742 | 3815 | 1.86 (1.664 - 2.08) |
| M6 | Mortality: 1 year | Death at 1 year | N America | Admin / registries | 2 | 596 | 1416 | 4209 | 18478 | 1.834 (1.713 - 1.964) | 2 | 596 | 1416 | 4209 | 18478 | 1.834 (1.713 - 1.964) |
| M6 | Mortality: 1 year | Death at 1 year | E Asia and Pacific | High risk | 5 | 60 | 166 | 70 | 354 | 1.911 (1.374 - 2.66) | 4 | 54 | 160 | 48 | 298 | 1.572 (1.078 - 2.29) |
| M6 | Mortality: 1 year | Death at 1 year | Europe and Central Asia | High risk | 5 | 125 | 146 | 469 | 1453 | 1.624 (1.009 - 2.611) | 4 | 35 | 55 | 136 | 265 | 1.275 (1.08 - 1.506) |
| M6 | Mortality: 1 year | Death at 1 year | Middle East and N Africa | High risk | 1 | 4 | 4 | 8 | 19 | 2.294 (1.385 - 3.801) | 1 | 4 | 4 | 8 | 19 | 2.294 (1.385 - 3.801) |
| M6 | Mortality: 1 year | Death at 1 year | N America | High risk | 2 | 390 | 783 | 1895 | 6457 | 1.486 (1.379 - 1.601) | 2 | 390 | 783 | 1895 | 6457 | 1.486 (1.379 - 1.601) |
| M6 | Mortality: 1 year | Death at 1 year | E Asia and Pacific | Matched | 1 | 18 | 28 | 8 | 56 | 4.5 (2.238 - 9.048) | 1 | 18 | 28 | 8 | 56 | 4.5 (2.238 - 9.048) |
| M6 | Mortality: 1 year | Death at 1 year | Europe and Central Asia | Matched | 1 | 7 | 21 | 5 | 20 | 1.333 (0.505 - 3.519) | 1 | 7 | 21 | 5 | 20 | 1.333 (0.505 - 3.519) |
| M6 | Mortality: 1 year | Death at 1 year | Middle East and N Africa | Matched | 1 | 63 | 94 | 55 | 114 | 1.389 (1.096 - 1.761) | 1 | 63 | 94 | 55 | 114 | 1.389 (1.096 - 1.761) |
| M6 | Mortality: 1 year | Death at 1 year | N America | Matched | 2 | 124 | 205 | 99 | 393 | 2.398 (1.986 - 2.895) | 2 | 124 | 205 | 99 | 393 | 2.398 (1.986 - 2.895) |
| M7 | Mortality: 2 years | Death at 2 years | All | Unselected | 7 | 5556 | 10512 | 34651 | 105564 | 1.97 (1.64 - 2.38) | 5 | 1743 | 2772 | 13729 | 40577 | 2.17 (1.73 - 2.72) |
| M7 | Mortality: 2 years | Death at 2 years | All | Admin / registries | 4 | 5369 | 10149 | 34223 | 103763 | 1.74 (1.56 - 1.95) | 3 | 1627 | 2525 | 13548 | 39467 | 1.86 (1.8 - 1.92) |
| M7 | Mortality: 2 years | Death at 2 years | All | High risk | 6 | 453 | 647 | 1671 | 2985 | 1.27 (1.15 - 1.4) | 6 | 453 | 647 | 1671 | 2985 | 1.27 (1.15 - 1.4) |
| M7 | Mortality: 2 years | Death at 2 years | E Asia and Pacific | Unselected | 3 | 187 | 363 | 428 | 1801 | 2.47 (1.658 - 3.681) | 2 | 116 | 247 | 181 | 1110 | 2.957 (2.429 - 3.599) |
| M7 | Mortality: 2 years | Death at 2 years | Europe and Central Asia | Unselected | 3 | 5300 | 10040 | 34165 | 103604 | 1.742 (1.526 - 1.987) | 2 | 1558 | 2416 | 13490 | 39308 | 1.865 (1.806 - 1.927) |
| M7 | Mortality: 2 years | Death at 2 years | N America | Unselected | 1 | 69 | 109 | 58 | 159 | 1.735 (1.351 - 2.228) | 1 | 69 | 109 | 58 | 159 | 1.735 (1.351 - 2.228) |
| M7 | Mortality: 2 years | Death at 2 years | Europe and Central Asia | Admin / registries | 3 | 5300 | 10040 | 34165 | 103604 | 1.742 (1.526 - 1.987) | 2 | 1558 | 2416 | 13490 | 39308 | 1.865 (1.806 - 1.927) |
| M7 | Mortality: 2 years | Death at 2 years | N America | Admin / registries | 1 | 69 | 109 | 58 | 159 | 1.735 (1.351 - 2.228) | 1 | 69 | 109 | 58 | 159 | 1.735 (1.351 - 2.228) |
| M7 | Mortality: 2 years | Death at 2 years | E Asia and Pacific | High risk | 3 | 63 | 137 | 65 | 256 | 1.46 (1.071 - 1.991) | 3 | 63 | 137 | 65 | 256 | 1.46 (1.071 - 1.991) |
| M7 | Mortality: 2 years | Death at 2 years | Europe and Central Asia | High risk | 2 | 33 | 42 | 71 | 114 | 1.205 (0.981 - 1.481) | 2 | 33 | 42 | 71 | 114 | 1.205 (0.981 - 1.481) |
| M7 | Mortality: 2 years | Death at 2 years | N America | High risk | 1 | 357 | 468 | 1535 | 2615 | 1.3 (1.224 - 1.38) | 1 | 357 | 468 | 1535 | 2615 | 1.3 (1.224 - 1.38) |
| M8 | Mortality: 3 years | Death at 3 years | All | Unselected | 1 | 574 | 766 | 4817 | 10444 | 1.62 (1.55 - 1.7) | 1 | 574 | 766 | 4817 | 10444 | 1.62 (1.55 - 1.7) |
| M8 | Mortality: 3 years | Death at 3 years | All | Admin / registries | 1 | 574 | 766 | 4817 | 10444 | 1.62 (1.55 - 1.7) | 1 | 574 | 766 | 4817 | 10444 | 1.62 (1.55 - 1.7) |
| M8 | Mortality: 3 years | Death at 3 years | All | High risk | 2 | 43 | 69 | 33 | 57 | 1.23 (0.82 - 1.84) | 2 | 43 | 69 | 33 | 57 | 1.23 (0.82 - 1.84) |
| M8 | Mortality: 3 years | Death at 3 years | Europe and Central Asia | Unselected | 1 | 574 | 766 | 4817 | 10444 | 1.625 (1.552 - 1.701) | 1 | 574 | 766 | 4817 | 10444 | 1.625 (1.552 - 1.701) |
| M8 | Mortality: 3 years | Death at 3 years | Europe and Central Asia | Admin / registries | 1 | 574 | 766 | 4817 | 10444 | 1.625 (1.552 - 1.701) | 1 | 574 | 766 | 4817 | 10444 | 1.625 (1.552 - 1.701) |
| M8 | Mortality: 3 years | Death at 3 years | E Asia and Pacific | High risk | 1 | 41 | 67 | 14 | 37 | 1.617 (1.026 - 2.549) | 1 | 41 | 67 | 14 | 37 | 1.617 (1.026 - 2.549) |
| M8 | Mortality: 3 years | Death at 3 years | Europe and Central Asia | High risk | 1 | 2 | 2 | 19 | 20 | 1.051 (0.953 - 1.16) | 1 | 2 | 2 | 19 | 20 | 1.051 (0.953 - 1.16) |
| N | Length of stay | Length of stay (days) | All | Unselected | 4 | NA | 956 | NA | 4116 | -1.74 (-2.4 - -1.07) | 4 | NA | 956 | NA | 4116 | -1.74 (-2.4 - -1.07) |
| N | Length of stay | Length of stay (days) | All | Admin / registries | 2 | NA | 927 | NA | 3974 | -1.77 (-2.44 - -1.11) | 2 | NA | 927 | NA | 3974 | -1.77 (-2.44 - -1.11) |
| N | Length of stay | Length of stay (days) | All | High risk | 5 | NA | 229 | NA | 409 | -6.69 (-12.19 - -1.19) | 3 | NA | 139 | NA | 304 | -4.94 (-11.22 - 1.33) |
| N | Length of stay | Length of stay (days) | E Asia and Pacific | Unselected | 1 | NA | 7 | NA | 62 | 4 (-9.438 - 17.438) | 1 | NA | 7 | NA | 62 | 4 (-9.438 - 17.438) |
| N | Length of stay | Length of stay (days) | Europe and Central Asia | Unselected | 1 | NA | 22 | NA | 80 | 2 (-6.839 - 10.839) | 1 | NA | 22 | NA | 80 | 2 (-6.839 - 10.839) |
| N | Length of stay | Length of stay (days) | Middle East and N Africa | Unselected | 1 | NA | 818 | NA | 3815 | -1.7 (-2.401 - -0.999) | 1 | NA | 818 | NA | 3815 | -1.7 (-2.401 - -0.999) |
| N | Length of stay | Length of stay (days) | N America | Unselected | 1 | NA | 109 | NA | 159 | -2.4 (-4.492 - -0.308) | 1 | NA | 109 | NA | 159 | -2.4 (-4.492 - -0.308) |
| N | Length of stay | Length of stay (days) | Middle East and N Africa | Admin / registries | 1 | NA | 818 | NA | 3815 | -1.7 (-2.401 - -0.999) | 1 | NA | 818 | NA | 3815 | -1.7 (-2.401 - -0.999) |
| N | Length of stay | Length of stay (days) | N America | Admin / registries | 1 | NA | 109 | NA | 159 | -2.4 (-4.492 - -0.308) | 1 | NA | 109 | NA | 159 | -2.4 (-4.492 - -0.308) |
| N | Length of stay | Length of stay (days) | E Asia and Pacific | High risk | 2 | NA | 99 | NA | 210 | -2.375 (-10.716 - 5.966) | 2 | NA | 99 | NA | 210 | -2.375 (-10.716 - 5.966) |
| N | Length of stay | Length of stay (days) | Europe and Central Asia | High risk | 3 | NA | 130 | NA | 199 | -8.732 (-15.994 - -1.471) | 1 | NA | 40 | NA | 94 | -7.8 (-10.93 - -4.67) |
| P | Ambulation | Ambulatory on discharge | All | High risk | 5 | 7 | 86 | 129 | 289 | 0.26 (0.13 - 0.5) | 5 | 7 | 86 | 129 | 289 | 0.26 (0.13 - 0.5) |
| P | Ambulation | Ambulatory on discharge | E Asia and Pacific | High risk | 4 | 7 | 82 | 125 | 270 | 0.246 (0.123 - 0.491) | 4 | 7 | 82 | 125 | 270 | 0.246 (0.123 - 0.491) |
| P | Ambulation | Ambulatory on discharge | Middle East and N Africa | High risk | 1 | 0 | 4 | 4 | 19 | 0.481 (0.031 - 7.427) | 1 | 0 | 4 | 4 | 19 | 0.481 (0.031 - 7.427) |
| Q | Delirium | Delirium | All | Unselected | 1 | 12 | 233 | 86 | 781 | 0.47 (0.26 - 0.84) | 1 | 12 | 233 | 86 | 781 | 0.47 (0.26 - 0.84) |
| Q | Delirium | Delirium | All | High risk | 3 | 3734 | 23222 | 5484 | 33263 | 0.78 (0.4 - 1.52) | 3 | 3734 | 23222 | 5484 | 33263 | 0.78 (0.4 - 1.52) |
| Q | Delirium | Delirium | E Asia and Pacific | Unselected | 1 | 12 | 233 | 86 | 781 | 0.468 (0.26 - 0.84) | 1 | 12 | 233 | 86 | 781 | 0.468 (0.26 - 0.84) |
| Q | Delirium | Delirium | E Asia and Pacific | High risk | 1 | 30 | 67 | 14 | 37 | 1.183 (0.724 - 1.934) | 1 | 30 | 67 | 14 | 37 | 1.183 (0.724 - 1.934) |
| Q | Delirium | Delirium | Europe and Central Asia | High risk | 1 | 10 | 88 | 27 | 84 | 0.354 (0.183 - 0.685) | 1 | 10 | 88 | 27 | 84 | 0.354 (0.183 - 0.685) |
| Q | Delirium | Delirium | N America | High risk | 1 | 3694 | 23067 | 5443 | 33142 | 0.975 (0.938 - 1.013) | 1 | 3694 | 23067 | 5443 | 33142 | 0.975 (0.938 - 1.013) |
| S | Complications | Complications | All | Unselected | 3 | 134 | 914 | 166 | 2362 | 1.82 (0.84 - 3.93) | 3 | 134 | 914 | 166 | 2362 | 1.82 (0.84 - 3.93) |
| S | Complications | Complications | E Asia and Pacific | Unselected | 2 | 97 | 841 | 150 | 2255 | 1.32 (0.541 - 3.225) | 2 | 97 | 841 | 150 | 2255 | 1.32 (0.541 - 3.225) |
| S | Complications | Complications | S Asia | Unselected | 1 | 37 | 73 | 16 | 107 | 3.39 (2.045 - 5.618) | 1 | 37 | 73 | 16 | 107 | 3.39 (2.045 - 5.618) |
| S | Complications | Complications | E Asia and Pacific | High risk | 3 | 48 | 93 | 68 | 261 | 1.582 (1.017 - 2.46) | 1 | 24 | 38 | 20 | 46 | 1.453 (0.965 - 2.187) |
